# Review of the genera of Conoderinae (Coleoptera, Curculionidae) from North America, Central America, and the Caribbean

**DOI:** 10.3897/zookeys.683.12080

**Published:** 2017-07-07

**Authors:** Salvatore S. Anzaldo

**Affiliations:** 1 School of Life Sciences, PO Box 874501, Arizona State University, Tempe, AZ, 85287-4501, USA

**Keywords:** weevils, Neotropical, taxonomy, Zygopinae

## Abstract

The thirty-nine extant genera of Conoderinae known to occur in North America, Central America, and the Caribbean are reviewed based on external morphology. An identification key is provided along with diagnoses, distributions, species counts, and natural history information, when known, for each genus. Morphological character systems of importance for weevil classification are surveyed, potential relationships among the tribes and genera are discussed, and groups most in need of taxonomic and phylogenetic attention are identified. The following genera are transferred to new tribes: *Acoptus* LeConte, 1876 from the Lechriopini to the Othippiini **(new placement)** and the South American genus *Hedycera* Pascoe, 1870 from the Lechriopini to the Piazurini **(new placement)**. *Philides* Champion, 1906 and *Philinna* Champion, 1906 are transferred from the Lechriopini to Conoderinae *incertae sedis* **(new placement)** although their placement as conoderines is uncertain. The species *Copturomimus cinereus* Heller, 1895 is designated as the type species of the genus *Copturomimus* Heller, 1895.

## Introduction

Conoderinae Schoenherr, 1833 (*sensu stricto*, Alonso-Zarazaga and Lyal 1999; Conoderitae *sensu* Prena et al. 2014) are a conspicuous representative of the immensely diverse tropical weevil fauna yet have received scant attention from taxonomists in the last century. Despite being one of the most recognizable subfamilies of Curculionidae Latreille, 1802, the classification of tribes and genera remains chaotic and there are currently no phylogenetic hypotheses for the relationships within Conoderinae. The most recent classification suggests that conoderines are part of a larger group composed of Conoderinae, Baridinae Schoenherr, 1836, Ceutorhynchinae Gistel, 1848, and Orobitidinae Thomson, 1859 (Conoderinae *sensu lato*; Prena et al. 2014), but molecular phylogenies, although to date containing very limited sampling of any of these groups, have not been congruent with this classification (e.g. Gunter et al. 2016).

Much of the published literature treating the Conoderinae, including the descriptions of the majority of genera and species, dates from over a century ago and is relatively inaccessible due to its publication in multiple languages and in a quality that does not meet standards of modern taxonomic practice. Several identification keys for the genera north of Mexico exist (see identification key introduction), but for identifying the far more diverse Central and South American fauna the only keys to genera are by Rheinheimer (2011) for French Guiana and Heller (1895) for the New World. Champion (1906b) described 14 genera from Central America, and since many of those are not also known from French Guiana, Guadeloupe and the United States, and were published after Heller’s 1895 key, they have not been treated in a published key. Photographs or illustrations of many genera and most species do not exist in the published literature, making confident identifications difficult. Further adding to the difficulty of identifications is the large number of species descriptions based on a single specimen. For example, 83 of Champion’s 194 (42.7%) species described in the "Biologia Centrali-Americana" (1906b, 1909, 1910a) that are currently considered to be conoderines are single-specimen descriptions that are reliant on color pattern, often resulting in several similar species equally agreeing with descriptions.

The impetus for this study came from the difficulty in circumscribing taxonomic projects on the Conoderinae due to the large amount of undescribed species that do not fit into generic concepts as currently formed. As such, the intention of this paper is to summarize current knowledge and provide basic phenotypic information for the genera of Conoderinae found in North and Central America and the Caribbean, thus providing a *status quo* of classificatory, systematic and biological knowledge on the Conoderinae, and as a result providing a foundation to facilitate future taxonomic studies and the identification of specimens. While many of the genera are currently suspect in their tribal placements and many of the presently recognized genera are likely to not represent monophyletic groups, only four changes in the placement of genera are proposed here due to the current lack of phylogenetic evidence, limited observances of relevant type material and unexamined potentially related South American taxa. Those four transfers are justified because they have not been hypothesized to be related to other genera not observed in this study, and furthermore, their new tribal placement strengthens the hypothesis of monophyly both of the tribe they were transferred from and their new tribal placement. Evidence is presented for alternative placements for other genera but further classificatory changes are reserved for an ongoing phylogenetic analysis.

The sequence of this review is as follows: I provide an overview of the diversity, distribution, natural history, and behavior of Conoderinae; a summary of the classificatory history and current morphological circumscription of Conoderinae; a survey of several morphological character systems; an identification key to genera occuring in the focal region; a systematic review section treating each tribe and genus in detail; and suggestions for future studies on the Conoderinae.

## Materials and methods

Specimens were observed with a Leica M125 stereomicroscope. Habitus images were taken with a Visionary Digital Passport II system using a Canon EOS 5D Mark II camera, slices were stacked in Zerene Stacker version 1.04 and edited in Adobe Photoshop CS6 version 13.0.1 to produce a uniform background. Images of morphological structures were taken with a BK Lab imaging system with a Canon EOS 7D camera equipped with either a 100 mm macro lens or an Infinity K2 long distance microscope with a 5x objective and stacked in Zerene Stacker, or images were taken with a Leica DFC 450 camera attached to a M205 C stereomicroscope and stacked using the Leica Application Suite (LAS) version 4.1.0. Figure 19 was taken with a JEOL JSM 6300 Scanning Electron Microscope.

Specimens from the following collections were observed in this study:

**ASUHIC** Arizona State University Hasbrouck Insect Collection, Tempe, AZ, USA

**CMNC** Canadian Museum of Nature Collection, Ottawa, Canada

**CWOB** Charles W. O’Brien Collection, Green Valley, AZ, USA

**MIUP** Museo de Invertebrados G.B. Fairchild, Universidad de Panamá, Panamá

**NMNH** National Museum of Natural History, Washington DC, USA

**PCMENT** Programa Centroamericano de Maestria en Entomología, Universidad de Panamá, Panamá

**SSAC** Salvatore S. Anzaldo Collection, Tempe, AZ, USA

**STRI** Smithsonian Tropical Research Institute, Balboa, Ancón, Panamá

### Diversity and distribution

At present, Conoderinae is organized into 14 tribes and 209 genera worldwide (following Alonso-Zarazaga and Lyal 1999, Bouchard et al. 2011, and incorporating changes and additions made in Kojima and Lyal 2002, Davis and Engel 2006, and Riedel et al. 2016), and over 2,000 species (Prena et al. 2014), placing it among the largest of the weevil subfamilies. Only five of those tribes and 62 genera are represented in the New World and all five tribes and 40 of the genera occur in the focal region north of South America. An additional 25 genera are placed in the largest three tribes, 22 of which occur exclusively in South America. Little or no material has been observed of many of the South American genera and their treatment is reserved for future study.

Like most groups of weevils, Conoderinae contains numerous undescribed species and genera – Hespenheide (2005b, 2007) reported over 100 undescribed species of the genus *Eulechriops* Faust, 1896 from a single locality in Costa Rica. The most speciose of the herein treated genera, in numbers of described species from North and Central America only, are *Eulechriops* (57), *Macrocopturus* Heller, 1895 (54), *Lechriops* Schoenherr, 1825 (49), and *Cylindrocopturus* Heller, 1895 (41), all of which are in need of revision and likely do not represent monophyletic groups as currently constructed. *Cratosomus* Schoenherr, 1825 (25), *Piazurus* Schoenherr, 1825 (19), and *Zygops* Schoenherr, 1825 (18) are also large genera that are much more diverse in South America. Of those largest genera, only *Piazurus* (in Fiedler 1936) and *Cratosomus* (in Emden 1933) have been subject to taxonomic scrutiny since their original description. Including South American diversity, the only New World genera with over 100 described species are *Cratosomus* and *Macrocopturus* (Prena et al. 2014). Many of the larger genera can only be identified by negative identification of the likely closely related, less speciose and better circumscribed genera, highlighting the need for a detailed analysis of morphological character systems and a revised classification. Eight genera remain monotypic although undescribed species are known from many of them.

No known genera of conoderines are endemic to the Caribbean region and relatively few species are recorded from there, mainly from Guadeloupe (Hustache 1932a) and Cuba (Zayas 1988). The six Cuban species described by Zayas (1988) have been recorded here in the genera they were originally described in, although from photographs and the descriptions it is evident that some may belong in a different genus. Since the specimens were not examined their generic placement could not be confirmed and thus no changes are made to their placement here.

In addition to the extant genera, five species of the extinct genus *Geratozygops* Davis & Engel, 2006 have been described from Dominican and Mexican ambers (Zimmerman 1971, Davis and Engel 2006, Poinar and Legalov 2013). Only one other species of fossil conoderine has been described, placed in the genus *Eulechriops* (Poinar and Legalov 2013).

*Natural history and behavior.* Conoderinae are more diverse and abundant in tropical regions, especially at middle-elevation wet forests (Hespenheide 1995). Most conoderines are distinctive from other weevils for their “very active and squirrel-like” behavior (Champion 1906b: 1), being alert and quick to fly when faced with a threat (Lyal 1986). They can be found most commonly in two microhabitats (Hespenheide 1995): on the underside of foliage and on upright or fallen tree trunks, where they often perch motionlessly. A three-year light-trapping study conducted in six localities in Panama (Wolda et al. 1998) yielded 234 different species currently classified as Conoderinae, of which only 51 (21.7%) could be identified to a described species. Most species are thought to be diurnal (Hespenheide 1995) – only 17 of the 234 species collected by Wolda et al. (1998) were represented by more than 10 specimens.

Conoderines are thought to be mainly wood or stem boring as larvae (Hespenheide 1980: 331, R.S. Anderson 1993: 218, Prena et al. 2014), but host associations for the group are very poorly known. The largest contributions to the knowledge of conoderine host associations are from rearing surveys of specific plant groups – LaPierre (2002) reared 27 species of Conoderinae in 7 genera from stems and petioles of various Urticaceae Juss. and Fassbender (2013) reared 13 species of Conoderinae in 4 genera from dead branches of Lecythidaceae A. Rich. Costa-Lima (1956) summarized known host information for Brazilian species.

Immature stages are known only from a few species of agricultural importance. A contributing factor to this lack of knowledge of larvae and pupae is likely that the majority of specimens are collected with either passive collecting techniques (e.g. malaise traps) or by hand while they perch on tree trunks – neither method results in the recording of a host plant, since the tree perched on is often used by multiple genera (Hespenheide 1995) and is not thought the be the host plant.

A few genera with known host associations are very specialized and are rarely found away from their host plant (e.g. *Lissoderes* Champion, 1906 and *Pseudolechriops* Champion, 1906 on *Cecropia* Loefl. leaves). Many genera remain monotypic (e.g. *Euzurus* Champion, 1906 and *Poecilogaster* Heller, 1895) and are relatively rare in collections – the host plants of these genera remain unknown, and it is possible that they are not actually rare as was the case for *Lissoderes* and *Pseudolechriops*, which were initially described as monotypic from very few specimens and now have multiple described species that are easily collected in a specific microhabitat but rarely found elsewhere in the environment (Hespenheide 1987, LaPierre 2002, Hespenheide and LaPierre 2006).

### Review of classificatory history

The first treatment of genera now included in Conoderinae was by Schoenherr (1825, 1826) who included *Cratosomus*, *Zygops* (including the subgenera *Copturus* Schoenherr, 1825, *Piazurus*, and *Coryssopus* Schoenherr, 1826), *Mecopus* Schoenherr, 1825, *Lechriops*, and *Pinarus* Schoenherr, 1826 under “Divisio 3. Cryptorhynchides” for having a curved rostrum and a more-or-less distinctly deep rostral channel. This classification was refined by Schoenherr (1837, 1838), where the Cryptorhynchides were split into two “*Cohortes*”, I (1837: 1; including *Cratosomus* and *Lechriops*) which has the prosternum distinctly canaliculate, continuous on the mesoventrite and distinctly terminated, and II (1837: 360, which included *Zygops*, *Copturus*, *Piazurus*, *Timorus* Schoenherr, 1838, *Pinarus*, and *Mecopus* in 1838) containing those with a less distinctly canaliculate prosternum that is usually not continuous on the mesoventrite and never distinctly terminated. This classification was largely unchanged in the subsequent influential work by Schoenherr (1845), which saw the addition of numerous species, several of which became type species of subsequently described genera but only two more New World conoderine genera (*Lobops* Schoenherr, 1845 and *Peltophorus* Schoenherr, 1845).

Lacordaire’s (1865) classification represents the first grouping of genera into the three largest New World tribes recognized today mainly based on sternal modification to receive the rostrum in repose. He divided the New World representatives of “Tribu *Zygopides*” – those having both a broad metanepisternum that extends between the metacoxae and the elytra, large eyes, a canaliculate prosternum, and an antennal funicle of seven articles (as summarized by Pascoe 1871: 199) – into three groups: the “*Piazurides*”, the “*Lechriopides*” and the “*Zygopides vrais*”. Pascoe (1871: 198) noted the inadequacy of this system but provided no alternative classification.

Lacordaire’s classification was amended only slightly by K.M. Heller (1894) to accommodate the thirty-three mainly Old World genera described by Pascoe since Lacordaire’s work as well as the two genera and many species newly described by Heller therein. In Heller’s key (1984: 3) the New World Conoderinae are characterized by an antennal funicle composed of seven articles and the presence of a rostral channel at least on the prosternum, but are not further separated morphologically from several Old World genera that share those characters. Heller (1895) further amended this to accommodate seventeen new New World genera, and provided an identification key based largely on eye shape, the modification to the mesoventrite and the relative lengths of the antennal funicular articles.

Champion’s (1906b) “Zygopina” section of the *Biologia Centrali-Americana* represents the most recent major taxonomic treatment of Central American Conoderinae, in which he authored 14 genera and 194 species (also in 1909, 1910a) pertaining to the Conoderinae as currently recognized. Champion noted that his arrangement of the genera would have been presented in the order given by Heller (1895) “...were it not more convenient, to avoid delay in publication, to deal with the genera seriatim, irrespective of their relationships...” (Champion 1906b: 1). Despite this, the order the genera are presented by Champion do seem to have been done so with consideration of potential relationships, and the next catalog of Conoderinae, Hustache’s pars 134 of the Coleopterorum Catalogus (1934), presented a classification in nearly the exact order arranged by Champion, with genera treated on Champion’s pages 2–21 representing the Piazurini Lacordaire, 1865, 21–87 representing the Zygopini Lacordaire, 1865, and 87–130 the Lechriopini Lacordaire, 1865, with the single exception of *Euzurus* on page 45 placed in the Lechriopini. Many of those genera were grouped in Lacordaire’s tribes without bearing the characters originally indicated, and no updated tribal diagnoses have been presented.

This classification scheme remained almost completely unchanged, despite a foreshadowing of its probable inadequacy in reflecting the evolutionary history of numerous lineages of Conoderinae by Böving (1926) and Hustache (1938), until several genera of Zygopini were transferred to the Lechriopini by Lyal et al. (2006). Böving’s (1926) comparative study of larvae and pupae (representing the only comparative study of conoderine immatures) of the genera *Peltophorus*, *Cylindrocopturus*, and *Eulechriops*, all of which at the time were included in the Zygopini, revealed *Cylindrocopturus* and *Eulechriops* to share multiple larval and pupal characters, suggesting a closer relationship to each other than either is to *Peltophorus*. Böving later (1927) created the tribe Cylindrocopturini to include both *Cylindrocopturus* and *Eulechriops*. The current classification includes *Eulechriops* in the Lechriopini and *Cylindrocopturus* and *Peltophorus* in the Zygopini due to the presence of modification to the mesoventrite in *Eulechriops* in the form of a carinate channel and an unmodified or only slightly modified mesoventrite (not a carinate channel) in *Cylindrocopturus* and *Peltophorus*. *Cylindrocopturus* was included in the Lechriopini by Kissinger (1964) and Hatch (1971) but in the Zygopini in all subsequent works – the genus was not among the lechriopines moved by Lyal et al. (2006) due to both an unmodified mesoventrite and lack of sclerolepidia (modified scales along the metanepisternal suture), while other genera that are possibly related to *Cylindrocopturus* that have sclerolepidia (e.g. *Macrocopturus*) were moved. It seems likely that after further examination many of the genera currently in the Zygopini will be shown to be more closely related to lechriopines than to *Zygops*, *Peltophorus*, and a few additional South American genera (e.g. *Parazygops* Desbrochers, 1890, *Colpothorax* Desbrochers, 1890) that make up the “true zygopines”, as Böving’s work implied (see treatment of Zygopini below).

Hustache (1938: 58) noted the interesting distribution of “granules” on the metathoracic episterna (i.e. sclerolepidia) and suggested that a further study of these structures may provide an updated classification from that of Lacordaire. The classification used in this paper follows Alonso-Zarazaga and Lyal (1999), accommodating the changes made in Lyal et al. (2006), where twelve genera (eight from the focal region) were moved from Zygopini to Lechriopini due to the presence of sclerolepidia and/or a modified mesoventrite.

*Current circumscription of the New World Conoderinae.* Presently, most of the species of Conoderinae can be recognized by the following combination of characters, agreeing with Lacordaire (1865) and Heller (1894): large eyes that take up much of the surface of the head, a rostral channel at least on the prosternum to receive the rostrum in repose, and an antennal funicle composed of seven articles (excepting *Philinna* Champion, 1906 and *Philides* Champion, 1906 which have six). See Table 1 for a summary of the classification used in this paper, including all genera currently placed in tribes that have representation in the focal region.

**Table 1.** Summary classification adopted in the present paper, modified from Alonso-Zarazaga and Lyal (1999), Kojima and Lyal 2002, Lyal et al. 2006 and Bouchard et al. 2011. An asterisk (*) next to a generic name indicates it is known only from South America, a circumflex accent (^) indicates an Old World distribution, and a dagger (†) indicates an extinct taxon.

Tribe	Genus
*Trichodocerini* Champion, 1906a: 713	*Trichodocerus* Chevrolat, 1879: XCII
*Piazurini* Lacordaire, 1865: 144	*Costolatychus Heller, 1906: 35
	*Cratosomus* Schoenherr, 1825: c.585
	^*Guiomatus* Faust, 1899: 100
	*Hedycera Pascoe, 1870: 457, *new placement* from Lechriopini
	*Latychellus Hustache, 1938: 59
	*Latychus Pascoe, 1872: 486
	*Lobops* Schoenherr, 1845: 116
	*Piazolechriops Heller, 1906: 44
	*Piazurus* Schoenherr, 1825: c.586
	*Pinarus Schoenherr, 1826: 307
	*Pseudopiazurus* Heller, 1906: 32
	*Pseudopinarus* Heller, 1906: 33
*Othippiini* Morimoto, 1962: 47	^*Abrimoides* Kojima & Lyal, 2002: 168
	*Acoptus* LeConte, 1876: 264, *new placement* from Lechriopini
	^*Brimoda* Pascoe, 1871: 219
	^*Brimoides* Kojima & Lyal, 2002: 163
	^*Chelothippia* Marshall, 1938: 173
	^*Egiona* Pascoe, 1874: 51
	^*Othippia* Pascoe, 1874: 49
	^*Rimboda* Heller, 1925: 238
*Lechriopini* Lacordaire, 1865: 149	*Balaninurus Heller, 1895: 51
	*Copturomimus* Heller, 1895: 63
	*Copturomorpha* Champion, 1906b: 65
	*Copturus* Schoenherr, 1825: c.586
	*Coturpus* R.S. Anderson, 1994: 480
	*Crassocopturus Rheinheimer, 2011: 71
	*Cylindrocopturinus* Sleeper, 1963: 218
	*Damurus Heller, 1895: 55
	*Eulechriops* Faust, 1896: 91
	*Euzurus* Champion, 1906b: 45
	*Hoplocopturus* Heller, 1895: 50
	*Lechriops* Schoenherr, 1825: c.586
	*Machaerocnemis Heller, 1895: 60
	*Macrocopturus* Heller, 1895: 19
	*Macrolechriops* Champion, 1906b: 126
	*Microzurus* Heller, 1895: 13
	*Microzygops* Champion, 1906b: 46
	*Mnemyne Pascoe, 1880: 179
	*Mnemynurus* Heller, 1895: 54
	*Paramnemyne* Heller, 1895: 10
	*Paramnemynellus Hustache, 1932b: 207
*Lechriopini* Lacordaire, 1865: 149	*Poecilogaster* Heller, 1895: 16
	*Pseudolechriops* Champion, 1906b: 90
	*Psomus* Casey, 1892: 458
	*Rhinolechriops Hustache, 1939: 162
	*Tachylechriops Heller, 1895: 15
	*Turcopus* R.S. Anderson, 1994: 475
*Zygopini* Lacordaire, 1865: 150	*Acopturus Heller, 1895: 61
	*Arachnomorpha* Champion, 1906b: 47
	*Archocopturus* Heller, 1895: 56
	*Colpothorax Desbrochers, 1890: CXXIX
	*Copturosomus Heller, 1895: 61
	*Cylindrocopturus* Heller, 1895: 56
	†*Geratozygops* Davis and Engel, 2006: 255
	*Helleriella* Champion, 1906b: 32
	*Hemicolpus Heller, 1895: 57
	*Hypoplagius Desbrochers, 1891: 40
	^*Isocopturus* Hustache, 1931: 23
	*Larides* Champion, 1906b: 34
	*Lissoderes* Champion, 1906b: 47
	*Macrotimorus Heller, 1895: 59
	*Parazygops Desbrochers, 1890: CXXIX
	*Peltophorus* Schoenherr, 1845: 451
	*Phileas* Champion, 1906b: 34
	*Philenis* Champion, 1906b: 43
	*Timorus Schoenherr, 1838: 680
	^*Xeniella* Hustache, 1931: 24
	*Zygops* Schoenherr, 1825: c.586
	*Zygopsella* Champion, 1906b: 42
Conoderinae *incertae sedis*	*Philides* Champion, 1906b: 129, *new placement* from Lechriopini
	*Philinna* Champion, 1906b: 128, *new placement* from Lechriopini

The South American genera *Timorus* and *Hypoplagius* Desbrochers, 1891 each have Mexican records of otherwise South American species. Champion (1906b: 33) and subsequent authors have doubted the validity of the Mexican record for the Brazilian *Timorus suturalis* Rosenschoeld, 1838, and since no material was observed it is also here not considered to be represented in Central America. *Hypoplagius pectoralis* Desbrochers, 1891 is recorded from Brazil, French Guiana, and Veracruz, Mexico (Champion 1906b: 32 mentions three specimens from the Sallé collection). Very little material of *Hypoplagius* has been observed in the course of this study, all of it being from South America. As such, the unusual distribution of *Hypoplagius* is also suspect and the genus is not treated in detail in the present publication, but the issue requires further study.

In addition to the South American genera, three genera with Old World distributions are currently placed in the largely New World Piazurini and Zygopini: *Guiomatus* Faust, 1899 (Piazurini, from New Guinea), *Isocopturus* Hustache, 1931 (Zygopini, from Cameroon), and *Xeniella* Hustache, 1931 (Zygopini, from Tanzania). No material was observed of those genera so their placement cannot be commented on.

Two groups previously included in the Conoderinae but most recently being treated in another subfamily are the Tachygonina Lacordaire, 1865 (currently in the Curculioninae: Rhamphini Rafinesque, 1815), and the genus *Isotrachelus* Faust, 1896 (currently in the Molytinae Schoenherr, 1823: Cleogonini Gistel, 1856); see the generic treatments for *Philides* and *Psomus* Casey, 1892, respectively, for more information. For the most recent treatments of Old World tribes, see Kojima and Lyal (2002) for Othippiini Morimoto, 1962, Marshall (1959) for Campyloscelini Schoenherr, 1845, and Marshall (1939) for Coryssomerini Thomson, 1859.

### Systematic utility of select morphological character systems

Due to the lack of phylogenetic evidence supporting the current classification, it was deemed necessary to evaluate character systems that have both had influence on the present classification of Conoderinae and other character systems that are traditionally used in other groups of weevils, including: the modification to the mesoventrite for receiving the rostrum in repose, the tibial apex, and the structure of the abdomen. These character systems potentially have deeper-level phylogenetic signal and thus can be informative for a revised classification of Conoderinae. Male genitalia also appear promising for providing structure to the mid-level classification. However, they are not comprehensively analyzed here, with the focus of this review being on reliable diagnosis of conoderine genera by external characters as well as with several of the genera remaining undissected due to limited material observed in collections. See the “Systematic Review of Genera” below for a more detailed account of the variation and exceptions of these characters found in each tribe and genus. The following additional character systems that are mostly only useful for diagnosing individual species or subgeneric species groups are also reviewed: eye size and shape, modification to the metaventrite, and mimicry complexes. Morphological terminology for thoracic sclerites was adopted from Oberprieler et al. (2014).

**Mesoventrite (Figs 1–18).** The character of historical importance for the identification of conoderine tribes and genera is the modification of the mesoventrite for the reception of the rostrum in repose. This remains one of the most influential characters for a genus-level identification; it is thus of paramount importance for future taxonomic work on the Conoderinae to identify the variation in this structure and assess its validity as a character system of significance in the delimitation of tribes and genera.

**Figures 1–9.**
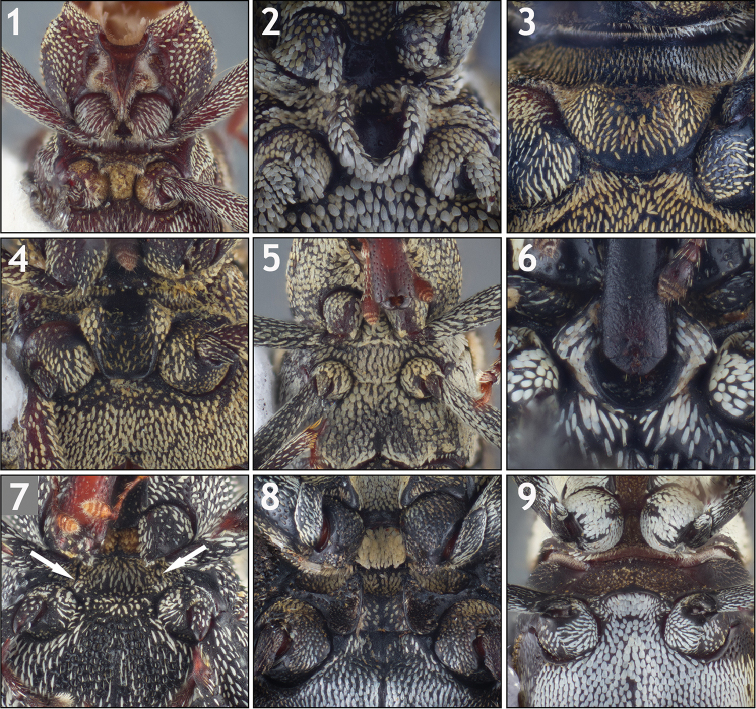
Variation in the mesoventrite. **1** *Trichodocerus brevilineatus* [ARTSYS0000616] showing a prosternal rostral “sheath” with the sides converging below the procoxae, the transverse ridge anterior to the mesocoxae and the region of the mesoventrite posterior to the ridge with dense yellow scales **2** *Lobops bonvouloiri* [ARTSYS0000527] showing a cup-shaped receptacle for receiving the rostrum **3** *Piazurus trifoveatus* [SSAC0001118] with an “open” channel on the mesoventrite **4** *Pseudopinarus condyliatus* [SSAC0001116] with an “open” channel on the mesoventrite **5** *Acoptus suturalis* [ASUHIC0016914] showing a flat, unmodified mesoventrite **6** *Copturus sanguinicollis* [ASUHIC0086638] showing a closed receptacle on the mesoventrite with lateral flanges **7** *Cylindrocopturinus pictus* [SSAC0001288] showing a rostral channel of the mesoventrite formed by relatively parallel carinae and no posterior termination **8** *Euzurus ornativentris* [ARTSYS0000796] **9** *Hoplocopturus javeti* [SSAC0001289] with an inverted U-shaped carina and the region posterior to the carina invaginated.

**Figures 10–18.**
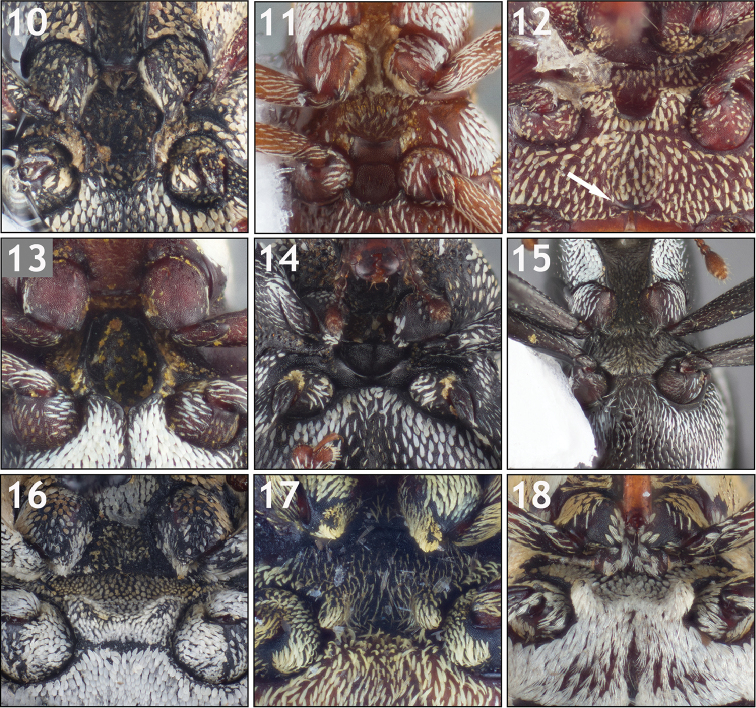
Variation in the mesoventrite. **10** *Lechriops californicus* [ASUHIC0024146] showing relatively parallel carinae marking the lateral margin of the rostral channel and a closure of the channel on the metaventrite **11** *Microzygops nigrofasciatus* [ARTSYS0000797] with tubercles anterior to the mesocoxae and slight, arcuate carinae on the anterior region of the mesoventrite (encircling the central scaled region) **12** *Paramnemyne decemcostata* [ARTSYS0000798] showing tuberculate posterolateral margins of the mesoventrite and a transverse carina near the posterior margin of the metaventrite **13** *Pseudolechriops klopferi* [SSAC0001060] showing a deep ovoid receptacle on the meso- and metaventrite **14** *Turcopus viscivorus* [ARTSYS0000530] showing a receptacle on the mesoventrite with prominently carinate posterior margin **15** *Lissoderes subnudus* [SSAC0001066] showing a completely unmodified mesoventrite covered with multifid setae **16** *Peltophorus adustus* [ASUHIC0031514] showing a ventrally expanded posterior margin of the mesoventrite to receive the rostrum in repose **17** *Philenis fuscofemorata* [ARTSYS0000659] with tubercles at the posterolateral margins of the mesoventrite and a deep depression at the posterior margin in between **18** *Philinna bicristata* [ARTSYS0000799] showing slight lamellate processes at the posterolateral margins of the mesoventrite as well as transversely flattened projections behind the procoxae.

The tendency of these weevils to fold their legs and tuck the rostrum into the rostral channel with the antenna folded underneath when dying greatly obscures the view of the ventral surface. To expose the mesoventrite, the legs can be gently moved out of the way with an insect pin, and if the rostrum also needs to be moved, the specimen can be relaxed in warm water for several minutes and the head then gently tilted upwards with a pin (while bracing the prothorax) to expose the antennae and rostral channel.

The following types of modification roughly correspond to Lacordaire’s original tribal designations, while taking into account the numerous genera described since and potentially improperly placed genera. For further discussion of variation in the mesoventrite see the tribal and individual generic accounts and the Discussion.

The rostral channel is variously referred to in the literature and in the present paper as “closed” or “open”. A “closed” rostral channel refers to the posterior margin of the channel, where the apex of the rostrum would fit in repose, being demarcated with a transverse, raised portion (e.g. Fig. 2). An “open” rostral channel refers to a rostral channel with some sort of longitudinal modification, usually in the form of raised, parallel carinae, that are lateral to the rostrum in repose but do not posteriorly demarcate the apex of the rostrum (e.g. Figs 3–4). The rostral channel in weevils can be closed on the prosternum, the meso- or metaventrite or on the abdominal ventrites or open on any of those structures. In the New World Conoderinae, the rostral channel is always present at least on the prosternum in the region anterior to the procoxae and this prosternal part of the rostral channel is open.

*Trichodocerine type* (Fig. 1). The mesoventrite has a transverse, ventrally produced ridge anterior to the mesocoxae and flattened, yellow scales in the intercoxal process posterior to the ridge. The rostral channel does not extend beyond the prosternum, which in most species can be interpreted as narrowly open. However, since the apex of the channel does not correspond with the apex of the rostrum in repose in any of the species, it is considered to not be truly “closed” on the prosternum even when a distinct termination of the channel is present. This type of modification is only found in the monogeneric tribe Trichodocerini Champion, 1906.

*Piazurine type* (Figs 2–4, 6, 8, 12). In its typical form, the modification to the mesoventrite in piazurines is open posteriorly (“gutter-like”), allowing the rostrum to extend beyond the mesoventrite to the metaventrite. The posterior margin of the mesoventrite is rounded and flattened, with the lateral portions raised (Figs 3, 4), and often overlapping the anterior border of the metaventrite. Rarely is the channel closed on the mesoventrite, with posterior margin raised to the same level as the lateral margins, forming a “cup-shaped receptacle” similar to the Cryptorhynchinae Schoenherr, 1825 (Fig. 2). The region of the mesoventrite anterior to this is never with modification (i.e. without carina, depression, etc.). This type of mesoventrite is found in all genera treated as Piazurini, the lechriopine genera *Paramnemyne* Heller, 1895 (Fig. 12) *Euzurus* (Fig. 8), *Copturus* (Fig. 6), and *Microzurus* Heller, 1895, which have more similarities with the piazurine type than the lechriopine type although they differ from the typical piazurine form.

*Lechriopine type* (Figs 7, 9–11, 13–14). The mesoventrite is variously carinate, most typically as a channel with roughly parallel or somewhat arcuate longitudinal carinae delimiting the side of the channel (e.g. Figs 7, 10). The rostral channel can be closed (e.g. Fig. 14) or open (e.g. Fig. 10) on the mesoventrite; if open on the mesoventrite the channel on the metaventrite can similarly be closed (e.g. Fig. 11) or without a distinct termination to receive the apex of the rostum (e.g. Fig. 9). The channel can be very shallow or deep, with the lateral margins slightly carinate or strongly ventrally produced. The lateral carina in some are strongly arcuate and anteriorly fused, forming an inverted U-shaped carina that does not seem to function for rostral reception (Fig. 9) and is depressed or invaginated posterior to the carina. If the posterior margin is tuberculate as in some lechriopines (e.g. Fig. 11), there are always anterior carinae. This type of mesoventrite is found in all genera included here in the Lechriopini, excepting *Paramnemyne*, *Copturus*, *Microzurus*, and *Psomus*.

*Zygopine type* (Figs 5, 15–18). The mesoventrite is unmodified (Figs 5, 15), or if with some modification, the modification is not in the form of a channel to receive the rostrum. This type of mesoventrite is found in Zygopini, in the lechriopine *Psomus*, the othippiine *Acoptus* LeConte, 1876, *Philides* and *Philinna*. Exceptions can be found in most species of *Philenis* Champion, 1906, which have ventrally produced tubercles at the posterolateral margins of the mesoventrite and a posteromedial depression (Fig. 17; but the mesoventrite of the type species is unmodified) and in *Peltophorus*, which has a ventrally produced posterior margin of the mesoventrite (Fig. 16).

**Figures 19–36.**
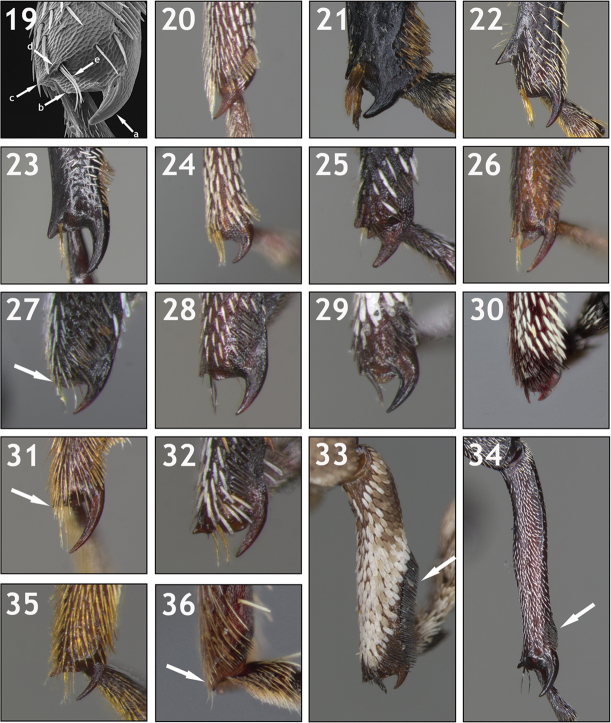
Variation in the metatibial apex. **19** Mesotibial apex of *Lechriops vestitus* illustrating parts of the tibial apex: a) posterior apical angle with large, curved uncus; b) middle of the apex with produced, rounded inner flange; c) anterior apical margin with premucro; d) oblique ridge of premucro; e) apical setal tuft **20** Male *Trichodocerus brevilineatus* [ARTSYS0000609] with a tibial uncus arising at the middle of the apex **21** *Cratosomus punctulatus mexicanus* [ASUHIC0031510] with thick fascicles of golden setae near the anterior apical margin and a dense setal brush at the posterior apical face **22** *Pseudopiazurus centraliamericanus* [ASUHIC0086627] with a large, subapical premucro **23** *Piazurus laetus* [SSAC0001077], showing a typical tibial apex for that genus **24** *Acoptus suturalis* [ASUHIC0016915] **25** *Cylindrocopturinus pictus* [SSAC0001288] with a pointed, premucro-like inner flange **26** *Pseudolechriops klopferi* [SSAC0001060] showing an elongate, uncus-like inner flange. **27** *Arachnomorpha circumlineata* [ARTSYS0000535] **28** *Archocopturus medeterae* [ASUHIC16884] **29** *Cylindrocopturus adspersus* [ASUHIC0016896] with a rounded, produced inner flange **30** *Helleriella longicollis* [ASUHIC0065241] with a very short uncus **31** *Lissoderes cecropiae* [ASUHIC0064707] with an elongate uncus and minute premucro **32** *Phileas granulatus* [ARTSYS0000528] with the middle of the apex (between uncus and premucro) sunken **33** *Peltophorus adustus* [ASUHIC0012325] with a short uncus and posterodistal setal comb extending halfway to the base of the tibia **34** *Zygops erythropygus* [ASUHIC0086640] with short posterodistal setal comb. **35** *Philenis flavipes* [ASUHIC0065102] **36** *Philinna bicristata* [ARTSYS0000799] with a small tooth at the anterior apical angle.

**Tibial apex (Figs 19–36).** The tibial apex of Conoderinae is interpreted for the majority of genera and species, largely following the morphological terminology of Thompson (1992), as bearing a large uncus at the posterior apical angle, a premucro at the anterior apical angle and a variously produced inner flange at the apex between them. The terminology of “anterior” and “posterior” is adopted here instead of the frequently used “inner” and “outer”, respectively, to avoid confusion when referencing the “mesal” and “lateral” faces of the femoral apex (which are synonymous with “inner” and “outer”, respectively). The adopted terminology is in reference to the position of the structures of the tibial and femoral apex of the hind leg in its life-like postion (as in the left hind leg of Fig. 101a). The apex usually bears two setal tufts, which can vary from one to a few setae (Fig. 23) to a thick fascicle of setae (Fig. 21), at the anterior apical angle arising from oblique carinae that are part of the premucro. See Figure 19 for a clarification of terminology.

While there can be slight differences between the pro-, meso- and metatibial apices (e.g. the protibial uncus is often larger than the meso- and metatibial uncus), unless otherwise specified the one discussed and figured is the metatibia of the left leg. The shape and size of the uncus is quite variable but most commonly long and slightly curved (e.g. Figs 23, 28, and 34 for typical form) but varies from being more elongate and thin (Fig. 31), hooked (Fig. 29) and very short (Figs 30, 33). The size, position, and orientation of the premucro varies as well, from being large, subapical and oriented at a 45° angle to the longitudinal axis of the tibia (Fig. 22), small, apical, and oriented at 45° angle to the longitudinal axis of the tibia (Figs 32, 35), small, apical, and oriented with the longitudinal axis of the tibia (Figs 28, 29), apical and minute (Figs 27, 31), or absent (Fig. 24). Many genera also have a third apical prominence at the middle of the apex between the uncus and premucro, which here is interpreted as a modification of the inner flange of Thompson (1992). This inner flange varies from being a simple carina, not produced ventrally (Fig. 23), slightly produced ventrally and rounded (Fig. 28), strongly produced ventrally and rounded (Fig. 29), small, pointed and premucro-like (Fig. 25), and elongate, pointed, and uncus-like (Fig. 26). While a few genera can be diagnosed by the structure of the tibial apex alone, many of the modifications, especially those of the inner flange, appear to be homoplasious as they can be found in species of several unrelated genera.

**Abdominal sclerites.** The structure of the abdominal ventrites and tergites are potentially of significance at the tribal level with the apex of the abdomen being opened in different ways, with either an exposed or concealed pygidium. When exposed, the pygidium can be visible in dorsal view (Figs 99b, 102b) or only in posterior or ventral view (e.g. Fig. 68a), and abdominal ventrites are flat or at most slightly evenly ascending. When concealed, abdominal tergites can be slightly (Fig. 73a) to very strongly (Fig. 76a) ascending with the last three tergites forming a ventral pygidium-like hinge to open the apex of the abdomen. Very few species exhibit sexual dimorphism in the exposure of the pygidium.

**Figures 37–45.**
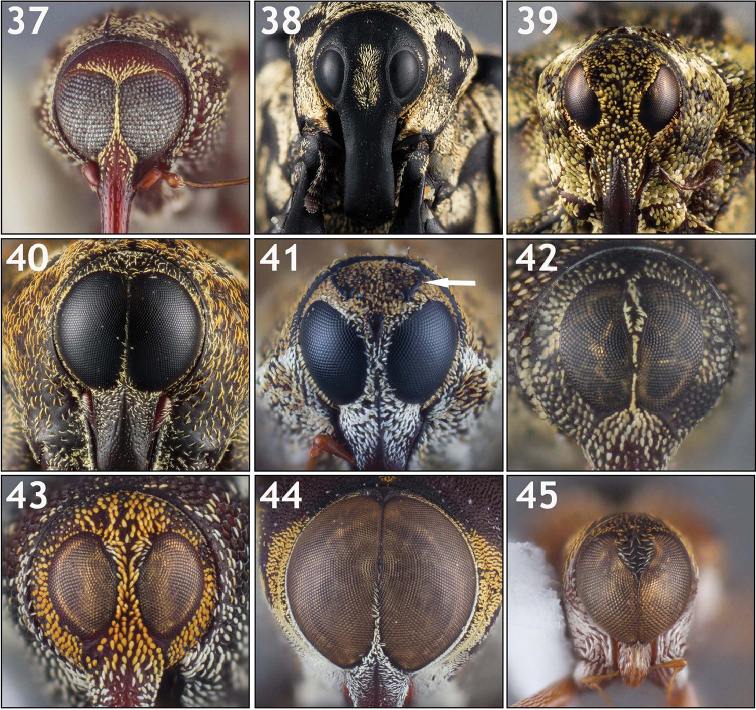
Variation in eye shape. **37** *Trichodocerus brevilineatus* [ARTSYS0000609] with large, subcontiguous eyes that continue below rostral insertion **38** *Cratosomus punctulatus mexicanus* [ASUHIC0031510] with relatively small, widely separated eyes **39** *Lobops bonvouloiri* [ARTSYS0000658] with widely separated eyes and the frons concave between the upper half of the eye **40** *Pseudopiazurus centraliamericanus* [ASUHIC0086627] with very large eyes that are contiguous in upper half **41** *Pseudopinarus guyanensis* [ASUHIC0086636] with large, subcontiguous eyes and an arcuate carina on the vertex of the head **42** *Acoptus suturalis* [ASUHIC0016914] with subcontiguous eyes separated at the top by a lanceolate space **43** *Cylindrocopturinus pictus* [SSAC0001288]**44** *Macrocopturus lynceus* [SSAC0001085] with very large, partially contiguous eyes **45** *Microzygops nigrofasciatus* [ARTSYS0000802] with large eyes contiguous in the bottom 2/3 and widely separated at the top.

**Figures 46–54.**
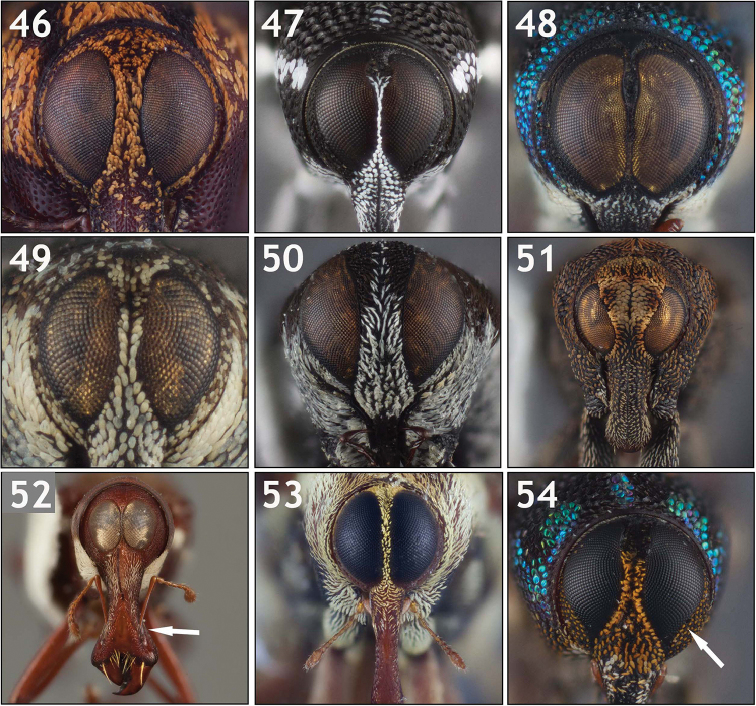
Variation in eye shape. **46** *Poecilogaster brevis* [ARTSYS0000805] with relatively vertical, separated eyes **47** *Arachnomorpha circumlineata* [ARTSYS0000535] showing subcontiguous eyes separated at the top by a broad interocular space **48** *Archocopturus medeterae* [ASUHIC0086637] with subcontiguous eyes separated at the top by a thin lanceolate space **49** *Cylindrocopturus quercus* [ASUHIC0016905] with vertical, separated eyes **50** *Helleriella longicollis* [ASUHIC0065241] with widely separated eyes and a very thin rostrum **51** *Larides cavifrons* [ASUHIC0016882], showing a strongly depressed interocular space **52** Male *Lissoderes cecropiae* [ASUHIC0064708] showing oval, subcontiguous eyes and an apical antennal insertion on the rostrum **53** *Philenis fuscofemorata* [ARTSYS0000659] showing ovoid eyes and slender antennae **54** *Zygopsella ruficauda* [ARTSYS0000526] with a sinuous lateral and mesal margin of the eyes which is strongly inflexed at the lower lateral margin.

**Eye size and shape (Figs 37–54).** Eye size and shape was used by Heller (1895: 3) as a major character in his key for separating groups of genera, namely the distance between the eyes and the shape of the eyes at the bottom and sides. The eyes of Conoderinae are typically large (taking up much of the surface of the head) and approximate, where they can be subcontiguous and separated by one to a few rows of scales (Figs 37, 41), or contiguous in part (as in Figs 40, 44, 45). Smaller, more widely separated eyes (Figs 38, 39, 51) are less common. Variation in the shape of the eye varies from being circular to ovoid (Figs 38, 52), acuminate at the lower margins (e.g. Fig. 49), sinuous along the lateral margin, with the lower lateral margin being inflexed (Fig. 54), with a distinct interocular space (the upper mesal margin being sinuous and inflexed) that can be lanceolate (Figs 42, 48), ovate (Figs 45, 47), or broad (Figs 39, 51). When separated, the interocular space at the top can be evenly convex with the rest of the surface of the head, slightly depressed, or concave (compare Fig. 51 with 97b).

**Metaventrite modification.** The modification to the metaventrite for receiving the rostrum is much more variable within a genus than the modification to the mesoventrite and is independent of the presence of modification to the mesoventrite. Modification can be absent (Fig. 15), but when present it is usually limited to the anterior, intermesocoxal portion of the metaventrite in the form of a depression (Fig. 8), an excavated anterior margin to receive the rostrum (Fig. 13), or a deep fovea (Fig. 11). In genera that have longer rostra, there can be a depression in the middle of the metaventrite (Fig. 9), a depression and a transverse carina marking the apex of the rostrum in repose (Fig. 12), or a longitudinal channel or depression along the entire length of the sclerite.

**Mimicry complexes.** Several putatively mimetic color patterns are hypothesized to have evolved independently in multiple genera of New World Conoderinae, including: red-eyed flies (Hespenheide 1973, 1995; Figs 80, 87), dolichopodid flies of the genus *Medetera* Fischer von Waldheim, 1819 (Hespenheide 1995, 2005; Figs 94, 103), ants of the genus *Zacryptocerus* Kempf, 1973 (Hespenheide 1986), other species of ants (Hespenheide 1995; e.g. Figs 86, 93), bees (Hespenheide 1995; Fig. 101), clytrine chrysomelids (Hespenheide 1995, 1996; Fig. 96), and additional convergent color patterns without an identified model, such as the “red-spotted” complex (Hespenheide 1995, Hespenheide 2017). The presence of these mimicry complexes can make genus- and species-level identifications difficult due to strong convergences in body shape and coloration patterns that result in species superficially resembling species in other genera more than closely related species. Characters linked to the mimicry complexes, such as color, are avoided for diagnostic use in the identification key whenever possible.

### Systematic review of genera

**Format of accounts**. Genera are grouped into tribes within subfamily Conoderinae following the classification of Alonso-Zarazaga and Lyal (1999) and Lyal et al. (2006), which largely reflects the status quo for mid-level classification of Conoderinae (see Table 1 for overview). Provided below for each genus is the type species information, synonymic history, gender, differential diagnosis, references to taxonomic treatments, geographic ranges, number of described species from the focal range, number of species known outside the focal range, host associations (if known), and at least a dorsal and lateral habitus image (see “Species Representation” section below). The higher-level entities are also briefly reviewed, providing a classificatory history, variation in the key character systems discussed above and the diversity, distribution and morphological circumscription of genera currently included. Subgenera are indicated by rounded brackets. The gender provided for all generic names follows Alonso-Zarazaga and Lyal (1999).

*Diagnosis*. Diagnoses provide characters or combinations of characters that distinguish each genus from its putative relatives, some largely following those given by Champion (1906b). Many genera, especially the largest genera, are definable only by a combination of the following three characters: the relative lengths of the first two funicular articles, the modification to the mesoventrite, and the structure of the hind femora (whether it is ventrally toothed and externally carinate). In many cases the combination of these characters is not exclusive to a genus or has exceptions within a genus, but as many of the genera are currently constructed that is the best way to separate the majority of the species. Those three characters are given for most genera regardless of their diagnostic quality for that genus.

*Keys*. Published keys treating at least some of the currently recognized species in each genus from the focal region are provided, presented in order of relevance (i.e. treating the most number of species or covering a larger geographic range). If the species in that key are treated under a different generic name than their current placement, that name is also provided.

*Species numbers and ranges.* Species counts and geographic ranges for genera are given from O’Brien and Wibmer (1982) and Alonso-Zarazaga and Lyal (1999), respectively, with updates where indicated. Species counts provided are the number of species currently recorded for only North and Central America and the Caribbean. A range including “South America” indicates additional species known from South America or the range of at least one Central American species extends into South America. The number of additional species known in South America are also provided, following Wibmer and O’Brien (1986) and including more recent additions.

*Species Representation.* The species selected for the accompanying habitus images is the type species for the genus if that species is known from the focal region and if specimens were available for study and in acceptable condition; these criteria were met for 21 of the 39 genera. If the type species is not from the focal region or the type species is from the focal region but specimens were not available or in acceptable condition, a species deemed a typical representative of the genus was used instead.

*Host associations.* All referenced names of botanical species are the accepted name from The Plant List (2013; theplantlist.org) and higher-level entities are the accepted name from Tropicos (2017; tropicos.org) at the time of access

**Specimen availability.** The species-level identifications of many observed specimens are tentative without comparison to type material, and since many species, especially mimetic ones, are found to consist of complexes of numerous undescribed but closely related sibling species (see Hespenheide 2005), photographed or otherwise referenced specimens in this paper, as often as possible, were given a unique identifier databased in the Symbiota Collections of Arthropod Network (SCAN; Gries et al. 2014). This allows future work on the Conoderinae to build off of this study by making some of the exact specimens used easily located so their identifications and morphological interpretations can be re-evaluated. Images used or specimens referenced belonging to the ASUHIC, SSAC, and STRI collections are accompanied by a unique identifier for their respective repository in SCAN (e.g. ASUHIC0016837, SSAC0001113, STRI_ENT_0123144). Specimens loaned from the other collections listed above were databased in SCAN with a unique identifier in the SCAN-ARTSYS collection, with the home institution entered in the “Owner Code” field (e.g. ARTSYS000530). See Table 2 for a list of all taxa and specimens featured in photographs.

**Table 2.** Taxon and specimen representation used in figures. Each identifier signifies the specimen used for all photos taken for that species unless otherwise specified.

Tribe	Taxon	Specimen identifier
Trichodocerini	*Trichodocerus brevilineatus* Champion, 1906	ARTSYS0000616 (Fig. 1) ARTSYS0000609 (Figs 20, 37)
	*Trichodocerus spinolae* Chevrolat, 1879	ARTSYS0000534
Piazurini	*Cratosomus lafontii* Guérin, 1844	SSAC0001133
	*Cratosomus punctulatus mexicanus* Gyllenhal, 1837	ASUHIC0031510
	*Lobops bonvouloiri* (Hustache, 1932)	ARTSYS0000658 (Fig. 39) ARTSYS0000527 (Figs 2, 69)
	*Piazurus caprimulgus* (Olivier, 1807)	SSAC0001113
	*Piazurus laetus* Pascoe, 1886	SSAC0001077
	*Piazurus trifoveatus* Champion, 1906	SSAC0001118
	*Pseudopiazurus centraliamericanus* (Heller, 1906)	SSAC0001291 (Fig. 64) ASUHIC0086627 (Figs 22, 40 71)
	*Pseudopinarus condyliatus* (Boheman, 1838)	SSAC0001116 (Fig. 4) ASUHIC0086626 (Fig. 72)
	*Pseudopinarus guyanensis* Hustache, 1938	ASUHIC0086636
Othippiini	*Acoptus suturalis* LeConte, 1876	ASUHIC0016914 (Figs 5, 73) ASUHIC0016915 (Fig. 24)
Lechriopini	*Copturomimus caeruleotinctus* Champion, 1906	SSAC0001059
	*Copturomimus cinereus* Heller, 1895	ASUHIC0086628
	*Copturomorpha* Champion, 1906 sp.	ASUHIC0086641
	*Copturus aurivillianus* (Heller, 1895)	ASUHIC0024140
	*Copturus sanguinicollis* (Champion, 1906)	ASUHIC0086638
	*Coturpus arcuatus* R.S. Anderson, 1994	ARTSYS0000531
	*Cylindrocopturinus pictus* (Schaeffer, 1908)	SSAC0001288
	*Eulechriops minutus* (LeConte, 1824)	ASUHIC0024145
	*Euzurus ornativentris* Champion, 1906	ARTSYS0000796 (Figs 8, 65) ARTSYS0000800 (Fig. 80)
	*Hoplocopturus javeti* (Champion, 1906)	SSAC0001289
	*Hoplocopturus sulphureus* Champion, 1906	ARTSYS0000801
	*Hoplocopturus varipes* Champion, 1906	SSAC0001086
	*Lechriops californicus* (LeConte, 1876)	ASUHIC0024146
	*Lechriops vestitus* (Boheman, 1838)	SSAC0001114 (Fig. 82)
	*Macrocopturus lynceus* (Champion, 1906)	SSAC0001085
	*Macrolechriops spinicoxis* Champion, 1906	ARTSYS0000529
	*Microzurus championi* Hustache, 1934	ASUHIC0031507
	*Microzurus* Heller, 1895 sp.	SSAC0001290
	*Microzygops nigrofasciatus* Champion, 1906	ARTSYS0000797 (Fig. 11) ARTSYS0000802 (Fig. 45, 86)
	*Mnemynurus poeciloderes* Champion, 1906	ARTSYS0000803
	*Paramnemyne decemcostata* Champion, 1906	ARTSYS0000798 (Fig. 12) ASUHIC0065104 (Fig. 88)
	*Poecilogaster brevis* (Waterhouse, 1879)	ARTSYS0000805 (Fig. 46) ASUHIC0086631 (Fig. 89)
	*Pseudolechriops klopferi* Hespenheide & LaPierre, 2006	SSAC0001060
	*Pseudolechriops megacephalus* Champion, 1906	ASUHIC0086629
Lechriopini	*Psomus armatus* (Dietz, 1891)	ARTSYS0000533
	*Turcopus viscivorus* R.S. Anderson, 1994	ARTSYS0000530
Zygopini	*Arachnomorpha circumlineata* Champion, 1906	ARTSYS0000535
	*Archocopturus laselvaensis* Hespenheide, 2005	ASUHIC0086633
	*Archocopturus medeterae* Hespenheide, 2005	ASUHIC0016884 (Fig. 28) ASUHIC0086637 (Fig. 48)
	*Cylindrocopturus adspersus* (LeConte, 1876)	ASUHIC0016896
	*Cylindrocopturus quercus* (Say, 1831)	ASUHIC0016905 (Fig. 49) ARTSYS0000819 (Fig. 95)
	*Helleriella longicollis* Champion, 1906	ASUHIC0065241
	*Larides cavifrons* Champion, 1906	ASUHIC0016882
	*Lissoderes cecropiae* Hespenheide, 1987	ASUHIC0064707 (Fig. 31) ASUHIC0064708 (Fig. 52)
	*Lissoderes subnudus* Champion, 1906	SSAC0001066 (Fig. 15) SSAC0001064 (Fig. 56) SSAC0001136 (Fig. 98)
	*Peltophorus adustus* (Fall, 1906)	ASUHIC0012325
	*Peltophorus polymitus seminiveus* (LeConte, 1884)	SSAC0001117
	*Peltophorus polymitus suffusus* (Casey, 1892)	ASUHIC0016837
	*Phileas granulatus* Champion, 1906	ARTSYS0000528
	*Philenis flavipes* Champion, 1906	ASUHIC0065102
	*Philenis fuscofemorata* Champion, 1906	ARTSYS0000659
	*Zygops erythropygus* Champion, 1906	ASUHIC0086640
	*Zygops vitticollis* Desbrochers, 1891	ASUHIC0086634
	*Zygopsella ruficauda* Champion, 1906	ARTSYS0000526
*Incertae sedis*	*Philides comans* Champion, 1909	ARTSYS0000804
	*Philinna bicristata* Champion, 1906	ARTSYS0000799 (Figs 18, 57) ARTSYS0000532 (Fig. 105)

## Taxonomic treatment

### Conoderinae

Taxon classificationAnimaliaColeopteraCurculionidae

Schoenherr, 1833: 26

#### Remarks.

The five tribes represented in the New World are unlikely to represent a monophyletic group and as such cannot be satisfactorily diagnosed by morphological characters or separated from the Old World tribes as a whole, even when excluding the aberrant genera.

### Trichodocerini

Taxon classificationAnimaliaColeopteraCurculionidae

Champion, 1906: 713

#### Classificatory history.

This monotypic tribe has been enigmatic in its placement in Curculionidae since the description of its sole genus by Chevrolat in 1879. *Trichodocerus* Chevrolat, 1879 was originally considered by Chevrolat to be near *Conotrachelus* Dejean, 1835 and it has since been treated as or had its species described in the Cryptorhynchinae (Champion 1906: 713, Hustache 1936, Papp 1979, O’Brien and Wibmer 1982, Wibmer and O’Brien 1986, Zherikhin and Gratshev 1995), Baridinae (Bondar 1946) and Conoderinae (Wibmer and O’Brien 1989: 15, Wolda et al. 1998, Alonso-Zarazaga and Lyal 1999, Lyal et al. 2006, Bouchard et al. 2011, Prena et al. 2014), where it currently resides.

### Trichodocerus

Taxon classificationAnimaliaColeopteraCurculionidae

Chevrolat, 1879: XCII

**Figures 67–70.**
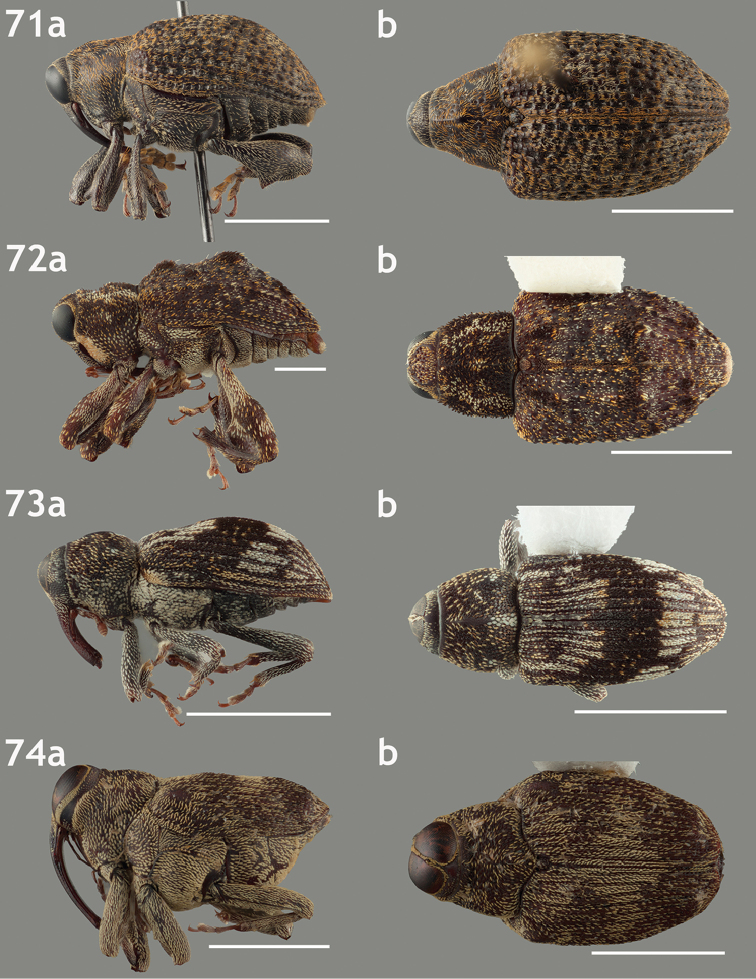
Lateral and dorsal habitus images of Trichodocerini and Piazurini. Scale bars = 2 mm unless otherwise specified. **67a–b** *Trichodocerus spinolae* [ARTSYS0000534] **68a–b** *Cratosomus lafontii* [SSAC0001133]; scale bars = 10 mm **69a–b** *Lobops bonvouloiri* [ARTSYS0000527] **70a–b** *Piazurus caprimulgus* [SSAC0001113].

Figs 1 , 20 , 37 , 67

= Mallerus Bondar, 1946: 86 [Syn.: Bondar 1947: 294]. Type species: Mallerus antiquus Bondar, 1946 [by original designation].

#### Type species.

*Trichodocerus spinolae* Chevrolat, 1879 [by subsequent designation: Champion 1906: 713].

#### Gender.

Masculine.

#### Diagnosis.

*Trichodocerus* is easily separated from all other genera treated here by the loose antennal club, the presence of elongate setae on the club and funicular articles, contiguous procoxae, a prosternal rostral channel with the lateral margins strongly converging and meeting below the procoxae, a mesoventrite with a transverse ventrally produced ridge anterior to mesocoxae and the mesoventrite posterior to ridge with dense yellow scales (Fig. 1), and the presence of stridulatory plectra on the male seventh abdominal tergite. Funicular article 1 is short and globose, the hind femora are not carinate and ventrally with one or no teeth, the femoral apices are always unarmed at the lateral and mesal faces, the eyes of most species have a distinct lower constriction lateral to rostral insertion (Fig. 37), the abdominal ventrites are slightly ascending, and the pygidium is concealed (slightly exposed in male *T. spinolae* and male of at least 1 undescribed species).

#### Notes.

The contiguous procoxae efficiently separates *Trichodocerus* from all genera except some species of the very different *Zygops*. The yellow scales of the mesocoxae and mesoventrite have also been observed in *Arachnomorpha* Champion, 1906 (on the pro- and mesocoxae), *Microzygops* Champion, 1906 (on the procoxae), and some species of *Lechriops* and *Macrocopturus* (on the pro- and mesocoxae and ventrally on the mesofemora), but in those genera they are not also present on the mesoventrite between the mesocoxae.

#### Phylogenetic relationships.

The numerous features that easily separate *Trichodocerus* from the rest of the conoderines are potentially indicative of improper placement in the Conoderinae, however, the same unique characters prevent confident reassignment to another group of Curculionidae. Champion (1906: 713) noted the similarity of the antennal funicle to *Hedycera* Pascoe, 1870, a South American genus here reassigned to the Piazurini.

#### Host associations.

The genus is apparently nocturnal, with most observed specimens being collected at UV light traps – 488 specimens were collected by Wolda et al. (1998), representing three of the 17 species of Conoderinae with more than 10 specimens collected. One undescribed species has been reared from balsa, *Ochroma pyramidale* (Cav. ex Lam.) Urb. (Malvaceae: Bombacoideae Burnett) (Hespenheide, personal communication). An observed specimen of *T. brevilineatus* Champion, 1906 [STRI_ENT_008474] was recorded from dead branches of balsa and an undescribed species [SSAC0001000] was collected on balsa leaves; another observed undescribed species has been collected on dead branches of *Pachira sessilis* Benth. (Bombacoideae) [STRI_ENT_0084793].

#### Described species.

Two species are known from the focal region and one additional described species occurs in South America. I have accumulated and distinguished over 20 species of *Trichodocerus* new to science as part of a revision currently in preparation.

#### Range.

Guatemala, Costa Rica, Panama; South America. Undescribed species are also known from Mexico.

### Piazurini

Taxon classificationAnimaliaColeopteraCurculionidae

Lacordaire, 1865: 144

#### Classificatory history and current circumscription.

This tribe was originally characterized by Lacordaire (1865: 144) for the genera *Cratosomus*, *Pinarus*, and *Piazurus* in recognition of the strongly canaliculate prosternum, the “gutter-like” modification to the mesoventrite and the clavate, non-carinate hind femora that do not or only slightly exceed the abdominal apex. Heller (1906: 31) produced a key to Piazurini that includes 8 of the 12 currently recognized genera – not included are *Lobops*, *Latychellus* Hustache, 1938, *Hedycera*, and the Old World *Guiomatus* – based largely on the relative size of abdominal ventrites and the amount that they ascend, relative lengths of funicular articles, and the construction of the mesoventrite.

The monotypic South American genus *Hedycera* is moved to the Piazurini despite the occurrence of the genus outside the geographic focus of this paper. The exposed pygidium that is not completely visible in dorsal view, the large triangular tooth on the hind femur, the transverse posteromedial depression on the metaventrite (discussed further below), and the unarmed femoral apices place the genus not only in the Piazurini but in a hypothesized clade containing *Piazurus*, *Pseudopiazurus* Heller, 1906, *Pseudopinarus* Heller, 1906 and the South American *Piazolechriops* Heller, 1906. *Hedycera megamera* Pascoe, 1870 would key out to couplet 7 of Heller’s 1906 key (containing *Pseudopinarus* and *Piazolechriops*), for having abdominal segments only slightly ascending, abdominal segment 2 not being longer than 3 and 4 combined, and the presence of “*superciliarleisten*”, referring to the arcuate carina at the vertex of the head found in most members of these genera (though not in a few species of *Pseudopinarus*), a greatly elongate antennal funicular article 2, and a slender rostrum. *Hedycera* can be differentiated from these by the shape of the pronotum in dorsal view, which is widest in the anterior half just before the subapical constriction, and in having elongate setae on the antennal funicular articles. When originally describing the monotypic genus, Pascoe (1870) stated that it was most closely related to *Piazurus*, which was later agreed with by Champion (1906: 713). *Hedycera* is the first genus separated in Heller’s key (1895) for having similar-sized abdominal ventrites 2, 3, and 4, but is not treated further in that publication. In the catalogs of Hustache (1934: 45) and Blackwelder (1947: 884) *Hedycera* is listed under the otherwise entirely Old World-distributed conoderine tribe Mecopini Lacordaire, 1865 and was moved to Lechriopini in Wibmer and O’Brien (1986: 19), without a justification provided in either placement.

#### Variation in key character systems.

The modification to the mesoventrite in the genera treated here in the Piazurini varies from being a cup-shaped receptacle (as in *Lobops*; Fig. 2) to structured similarly to a cup-shaped receptacle but with the posterior margin flattened and depressed at least slightly below the level of the lateral margins of the channel (Figs 3, 4) allowing the rostrum to pass through to the metaventrite if long enough. The eyes are often smaller and more separated and are not or not as sharply acuminate ventrally or laterally inflexed (Figs 38, 39) as in many Lechriopini and Zygopini, but can be quite large and contiguous or subcontiguous (Figs 40–41), taking up most of the surface of the head as well as be slightly ventrally acuminate to slightly laterally inflexed. The pygidium is exposed but not entirely visible in dorsal view (somewhat concealed from above by the elytral apex; e.g. Fig. 68), usually only visible completely in posterior or ventral view. Abdominal ventrites are flat to slightly, evenly ascending. The vestiture consists of thick setae to small scales, usually not covering most of the body surface except in *Lobops*, which has large, flat and round scales. The femora are at least slightly clavate, the hind femur is without a lateral carina and lacks teeth at the mesal and lateral apical faces (Fig. 60; in most lechriopines and zygopines, a tooth is usually present at the mesal and/or lateral face of the femoral apex on the middle and/or hind legs as in Figs 61–63), and several genera have a large, laterally compressed, triangular ventral tooth. This large triangular tooth is also found in other conoderine tribes (e.g. Menemachini Lacordaire, 1865; Campyloscelini Schoenherr, 1845) as well as other groups of weevils (e.g. Hylobiini Kirby, 1837). Despite this homoplasious distribution in Curculionidae it likely represents a single origin within the Piazurini, with the genera having it also sharing additional characters; it is also not found in other New World Conoderinae, making it useful for diagnosing the group of Piazurines that bear it.

#### Additional characters of potential phylogenetic significance.

The metaventrite posteromedially has a transverse depression, not with a narrow longitudinal sulcus extending variably anteriorly as in most Lechriopini and Zygopini (but many species of *Cratosomus* have a broad longitudinal depression). The antennal club is typically more spherical to ovoid, with the suture between at least articles 2 and 3 sinuate (but also found in a few lechriopines and zygopines). A mesal process of the procoxae is absent in most piazurines and found in many lechriopines and zygopines (though present, among the Central American species observed, in *Pseudopinarus*, *Lobops bonvouloiri* (Hustache, 1932), and in the species *Piazurus alternans* Kirsch, 1875). Sclerolepidia are absent in Piazurini (Lyal et al. 2006: 237). Additionally, piazurines are quite different behaviorally from the remainder of the New World Conoderinae, typically being less active in the daytime and no species are known to be part of the several widespread mimicry complexes found in the tribes Lechriopini and Zygopini (Hespenheide 1995).

#### Diversity and distribution.

Fifty-two species are currently known from north of South America in five genera. Six additional genera are known only from South America, and one genus, *Guiomatus*, occurs in Papua New Guinea.

### Cratosomus

Taxon classificationAnimaliaColeopteraCurculionidae

Schoenherr, 1825: c.585

Figs 21 , 38 , 68

= Atenismus Chevrolat, 1880: L [Syn.: Emden 1933: 505]. Type species: Atenismus spinipennis Chevrolat, 1880 [by monotypy].
= Gorgus Schoenherr, 1825: c.585 [Syn.: Gyllenhal 1837: 13]. Type species: Cryptorhynchus lentiginosus Germar, 1824 [by original designation].
= Gorgus Schoenherr, 1826: 279 (non Schoenherr, 1825) [Syn.: Gyllenhal 1837: 32]. Type species: Curculio dubius Fabricius, 1787 [by original designation] (=Curculio bombina Fabricius, 1787).
(Eucratosomus) Kuschel, 1945: 361. Type species: Cryptorhynchus sticticus Germar, 1824 [by original designation].

#### Type species.

*Rhynchaenus herculeanus* Dalman, 1823 [by original designation] (=*Rhynchaenus roddami* Kirby, 1819).

#### Gender.

Masculine.

#### Diagnosis.

*Cratosomus* can be differentiated from the other Piazurines treated here by the setal tufts of the anterior margin of the tibial apex being composed of thick fascicles of golden setae (Fig. 21), the dense setal brush at the posterodistal face of the meso- and metatibia (Fig. 21), the thick rostrum that is apically dorsoventrally compressed, and generally larger body size. The eyes can be small and widely separated (Fig. 38) or large and approximate; the femora are ventrally with 0-2 teeth, usually with a distinct ventral carina distally; and the elytra and pronotum are often tuberculate or spinose.

#### Notes.

This genus includes some of the largest Neotropical weevils (Champion 1906: 1). Males of some species have lateral tusk-like processes of the rostrum and are presumably under sexual selection and used during male-male competitions.

#### Keys.

Emden 1933 (Central and South America), Champion 1906: 2 (Central America).

#### Phylogenetic relationships.

Schoenherr’s (1838) classification included *Cratosomus* in a separate *Cohors* of Cryptorhynchides, thereby distinguished from the rest of the then-described Conoderinae. Although somewhat dissimilar in appearance to the Piazurini treated here, it resembles the South American piazurine genera *Latychus* Pascoe, 1872 (and likely also the South American *Costolatychus* Heller, 1906, and *Latychellus* Hustache, 1938, but no specimens were observed of those genera) in the thickened dorsoventrally compressed rostral apex, the smaller and relatively widely separated eyes (as in some *Cratosomus*), and the small ventral femoral tooth.

#### Host associations.

R.S. Anderson (1993: 218) lists Annonaceae Juss., Rutaceae Juss., Lauraceae Juss., Myrtaceae Juss., and Sapotaceae Juss Hosts for several species of Brazilian *Cratosomus* are recorded by Costa-Lima (1956: 213).

#### Described species.

Twenty-five species are known from the focal region (with 14 subspecies or forms) and an additional 126 species (and many subspecies or forms) are known exclusively from South America (Wibmer and O’Brien 1986: 254; Rheinheimer 2011: 66 described one more).

#### Range.

Mexico, Belize, Guatemala, Honduras, Nicaragua, Costa Rica, Panama; South America. Distributions of Guadeloupe and the Lesser Antilles are listed with doubt by Emden (1933: 532) and subsequent catalogs.

### Lobops

Taxon classificationAnimaliaColeopteraCurculionidae

Schoenherr, 1845: 116

Figs 2 , 39 , 69

#### Type species.

*Lobops setosus* Fåhraeus, 1845 [by original designation].

#### Gender.

Masculine.

#### Diagnosis.

*Lobops* is unique among the Piazurines in having a prominent cup-shaped receptacle for receiving the rostrum on the mesoventrite (Fig. 2), a dense covering in flat, round scales, and strongly concave interocular space (Fig. 39). The metafemoral tooth is not especially large, the femora are not strongly clavate, and the second funicular article is relatively short (not longer than the first); these characters are also shared with *Cratosomus* but not other piazurine genera treated here.

#### Phylogenetic relationships.

Of the five piazurine genera covered here, *Lobops* has the least certain placement in the tribe. Schoenherr (1845) originally indicated for the South American type species a relationship with *Conotrachelus*, and the genus was previously placed in the Ithyporini Lacordaire, 1865 (O’Brien and Wibmer 1982: 125, as Cryptorhynchinae; the tribe is currently placed in the Molytinae), overlooking a transfer to the Piazurini by Kuschel (1955: 271). The only known Central American species, *L. bonvouloiri*, was originally described in the genus *Pseudopinarus*. The structure of the mesoventrite is suggestive of placement in the Cryptorhynchinae, but that type of receptacle has been shown to not be exclusive to the subfamily (Riedel et al. 2016: 5). *Lobops* is certainly better placed in the Piazurini than in the other tribes reviewed here – despite differences in a number of characters, the exposed pygidium that is not visible in dorsal view, the unarmed femoral apices, non-carinate femora, and lack of sclerolepidia, in combination, are unique to the Piazurini.

#### Host associations.

Unknown.

#### Described species.

One species is known from the focal region and two additional species are known from South America (Wibmer and O’Brien 1986: 263).

#### Range.

Panama; South America.

### Piazurus

Taxon classificationAnimaliaColeopteraCurculionidae

Schoenherr, 1825: c.586

Figs 3 , 23 , 60 , 70

#### Type species.

*Poecilma stipitosum* Germar, 1824 [by original designation].

#### Gender.

Masculine.

#### Diagnosis.

An elongate second funicular article and broad triangular femoral tooth place *Piazurus* near *Pseudopinarus* and *Pseudopiazurus*, and it can be differentiated from them by the longer second abdominal ventrite (which is as long as the third and fourth ventrites when seen from the side) and the protibial apex that bears a premucro. Being a much more diverse genus than *Pseudopinarus* and *Pseudopiazurus*, it is easiest to arrive at an identification by a negative identification of those two smaller genera: namely, species of *Piazurus* never have a carinate vertex of the head (as in many *Pseudopinarus*), a strongly impressed first abdominal ventrite (as in *Pseudopiazurus*), or a subapical premucro (as in some *Pseudopinarus* and *Pseudopiazurus*) and usually do not have a mesal procoxal process (which is found in most *Pseudopinarus* and a South American *Pseudopiazurus*; it is present at least in *Piazurus alternans*).

#### Notes.

Fiedler (1936) divided *Piazurus* into seven groups based mainly on the shape of the elytra and the presence, location and shape of elytral tubercules.

#### Keys.

Fiedler 1936 (Central and South America), Heller 1906: 33 (*Piazurus* *s. str.* of Central and South America), Champion 1906: 9 (Central America).

#### Phylogenetic relationships.

Of the genera with a broad ventral metafemoral tooth, *Piazurus* is most similar to *Pseudopiazurus* with a conical prothorax and lack of a mesal process of the procoxae (though it is present in at least one species of each genus).

#### Host associations.

Some species have been reared from branches of various genera of Lecythidaceae (Fassbender 2013, Fassbender et al. 2014). Maes and O’Brien (1990) report *Piazurus trifoveatus* Champion, 1906 from *Coffea* L. (Rubiaceae Juss.) and Costa-Lima (1956: 218) reports a Brazilian species from fruits of Myrtaceae.

#### Described species.

Nineteen species are known from the focal region and an additional 58 species are exclusive to South America (Wibmer and O’Brien 1986: 260).

#### Range.

Mexico, Guatemala, Belize, Honduras, Nicaragua, Costa Rica, Panama; South America.

### Pseudopiazurus

Taxon classificationAnimaliaColeopteraCurculionidae

Heller, 1906: 32

**Figures 71–74.**
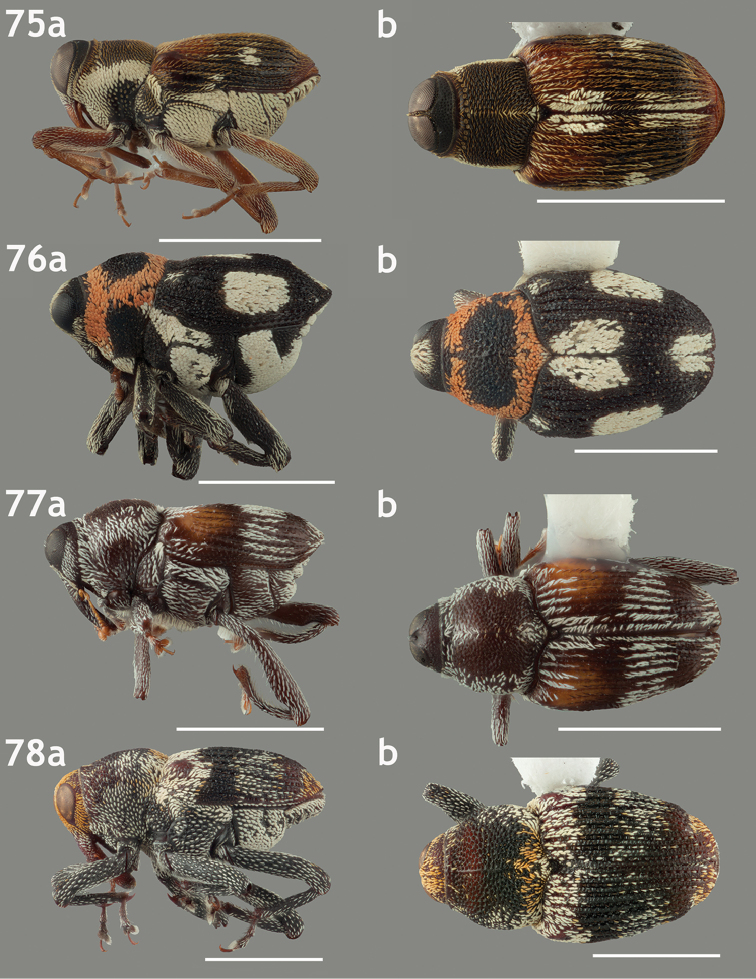
Lateral and dorsal habitus images of Piazurini, Othippiini and Lechriopini. Scale bars = 2 mm unless otherwise specified **71a–b** *Pseudopiazurus centraliamericanus* [ASUHIC0086627]; scale bars = 5 mm **72a–b** *Pseudopinarus condyliatus* [ASUHIC0086626]; scale bar for **72a** = 1 mm **73a–b** *Acoptus suturalis* [ASUHIC0016914] **74a–b** *Copturomimus cinereus* [ASUHIC0086628].

Figs 22 , 40 , 64 , 71

#### Type species.

*Piazurus obesus* Boheman, 1838 [by subsequent designation: Rheinheimer 2011: 76].

#### Gender.

Masculine.

#### Diagnosis.

*Pseudopiazurus centraliamericanus* (Heller, 1906), the only recorded Central American species of *Pseudopiazurus*, can be separated from other Central American piazurines by the deep U-shaped depression on the first abdominal ventrite (Fig. 64; also found in a few *Pseudopinarus*, but when present in that genus there is always also present the arcuate carina on the vertex of the head which is never found in *Pseudopiazurus*), the large subapical premucro of the metatibia (Fig. 22; but also at least in *Pseudopinarus guyanensis* Hustache, 1938), the absent premucro at the protibial apex (also in species of *Pseudopinarus*), the very large, ovoid, contiguous eyes (Fig. 40), and the large, deep, ovoid punctures of the elytral striae (but also found in some *Cratosomus* species).

#### Notes.

First described by Heller (1906) along with *Pseudopinarus* as subgenera of *Piazurus*. As documented by Champion (1906: 18), after the subgenus was first introduced in the key the name was erroneously switched with that of *Pseudopinarus* and the key to species of *Pseudopiazurus* is given under the name *Pseudopinarus* (Heller 1906: 34). The catalog of South American species by Wibmer and O’Brien (1986: 262) makes a correction to the two species given for Central America by O’Brien and Wibmer (1982: 160), which overlooked an error by Hustache (1934), leaving *P. centraliamericanus* the only species known from Central America.

#### Keys.

Marshall (1922: 69), Fiedler 1936: 28 and Heller 1906: 34 (under *Pseudopinarus*, in error) also contain keys to species.

#### Phylogenetic relationships.

This genus is most similar in overall appearance to *Piazurus* (see above), but the genus as a whole is incompletely distinguishable from *Pseudopinarus* by obvious characters with the exception of the much larger eyes, usually larger and more ascending mesepipleura, and absent ventral profemoral tooth of *Pseudopiazurus* – the deep arcuate sulci of the first ventrite in *Pseudopiazurus* is apparently also found in *Pseudopinarus*, e.g. in *Pseudopinarus quadratus* (Champion, 1906); the mesal process of the procoxae is absent in *Pseudopiazurus centraliamericanus* but present in the South American *Pseudopiazurus spiniventris* Marshall, 1922 as well as in many *Pseudopinarus*, and the subapical premucro of the metatibia is also found in *Pseudopinarus guyanensis*. Despite the overlap in these characters, *Pseudopinarus* is interpreted here as more closely related to the South American *Piazolechriops*, *Pinarus*, and *Hedycera*.

#### Host associations.

R.S. Anderson (1993: 218) lists Guttiferae Juss. (=Clusiaceae Lindl.). Marshall (1922: 67) records the South American *P. obesus* (=*P. papayanus* (Marshall, 1922)) as a borer of the “leaf-stems” of papaya (Caricaceae Dumort: *Carica papaya* L.).

#### Described species.

One species is known from the focal region and three additional species are known exclusively from South America (Wibmer and O’Brien 1986: 262, including Costa-Lima’s (1956: 217) synonymy of *P. papayanus* with *P. obesus*, which was either overlooked or disputed, as they are treated as separate by Wibmer and O’Brien).

#### Range.

Mexico, Guatemala, Honduras, Nicaragua, Costa Rica, Panama; South America.

### Pseudopinarus

Taxon classificationAnimaliaColeopteraCurculionidae

Heller, 1906: 33

Figs 4 , 41 , 72

= Paralatychus Voss, 1947: 60 [Syn.: Wibmer and O’Brien 1986: 7]. Type species: Paralatychus conotracheloides Voss, 1947 [by original designation] (=Piazurus dentipennis Fiedler, 1936).

#### Type species.

*Piazurus rana* Heller, 1906 [by subsequent designation: Rheinheimer 2011: 76].

#### Gender.

Masculine.

#### Diagnosis.

As a subgenus of *Piazurus*, *Pseudopinarus* was separated from *Piazurus* *s. str.*, along with the other subgenus, *Pseudopiazurus*, by Heller (1906) and Fiedler (1936) for having a second abdominal ventrite that is shorter in length than the third and fourth ventrites combined. *Pseudopiazurus* is easily distinguished from *Pseudopiazurus* in body shape, which is much more robust in *Pseudopiazurus*, but as a whole *Pseudopinarus* is the most difficult piazurine genus to characterize. Most species can be further distinguished from *Pseudopiazurus* by the presence of a ventral tooth on the profemora. Some *Pseudopinarus* have an arcuate carina on the vertex of the head (Fig. 41) similar to the South American *Piazolechriops* and *Hedycera*. The eyes are generally smaller and more separate than in *Pseudopiazurus*, but can be large and subcontiguous (as in Fig. 41). The structure of the mesoventrite varies as well, with some species bearing ventrally produced posterolateral tubercles (as in *Pseudopiazurus*) and others with nearly a cup-shaped receptacle. Additionally, the relatively small mesepipleura and the procoxae with a mesal process differentiates some *Pseudopinarus* from most species of *Piazurus* and the single Central American species of *Pseudopiazurus*.

#### Keys.

Fiedler 1936: 29, Heller 1906: 34.

#### Phylogenetic relationships.

The species of *Pseudopinarus* that have the arcuate carina on the vertex of the head are very similar to the South American genera *Piazolechriops* and *Hedycera* than to other Central American genera. The only character given by Heller (1906) to separate *Pseudopinarus* from *Piazolechriops* is the shorter hind femur of *Pseudopinarus*, which do not, or only very slightly, extend beyond the apex of the abdomen. *Pseudopinarus* differs from *Hedycera* by the short antennal setae and the pronotum in dorsal view, which is not widest just before the apex. Other species of *Pseudopinarus*, e.g. *P. cerastes* (Fabricius, 1801), are more similar to smaller species of *Piazurus* (e.g. *P. alternans*), raising the question of the monophyly of the genus and the validity of the shorter second abdominal ventrite as a character separating monophyletic groups from *Piazurus*.

#### Host associations.

Some species have been reared from branches of various genera of Lecythidaceae (Fassbender et al. 2014). *Pseudopinarus guyanensis* has been reared from seeds of *Gnetum* L. (Gnetaceae Blume) [ASUHIC0086636, STRI_ENT_0082031].

#### Described species.

Seven species are known from the focal region (Wibmer and O’Brien 1986: 263 add *P. guyanensis* to the Central American fauna) and an additional 13 species are known only from South America (Wibmer and O’Brien 1986: 262).

#### Range.

Mexico, Belize, Guatemala, Honduras, Nicaragua, Costa Rica, Panama; South America.

### Othippiini

Taxon classificationAnimaliaColeopteraCurculionidae

Morimoto, 1962: 47

#### Classificatory history and current circumscription.

Eleven genera were first grouped into tribe Othippiini by Hustache (1938: 63), which was not treated as a valid name in the catalog of Alonso-Zarazaga and Lyal (1999: 113) for lacking a description. Morimoto (1962: 47) provided some clarification on the distinction of the tribe, and this was further refined in Kojima and Lyal (2002), where seven genera were transferred out of the Othippiini in order to redefine it. Othippiines can be distinguished (*sensu* Kojima and Lyal 2002: 172) by the following combination of characters: the mesepisterna are non-ascending, the scutellum is exposed, the prosternum of most is canaliculate, and the antennal funiculus has 7 articles. However, the monophyly of the tribe has yet to be shown (Kojima and Lyal 2002).

#### Variation in key character systems.

The mesoventrite of othippiines can have a rostral channel or be unmodified (Kojima and Lyal 2002). The number of antennal funicular articles for othippiines given by Kojima and Lyal (2002) serves to separate this tribe from the Mecopini which have been considered to have 6 articles since originally described. However, one of the genera currently treated in the Mecopini (*Emexaure* Pascoe, 1871) has a funiculus with 7 articles (Pascoe 1871: 216), some of the genera are similar in appearance and also in eye shape (Kojima and Lyal 2002: 171) and distinction between the tribes requires further study.

#### Diversity and distribution.

Othippiini now contains eight genera, six of which are currently monotypic. The monotypic genus *Acoptus* is the sole New World representative.

### Acoptus

Taxon classificationAnimaliaColeopteraCurculionidae

LeConte, 1876: 264

Figs 5 , 24 , 42 , 73

= Homogaster Provancher, 1877: 530 [Syn.: Blackwelder and Blackwelder 1948: 48]. Type species: Homogaster quebecensis Provancher, 1877 [by monotypy].

#### Type species.

*Acoptus suturalis* LeConte, 1876 [by monotypy].

#### Gender.

Masculine.

#### Diagnosis.

*Acoptus* can be easily recognized from the rest of the conoderines treated here by the following characteristics: the inner margin of eyes towards the top has a large lanceolate space (Fig. 42) and the eyes are nearly touching above and below the lanceolate space, the mandibles are somewhat falcate and are in contact only at the apex (visible in Fig. 5), and the tibial apex distally has the dorsal margin dilated and premucro absent from all tibiae (Fig. 24). The relatively forward facing eyes that do not extend much on lateral portions of head (genae large), the first funicular article that is longer than the second, the unmodified mesoventrite, the ventrally toothed and non-carinate metafemora, the unarmed femoral apices, the small and non-ascending mesopleura, the absent sclerolepidia, and the abdominal ventrites that are not rapidly ascending additionally help diagnose the genus and in combination separate it from all Lechriopini and other New World Conoderinae.

#### Notes.

The species *Homogaster quebecensis* was first placed in synonymy with *Piazurus subfasciatus* LeConte, 1876 (=*Lechriops subfasciatus* (LeConte)) by LeConte (1880: xii), where it remained in catalogs until Blackwelder and Blackwelder (1948: 48) listed it in synonymy with *Acoptus suturalis*. Provancher’s description agrees with that of *Acoptus*.

#### Phylogenetic relationships.

In keys to North American genera, *Acoptus* is always separated from the rest of the North American genera along with *Psomus* by the flat abdominal ventrites (e.g. Hespenheide 2002: 755). Casey (1892: 458, 1897: 666) suggested a relationship to *Psomus* on these grounds, but this similarity only suggests they are both aberrant in their placement in the Lechriopini – they are otherwise very different in appearance, as also noted by Casey (1892: 458). LeConte (1876: 264) originally distinguished his genus from *Copturus* (which, with the geographic scope and time of publication of the key included only species currently placed in *Cylindrocopturus* and *Eulechriops*) and *Zygops* by the nearly flat abdominal ventrites and elongate first funicular article. Provancher (1877: 530), apparently independently, separated his genus *Homogaster* from *Zygops* and *Copturus* (as well as the South American *Timorus*) for the same reasons. A phylogeny by Davis (2014) recovered *Acoptus* as closer to the Old World genus *Mecopus* (Conoderinae: Mecopini) than any of the included New World conoderines, a result consistent with the new placement of the genus (the study did not include Othippiini).

#### Host associations.

Mentions in the literature of host plants include *Fagus* L. (Fagaceae Dumort) (Chittenden 1890: 171), *Ulmus americana* L. (Ulmaceae Mirb.) (Hoffman 1942: 12) and *Juglans cinerea* L. (Juglandaceae DC. ex Perleb) (Halik and Bergdahl 2002). Sleeper (1963: 215) additionally reports *Quercus* L. (Fagaceae), *Cercis canadensis* L. (Fabaceae Lindl.), *Carya* Nutt. (Juglandaceae), and *Platanus occidentalis* L. (Platanaceae T. Lestib.). Adults of *A. suturalis* have been implicated as vectors of the chestnut blight fungus, *Cryphonectria parasitica* (Murrill) Barr (Pakaluk and Anagnostakis 1977) and the butternut canker fungus, *Sirococcus clavigignenti-juglandacearum* Nair, Kostichka & Kuntz (Halik and Bergdahl 2002).

#### Described species.

One.

#### Range.

Eastern Canada, Eastern U.S.A., extreme northeastern Mexico (Sleeper 1963: 215). Specimens have not been observed from Mexico or even Texas to confirm Sleeper’s range extension; recently the genus was reported for the first time from Arkansas (Skvarla et al. 2015).

### Lechriopini

Taxon classificationAnimaliaColeopteraCurculionidae

Lacordaire, 1865: 149

#### Classificatory history and current circumscription.

This tribe was originally characterized by Lacordaire (1865: 149) for the genus *Lechriops* by the rostral channel, which is closed (horseshoe-shaped) posteriorly to receive the rostrum and the linear, carinate femora that may or may not exceed the apex of the abdomen.

While a subclassification for the Lechriopini is not formally proposed here without also examining the South American genera, the following groups of genera are hypothesized to be related: the “*Eulechriops* genus complex”, including *Eulechriops*, *Macrolechriops* Champion, 1906, *Copturomorpha* Champion, 1906, *Cylindrocopturinus* Sleeper, 1963, *Coturpus* R.S. Anderson, 1994, and *Turcopus* R.S. Anderson, 1994 and the “*Macrocopturus* genus complex”, including *Macrocopturus*, *Copturomimus* Heller, 1895, *Lechriops*, *Pseudolechriops*, *Hoplocopturus* Heller, 1895, and *Mnemynurus* Heller, 1895. The genera *Microzygops*, *Paramnemyne*, *Poecilogaster*, *Euzurus*, *Copturus*, *Microzurus* and *Psomus* do not fit into either complex as currently conceived. Until the inclusion of the South American lechriopine genera a subtribal classification for the Lechriopini will not be further speculated here.

#### Variation in key character systems.

Among the genera currently placed in the tribe (*sensu* Lyal et al. 2006), the only characters that distinguish them (after the exclusion of *Acoptus*, *Philinna*, and *Philides*) are a concealed pygidium with rapidly ascending abdominal sclerites, the presence of modification to the mesoventrite and/or the presence of sclerolepidia (just sclerolepidia in *Copturomimus*, most *Macrocopturus* and *Psomus*). The mesepipleura are usually large and somewhat ascending (except in *Paramnemyne* and *Psomus*). Other characters given by Lyal et al. (2006: 229) that separate lechriopines from zygopines are: “larger eyes, extending half-way or more down the side of the head; a longer rostrum, reaching at least the middle coxae; the middle and hind femora with the posterior distal margin extended into an acuminate projection extending beyond the anterior distal margin”, but these appear to be homoplastic – many lechriopines, especially some *Eulechriops* and related genera, have smaller eyes like many zygopines, and many zygopines have a similar femoral apex. The presence of a carina and ventral tooth on the hind femora, and the relative lengths of the first two funicular articles are potentially indicative of infratribal relationships; in the *Eulechriops* genus complex the hind femora are not carinate and unarmed ventrally and the second funicular article is at most subequal to the first, while in the *Macrocopturus* genus complex the hind femora are ventrally toothed and carinate and the second funicular article is longer than the first.

Modification to the meso- and metaventrite to receive the rostrum varies quite a bit in this group, with the typical forms (i.e. deviating the least from Lacordaire’s original tribal construction of a closed, horseshoe-shaped channel), being found in most members of the following genera: *Lechriops*, *Poecilogaster*, *Eulechriops*, *Macrolechriops*, *Copturomorpha*, *Coturpus*, *Turcopus*, *Copturus*, *Microzurus*, *Euzurus*, *Microzygops* and *Pseudolechriops*. These genera likely do not represent a monophyletic group, and the mode of closure (whether a simple depression or a strongly carinate apex of the channel) and the location of closure (on the mesoventrite or metaventrite) can vary significantly within genera. *Pseudolechriops* has arcuate lateral margins of the channel forming an ovoid carina that encircles a deep excavation on the mesoventrite and the anterior margin of the metaventrite (Fig. 13). A few species of the genus *Macrocopturus* (e.g. *M. albidus* Champion, 1906) and the genus *Microzygops* have a similarly constructed mesoventrite (Fig. 11) but the majority of *Macrocopturus* species and the very similar *Copturomimus* species have the unmodified “zygopine type” of mesoventrite. The mesoventrites of the genera *Hoplocopturus* and *Mnemynurus* are interpreted as of the lechriopine type, with the sides of the channel strongly arcuate and meeting medially, forming an inverted U-shaped carina that no longer appears to serve the function of receiving the rostrum (Fig. 9). *Paramnemyne*, *Euzurus*, *Copturus*, and *Microzurus* have a mesoventrite that would be classified here as the piazurine type (Heller 1895: 5 also notes the resemblance), with the rostral channel on the mesoventrite open (in *Paramnemyne* and *Euzurus*, Figs 8, 12) or closed (in *Copturus* and *Microzurus*, Fig. 6) and without anteriorly extending carinae; at least the latter three genera likely belong in the Lechriopini considering other characters. The mesoventrite of *Psomus* is unmodified.

#### Diversity and distribution.

Two hundred and forty-two species are currently known from north of South America in nineteen genera, comprising nearly half of the genus- and the majority of the species-level diversity of North and Central American Conoderinae. An additional eight genera are known only from South America.

### Copturomimus

Taxon classificationAnimaliaColeopteraCurculionidae

Heller, 1895: 63

Figs 66 , 74

#### Type species.

*Copturomimus cinereus* Heller, 1895 [by present designation].

#### Gender.

Masculine.

#### Diagnosis.

*Copturomimus* is similar to the large genus *Macrocopturus* with the elongate second funicular article, unmodified mesoventrite, and carinate and ventrally toothed hind femora, and can only be distinguished externally from that genus by the obliquely striolate area dorsally on the profemora (Fig. 66). The other genus with a striolate patch on the profemora, *Copturomorpha*, tends to have a striolate patch that is less obvious, being more finely striolate and more often concealed by scales; that genus otherwise is more similar to *Eulechriops*.

#### Notes.

The function of the striolate profemora is unknown – the first conoderine species described with it, *Copturomorpha musica* (Kirsch 1875b), was named, as the specific epithet suggests, for its hypothesized stridulatory function (Kirsch 1875b: 248). The function of the patch was instead suggested to be for antennal grooming purposes (Champion 1906b: 60) due to the lack of an obvious corresponding file structure required for stridulation and the position of the leg relative to the antennal club – observation of *Copturomimus caeruleotinctus* Champion, 1906 [SSAC0001059] revealed the use of the setal comb at the protibial apex (and not the striolate femoral patch) for antennal cleaning purposes.

#### Keys.

Champion 1906b: 60 (for Central America), Muñiz 1965: 5 (for three species on avocado, key modified from Muñiz and Barrera 1958: 2).

#### Phylogenetic relationships.

Heller (1895: 63) originally implied a relationship with his South American genus *Copturosomus* Heller, 1895, which is also difficult to distinguish from *Macrocopturus*. The relationship of both genera with *Macrocopturus* requires much more study to identify natural groupings of species. Whether the striolate femoral patch identifies a natural group is unknown but unlikely (Hespenheide 2009: 337). See also entry on *Macrocopturus* for discussion of the relationships of that hypothesized complex of genera.

#### Host association

. *Copturomimus perseae* (Guenther, 1935) and two other South American species are wood-boring on avocado (Lauraceae: *Persea* Mill.) (Hustache *in* Mariño M. 1947, Kissinger 1957, Muñiz 1965). Associations of other Central American species are unknown.

#### Described species.

Twelve species are known from the focal region (one species described by Hespenheide 2009) and five additional species are known from South America (Wibmer and O’Brien 1986: 271; Muñiz 1965 transferred one species from *Copturus*).

#### Range.

Mexico, Guatemala, Honduras, Costa Rica, Panama; South America.

### Copturomorpha

Taxon classificationAnimaliaColeopteraCurculionidae

Champion, 1906b: 65

Fig. 75

#### Type species.

*Copturomorpha interrupta* Champion, 1906 [by original designation].

#### Gender.

Feminine.

**Figures 75–78.**
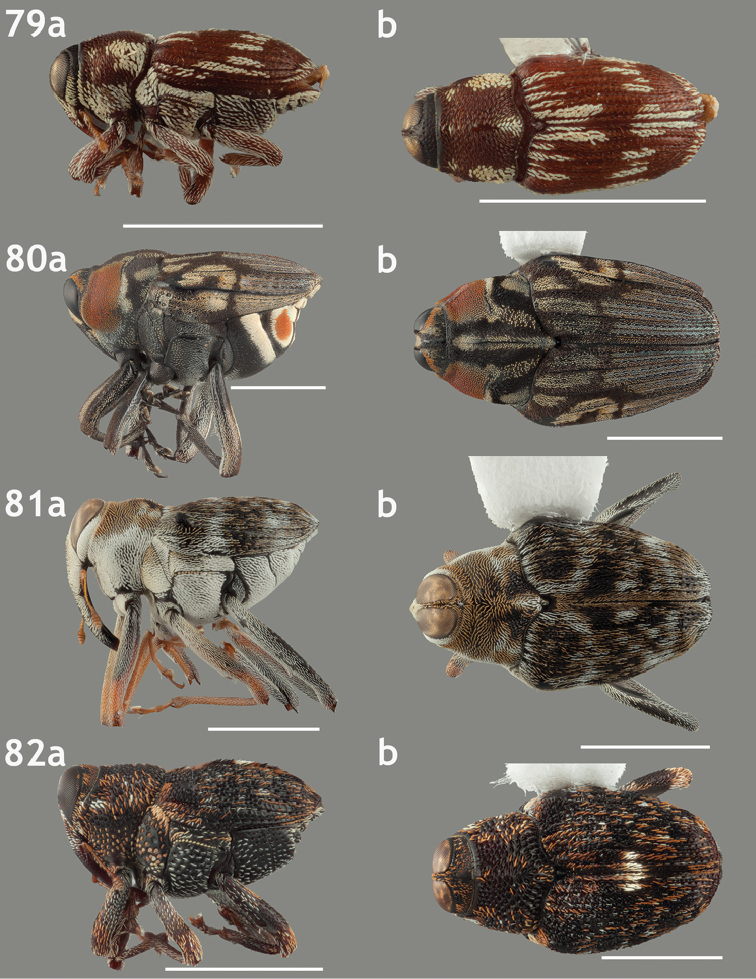
Lateral and dorsal habitus images of Lechriopini. **75a–b** *Copturomorpha* sp. [ASUHIC0086641] **76a–b** *Copturus aurivillianus* [ASUHIC0024140] **77a–b** *Coturpus arcuatus* [ARTSYS0000531] **78a–b** *Cylindrocopturinus pictus* [SSAC0001288]. Scale bars = 2 mm.

#### Diagnosis.

Like *Copturomimus*, *Copturomorpha* can only be distinguished from a much larger genus (*Eulechriops*) by the presence of a striolate patch dorsally on the profemur, and shares the short second funicular article that is not longer than the first, the unarmed and non-carinate metafemora and the excavate mesoventrite.

#### Notes.

The presence of the striolate patch on the dorsal surface of the profemora is shared only with *Copturomimus*, where the patch is typically larger and more coarsely striolate. *Copturomorpha* will key out to *Eulechriops* if that character is overlooked – it is often indistinct and obscured by scales. Several South American species described in the genus by Hustache (1938) have a second funicular article that is longer than the first and a carinate and toothed hind femora in addition to the excavated mesoventrite, necessitating further study on the identity of *Copturomorpha* and the species currently placed there.

#### Keys.

Champion 1906b: 65 (for Central America).

#### Phylogenetic relationships.

The combination of characters from the antenna, mesoventrite, and femora place *Copturomorpha* in a hypothesized genus complex including *Eulechriops*; whether the striolate femoral patch identifies a natural group separate from or within *Eulechriops* needs investigation.

#### Host associations.

Hosts of all described species are unknown; Fassbender (2013) and Fassbender et al. (2014) reared specimens from branches of Lecythidaceae that potentially represent a species of this genus.

#### Described species.

Eight species are known from the focal region (one species described by Hespenheide 2011) and an additional 16 species are known from South America (Wibmer and O’Brien 1986: 271).

#### Range.

U.S.A.: Texas, Mexico, Guatemala, Panama; South America.

### Copturus

Taxon classificationAnimaliaColeopteraCurculionidae

Schoenherr, 1825: c.586

Figs 6 , 76

= Zurus Heller, 1895: 5 (non Amyot, 1846). Type species: Zurus aurivillianus Heller, 1895 [by subsequent designation: O’Brien and Wibmer 1982: 8].
= Neozurus O’Brien & Wibmer, 1982: 168 [replacement name for Zurus] [Syn.: Wibmer & O’Brien 1986: 5].

#### Type species.

*Poecilma papaveratum* Germar, 1824 [by original designation].

#### Gender.

Masculine.

#### Diagnosis.

*Copturus* is very similar to *Microzurus* with a concealed scutellum and a closed receptacle of the mesoventrite that is laterally flanged near the apex (Fig. 6), and can be distinguished from *Microzurus* by the ventrally toothed pro- and mesofemora, larger tarsal claws, and flattened (not costate) elytral intervals. The first two funicular articles vary in length among the species, with the second article being longer than or subequal to the first (Champion 1906b: 87).

#### Notes.

The usage of this generic epithet has a particularly complicated history, as explained by Muñiz-Vélez and Ordóñez-Reséndiz (2010). The first usage of the name *Copturus* was as a subgenus of *Zygops* (Schoenherr 1825: col. 586), where the type species was designated as *Poecilma papaveratum* Germar, 1824. The subgenus was elevated to genus by Dejean (1835), and dozens of additional species were described to the genus (e.g. Schoenherr 1838, 1845, Kirsch 1875a, b). Heller (1895) created several genera out of specimens included in *Copturus* including the genus *Zurus* Heller, 1895 for the species of *Copturus* that have a concealed scutellum, second funicular article that is barely longer than the first, and a unique, horseshoe-shaped modification to the mesoventrite. Among the species moved from *Copturus* into *Zurus* was *Poecilma papaveratum*, which Heller recognized as the previously designated type species of *Copturus*. *Neozurus* O’Brien & Wibmer, 1982 was created as a replacement name for *Zurus* (O’Brien and Wibmer 1982: 4) which was preoccupied by *Zurus* Amyot, 1846 and was later synonymized with *Copturus* as an unjustified replacement name (Wibmer and O’Brien 1986, Alonso-Zarazaga and Lyal 1999: 111). As a result, all species treated as *Copturus* by Heller became newly recombined as *Macrocopturus*, one of Heller’s original subgenera of *Copturus* elevated to genus (Wibmer and O’Brien 1986: 17), and all species treated as *Zurus* by Heller and subsequent authors until Wibmer and O’Brien (1986) became newly recombined as *Copturus*, returning the type species *Poecilma papaveratum* to its original genus.

#### Keys.

Champion 1906: 87 (to *Zurus* of Central America), Heller 1895: 5 (to *Zurus* of Central and South America).

#### Phylogenetic relationships.

Hespenheide (1984: 315) suggests a relationship with *Microzurus*, *Euzurus*, and *Cylindrocopturinus*. Of those three, it is most similar to *Microzurus*, the only differences given by Champion (1906: 87) being the ventrally toothed pro- and mesofemora, the larger body size and comparatively proportionate tarsal claws. *Euzurus* also has a scutellum concealed by a posterior lobe of the pronotum, but the manner of it’s concealment differs from *Copturus* and *Microzurus*: where the posterior pronotal lobe of *Copturus* and *Microzurus* subducts the elytra, completely concealing the scutellum, in *Euzurus* the posterior lobe is only extended posteriorly and not below the elytral base, leaving the scutellum visible in posterior view. Despite this difference, Lyal et al. report Type II sclerolepidia to be present in *Copturus*, *Microzurus*, and *Euzurus*, which, among the sclerolepidia-bearing lechriopines, is only also known in the very different *Psomus*. See entry on *Cylindrocopturinus* for the present interpretation of the relationship of that genus. The relationship of *Copturus* and *Microzurus* within the Lechriopini and whether or not *Euzurus* is found to be the sister-genus is not easily hypothesized by the external characters examined thus far.

#### Host associations.

The widespread Central and South American species *Copturus aurivillianus* (Heller, 1895) is reported by Costa-Lima (1956: 219) to bore stems of *Canavalia* Adans., *Dolichos* L., and *Phaseolus* L. (Fabaceae) as larvae in Brazil.

#### Described species.

Six species are known from the focal region and an additional 27 species are known only in South America.

#### Range.

Mexico, Honduras, Nicaragua, Costa Rica, Panama, Guadeloupe; South America.

### Coturpus

Taxon classificationAnimaliaColeopteraCurculionidae

R.S. Anderson, 1994: 480

Fig. 77

#### Type species.

*Coturpus arcuatus* R.S. Anderson, 1994.

#### Gender.

Masculine.

#### Diagnosis.

Within the *Eulechriops* complex of genera, *Coturpus* can be identified by lacking a striolate profemoral patch (as in *Copturomimus*), lacking a very prominent receptacle on the mesoventrite (as in *Turcopus*), lacking the premucro-like inner flange at the tibial apex (as in *Cylindrocopturinus*), and can be differentiated from the observed species of *Eulechriops* by bearing elongate setae on the ventral surface of strongly arcuate hind legs at least in the males and by lacking a procoxal mesal tooth (though with the vast numbers of undescribed *Eulechriops* it is difficult to rule out the absence of this character from that genus).

#### Notes.

Females are unknown, and R.S. Anderson (1994: 482) suspects the modified hind legs to be found only in males as similar modification to the hind legs is known only in male *Cylindrocopturinus*.

#### Phylogenetic relationships.

R.S. Anderson (1994: 462) proposed a relationship to *Cylindrocopturinus* based on the presence of elongate setae on the ventral surface of the hind legs in males. This genus is difficult to separate from large and variable *Eulechriops. Coturpus* can be further separated from *Turcopus* and *Cylindrocopturinus* by genitalic characters given by R.S. Anderson (1994).

#### Host associations.

The genus has been collected on mistletoe, *Phoradendron* Nutt. (Santalaceae) on *Quercus* (R.S. Anderson 1994: 484).

#### Described species.

One (R.S. Anderson 1994).

#### Range.

Mexico.

### Cylindrocopturinus

Taxon classificationAnimaliaColeopteraCurculionidae

Sleeper, 1963: 218

Figs 7 , 25 , 43 , 61 , 78

#### Type species.

*Eulechriops pictus* Schaeffer, 1908 [by monotypy].

#### Gender.

Masculine.

#### Diagnosis.

*Cylindrocopturinus* can be differentiated from genera in the *Eulechriops* complex of genera by the tibial apex, which has a modified inner flange that resembles the premucro (Fig. 25), a rostral channel that is laterally carinate on the mesoventrite and not closed posteriorly by carina (Fig. 7), and no striolate profemoral patch (a similar mesoventrite has been seen in *Copturomorpha*, but members of this genus always have a striolate profemoral patch).

#### Notes.

Sleeper’s (1963) key to U.S. genera inexplicably contains two couplets that lead to *Cylindrocopturinus*: couplet 6a leads to genus “7. *Cylindricopturinus*, new genus” and couplet 8a leads to genus “7. *Cylindrocopturinus*, new genus”. Couplet 6a is where *C. pictus* Schaeffer actually would key out to (in order to reach couplet 8a, couplet 4a would have to be selected, which states that the mesoventrite is not excavated, leading to couplet 8, where *Cylindrocopturinus* is differentiated from *Cylindrocopturus* for *having* such excavation). The use of the epithet in couplet 6a is the first appearance of the name and is spelled differently than the usage in the remainder of the text. Subsequent authors (Kissinger 1964, Hespenheide 1984, Anderson 1994) did not choose among the original spellings as a First Reviser (International Code of Zoological Nomenclature article 24.2.3) but used the spelling “*Cylindrocopturinus*” in their work. *Cylindrocopturinus* is thus selected here as the correct original spelling, making “*Cylindricopturinus*” an unavailable name as an alternative original spelling.

#### Keys.

R.S. Anderson 1994: 463.

#### Phylogenetic relationships.

See “Phylogenetic relationship” section for *Coturpus*. Hespenheide (1984: 315) suggested a relationship of *Cylindrocopturinus* with *Zurus* (= *Copturus*), *Euzurus*, *Microzurus*, *Mnemyne* Pascoe, 1880, and *Paramnemyne*, citing the “...structure of the mesosternum and procoxae, the small size of the tarsal claws, and form of the antennae...” as indicative of a closer relationship to *Microzurus*. The genera *Cylindrocopturinus*, *Coturpus*, and *Turcopus* were proposed by R.S. Anderson (1994) to be related to *Eulechriops*. As interpreted here, those genera, plus *Macrolechriops* and *Copturomorpha*, compose a group of lechriopines, the majority of which contain a combination of the following characteristics: unarmed and non-carinate hind femora, a rostral channel defined laterally by carina, and a second antennal funicular article that is subequal to or shorter than the first.

#### Host associations.

Species of *Cylindrocopturinus* have been collected on various species of *Phoradendron* on species of *Quercus*, *Acacia* Mill. (Fabaceae), *Juniperus* L. (Cupressaceae Gray), and *Ipomoea* L. (Convolvulaceae Juss.) (R.S. Anderson 1994).

#### Described species.

Four (Hespenheide (1984) described one species, R.S. Anderson (1994) described two).

#### Range.

U.S.A.: AZ, Mexico, Honduras.

### Eulechriops

Taxon classificationAnimaliaColeopteraCurculionidae

Faust, 1896: 91

Fig. 79

= Zygomicrus Casey, 1897: 679 [Syn.: Champion, 1906b: 109 (with doubt); Blatchley and Leng 1916: 423]. Type species: Eccoptus minutus LeConte, 1824 [by monotypy].

#### Type species.

*Eulechriops erythroleucus* Faust, 1896 [by subsequent designation: Sleeper 1963: 215].

#### Gender.

Masculine.

**Figures 79–82.**
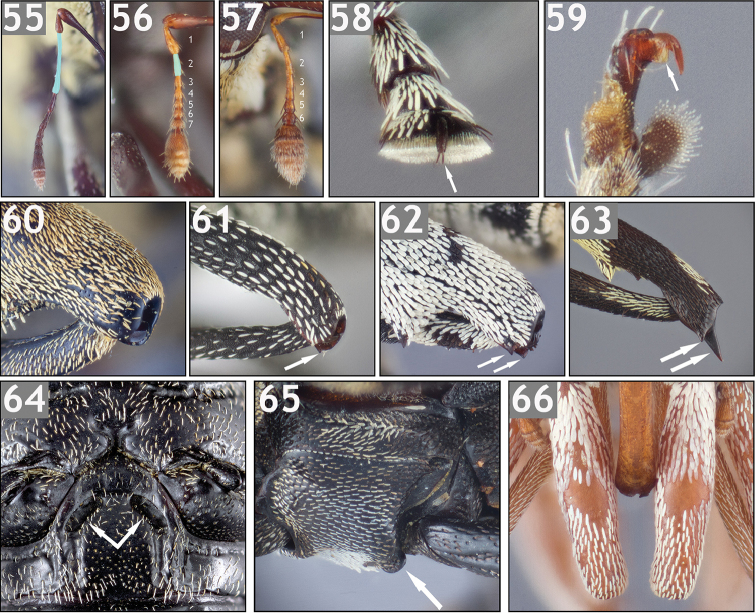
Lateral and dorsal habitus images of Lechriopini. **79a–b** *Eulechriops minutus* [ASUHIC0024145] **80a–b** *Euzurus ornativentris* [ARTSYS0000800]. **81a–b** *Hoplocopturus varipes* [SSAC0001086] **82a–b** *Lechriops vestitus* [SSAC0001114]. Scale bars = 2 mm.

#### Diagnosis.

*Eulechriops* is a large and variable genus that can be identified from related genera by the following combination of characters: antennal funicular article 2 is not longer than 1; the mesoventrite has a carinate channel that can terminate on the meso- or metaventrite, but when closed on the mesoventrite it is not pronounced and sharply carinate (as in *Turcopus*); the femora are not carinate and are ventrally unarmed; the profemora lack a striolate patch (as in *Copturomorpha*); the pronotum lacks a strongly convex, arcuate pronotal carina (as in *Macrolechriops*), and the hind legs are not sexually dimorphic (as in *Cylindrocopturinus* and *Coturpus*).

#### Notes.

The genus was erected by Faust (1896: 91, in footnote) to accommodate *Lechriops* that have unarmed, non-carinate femora; Marshall (1922: 70) notes the lack of interdependence of these two characters. The type species was not originally designated by Faust and is given by Sleeper (1963) as the Venezuelan species *E. erythroleucus*, which was the first of three species described by Faust under his new genus – this was apparently overlooked in subsequent catalogs where the type species is listed as undesignated. *Eulechriops* is possibly the most diverse genus of Conoderine when considering the large number of undescribed species (Hespenheide 2007), as well as the most speciose genus of Conoderinae in the Caribbean region with sixteen species described by Hustache (1932a) from Guadeloupe.

#### Keys.

Champion 1906b: 110 (for Central America), Hustache 1931: 285 (for Guadeloupe), Hespenheide 2003: 95 (for two U.S. species).

#### Phylogenetic relationships.

The genus is likely related to the genera *Copturomorpha*, *Macrolechriops*, *Cylindrocopturinus*, *Turcopus*, and *Coturpus*, most of which have a combination of the following characters: unarmed, non-carinate metafemora, a funicular article 2 that is not longer than article 1, and the rostral channel of the mesoventrite with relatively parallel longitudinal carinae. The species recognized as *Eulechriops* are unlikely to represent a monophyletic group (Hespenheide 2005b) and the species currently recognized in the genus can only be identified to *Eulechriops* by not having the distinguishing characters of the aforementioned related (and smaller) genera.

#### Host associations.

Mostly unknown. Two of the three U.S. species and related Mexican species (of the *E. minutus* species group of Hespenheide 2003) are associated with *Quercus* (Sleeper 1963, Hespenheide 2003). Several undetermined Central American species have been reared from *Cecropia*, *Coussapoa* Aubl. and *Pourouma* Aubl. (Urticaceae) (Jordal and Kirkendall 1998: 159, LaPierre 2002). Some South American species have been reared from *Rubus* L. (Rosaceae Juss.) (Hespenheide 2005), *Manihot* Mill. (Euphorbiaceae Juss.) (Monte 1938) and *Gossypium* L. (Malvaceae) (Barber 1926).

#### Described species.

Fifty-seven species are known from the focal region (with one described by Hespenheide 2007 and one fossil species described by Poinar and Legalov 2013) and an additional 31 species are known from South America (Wibmer and O’Brien 1986: 264, with one more described by Hespenheide 2005b).

#### Range.

U.S.A., Mexico, Belize, Guatemala, Honduras, Nicaragua, Costa Rica, Panama, Dominican Republic (Poinar and Legalov 2013, fossil), Guadeloupe; South America.

### Euzurus

Taxon classificationAnimaliaColeopteraCurculionidae

Champion, 1906b: 45

Figs 8 , 65 , 80

#### Type species.

*Euzurus ornativentris* Champion, 1906 [by original designation].

#### Gender.

Masculine.

#### Diagnosis.

The single species of *Euzurus* can be identified by the posteriorly produced lobe of the pronotum concealing the scutellum from above, the distinctly structured mesoventrite (Fig. 8) which has lamellae extending anteriorly from the posterior modification, and the large tubercle on the metaventrite anterior to the anteroventral border of the metacoxa (Fig. 65). The second antennal funicular article is longer than the first, the eyes are vertical and separated, the metafemur is laterally bicarinate and ventrally toothed, and the tibial uncus is short and curved.

#### Phylogenetic relationships.

With the genera *Copturus* and *Microzurus*, *Euzurus* shares a concealed scutellum (but see “Phylogenetic relationships” section for *Copturus*), Type II sclerolepidia (Lyal et al. 2006: 229), and modification to the mesoventrite that does not have anteriorly extending carinae. The separated, vertical eyes are similar to some species of *Cylindrocopturus* and *Poecilogaster* (Figs 46, 49). Champion (1906b: 46) mentions a similar appearance to Macrocopturus (Eucopturus) Heller.

#### Host associations.

Unknown.

#### Described species.

One.

#### Range.

Costa Rica, Panama.

### Hoplocopturus

Taxon classificationAnimaliaColeopteraCurculionidae

Heller, 1895: 50

**Figures 55–66.**
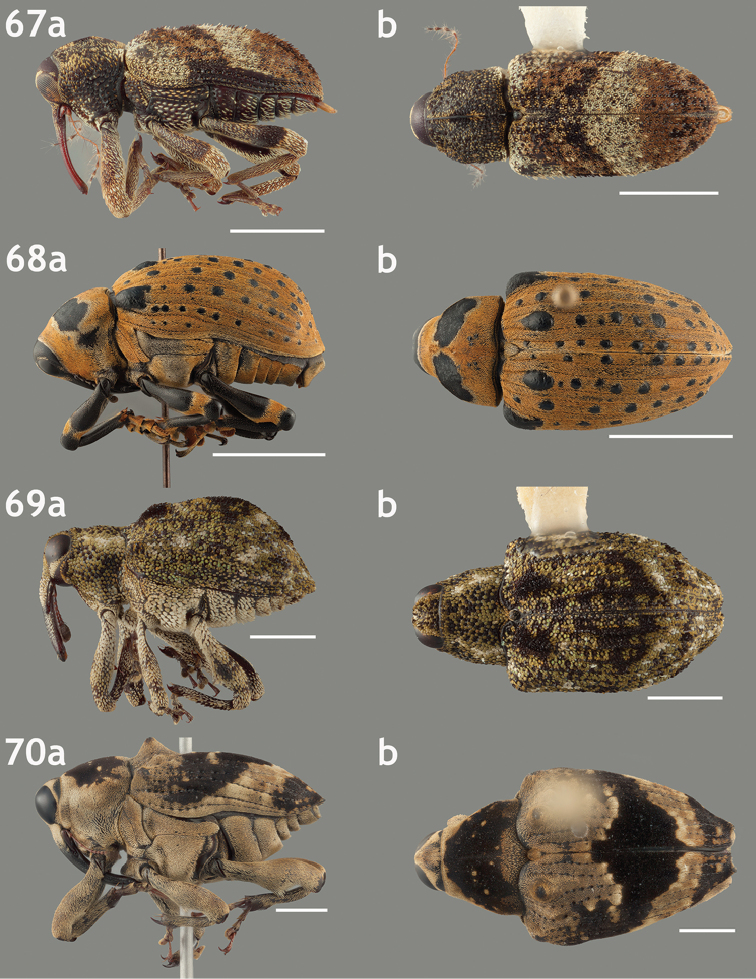
Miscellaneous morphological structures. **55** *Hoplocopturus sulphureus* [ARTSYS0000801] with an overlay showing an elongate second funicular article that is more than twice the length of the first **56** *Lissoderes subnudus* [SSAC0001064] with an overlay showing a second funicular article that is subequal to the first, and seven distinct funicular articles **57** *Philinna bicristata* [ARTSYS0000799] showing a funiculus composed of six articles **58** *Microzurus* sp. [SSAC0001290] showing a short, slender fifth tarsal article and minute claws **59** *Philides comans* [ARTSYS0000804] with a broad tooth at the base of the tarsal claw **60** Left metafemoral apex of *Piazurus trifoveatus* [SSAC0001118] that is unarmed at the mesal and lateral faces **61** Left metafemoral apex of *Cylindrocopturinus pictus* [SSAC0001288] with a tooth only at the mesal face **62** Left metafemoral apex of *Peltophorus polymitus seminiveus* [SSAC0001117] showing a small tooth at both mesal and lateral faces **63** Left metafemoral apex of *Hoplocopturus sulphureus* [ARTSYS0000801] showing a small tooth at the lateral face and an acuminate process at the mesal face **64** Metaventrite of *Pseudopiazurus centraliamericanus* [SSAC0001291] showing deep, arcuate sulc **65** Lateral view of the metathorax of *Euzurus ornativentris* [ARTSYS0000796] showing a large tubercle in anterior to the metacoxa **66** Dorsal view of the profemora of *Copturomimus caeruleotinctus* [SSAC0001059] showing denuded, striolate regions.

Figs 9 , 55 , 63 , 81

#### Type species.

*Copturus armatus* Gyllenhal, 1838 [by original designation].

#### Gender.

Masculine.

#### Diagnosis.

As originally distinguished from the rest of the New World conoderines by Heller (1895) in his key to genera, *Hoplocopturus*, *Mnemynurus*, and the South American *Balaninurus* Heller, 1895 have an arcuate carina on the mesoventrite. This character separates *Hoplocopturus* and *Mnemynurus* from the Central American lechriopine genera that have an elongate second funicular article, carinate and ventrally toothed femora and modification to the mesoventrite, especially the often very similar looking species of *Lechriops* and *Macrocopturus*. From *Mnemynurus caloderes* Heller, 1895 (the only species of *Mnemynurus* at the time), Heller (1895) differentiated *Hoplocopturus* by the length of the rostrum and the corresponding rostral channel – in his key, *Hoplocopturus* species have a rostrum that does not extend beyong the mesocoxae, while *Mnemynurus caloderes* has a rostrum channel and rostrum that extends to the first abdominal ventrite. Champion (1906b) added several species to each genus, mentioning that “*Hoplocopturus* is connected to *Mnemynurus* by intermediate forms, and it can only be separated therefrom by the shorter rostrum” (Champion 1906b: 53). Most species of *Mnemynurus* described by Champion (except *M. longispinis* Champion, 1906) have a rostrum that extends at least to the posterior margin of the metaventrite, and species of *Hoplocopturus* (except *H. javeti* Champion, 1906 and *H. nigripes* Champion, 1906) have a shorter rostrum. Those species of *Hoplocopturus* with a rostrum that reaches near the middle of the metaventrite are additionally difficult to separate from *Mnemynurus* because they have a similar coloration to all described *Mnemynurus* (the “red-eyed fly” mimicry complex) and the region of the mesoventrite posterior to the arcuate carina is invaginated under the carina (not simply a semicircular depression as in other *Hoplocopturus*). Despite these exceptions, the following characters can be used to separate many of the species of *Hoplocopturus* from *Mnemynurus*: rostrum shorter (never reaching the posterior margin of the metaventrite) and the rostral apex cylindrical (apically flattened and dilated in *Mnemynurus*). The hind femora of both genera can be carinate or not and the femoral apex typically has an elongate spine at the mesal face.

#### Notes.

Some species (e.g. *H. varipes* Champion, 1906) have a mesoventrite densely covered in scales and the distinguishing carina is difficult to see. Most species of *Hoplocopturus* are not clear members of a mimicry complex with the exception of a few species belonging to the “blue-thorax” complex and two described and several undescribed belonging to the “red-headed” fly-mimicking complex (Hespenheide 2005), but all described (and almost all observed undescribed) species of *Mnemynurus* belong to the “red-headed fly” complex. The species that have the “red-headed fly” coloration pattern are the most difficult to separate from *Mnemynurus*.

#### Keys.

Champion 1906: 53 (for Central America).

#### Phylogenetic relationships.

*Hoplocopturus* is very similar to *Mnemynurus* and *Balaninurus*, sharing with those genera the arcuate carina on the mesoventrite. Some species are very similar looking to certain *Lechriops* and *Macrocopturus* (e.g. *H. sherrywernerorum* Hespenheide, 2009 and *H. costatipennis* Champion, 1906, respectively), but the structure of the mesoventrite easily separates the species of *Hoplocopturus* from those genera.

#### Host associations.

Some *Hoplocopturus* (e.g. *H. varipes* Champion, 1906) can be found on the upper and lower surface of the large leaves of *Xanthosoma* Schott (Araceae Juss.) [SSAC0001086], a plant family association with Araceae has also been made in *Mnemynurus* (see below). One species has been collected on treefalls of *Sterculia* L. (Malvaceae: Sterculioideae Burnett) [STRI_ENT_0082473, SSAC0001292].

#### Described species.

Eighteen species are known from the focal region (with one more described by Hespenheide 2009) and an additional 12 species are known only in South America (Wibmer and O’Brien 1986: 270).

#### Range.

Mexico, Guatemala, Belize, Honduras, Nicaragua, Costa Rica, Panama; South America.

### Lechriops

Taxon classificationAnimaliaColeopteraCurculionidae

Schoenherr, 1825: c.586

Figs 10 , 19 , 82

= Gelus Casey, 1897: 667 [Syn.: Champion, 1906: 91]. Type species: Cryptorhynchus oculatus Say, 1824 [by subsequent designation: Sleeper 1963: 210].

#### Type species.

*Rhynchaenus sciurus* Fabricius, 1801 [by original designation].

#### Gender.

Masculine.

#### Diagnosis.

Most species of *Lechriops* can be distinguished by the following combination of characters: the second antennal funicular article is longer than the first, the mesoventrite has a rostral channel that is bordered laterally by carinae, and the metafemora are carinate and ventrally toothed (Champion 1906b: 91). The anterior margin of the metaventrite is also usually excavated to receive the apex of the rostrum (Fig. 10; Champion 1906b: 91, Hespenheide 2009: 334), and the region of the mesoventrite lateral to the longitudinal carinae is often with dense multifid setae.

#### Notes.

Many species have a white elytral sutural spot (as in Fig. 82b), but this is not exclusive to *Lechriops* (see Hespenheide 2009).

#### Keys.

See Hespenheide 2003: 351 (for the seven U.S. species) and Champion 1906b: 91 (for Central America). Also Sleeper 1963: 210 (for U.S. species), Blatchley and Leng 1916: 418 (for Northeastern U.S. species, as *Gelus*) and LeConte and Horn 1876: 260 (for U.S. species, as *Piazurus*).

#### Phylogenetic relationships.

Some species look superficially very similar to species of *Eulechriops*, *Macrocopturus*, and *Hoplocopturus*, but the above combination of characters will separate most species. Champion (1906b: 91) considered *Lechriops* to be very close to *Macrocopturus* and in both genera he described species similar to the other genus (*L. copturoides* Champion, 1906 and *Macrocopturus furfuraceus* (Champion, 1906), the latter of which “forms a sort of connecting-link between *Copturus* [=*Macrocopturus*] and *Lechriops*” (Champion 1906b: 69)).

#### Host associations.

Some species in the U.S. and Mexico (the *L. californicus* species group of Hespenheide 2003) are associated with various species of conifers in the genera *Pinus* L. and *Pseudotsuga* Carrière (Pinaceae Spreng. ex Rudolphi). Some Central American species have been reared from petioles and stems of *Cecropia* and *Coussapoa* (Urticaceae) (Jordal and Kirkendall 1998: 159, LaPierre 2002). The Puerto Rican *Lechriops psidii* Marshall, 1922 is known to feed on guava fruits (Myrtaceae: *Psidium guajava* L.) (Marshall 1922: 70), but the placement of that species in *Lechriops* is suspect (though no specimens have been observed) due to the unmodified mesoventrite and lack of a femoral tooth.

#### Described species.

Forty-nine species are known from the focal region [including two more described by Hespenheide 2003] and an additional 42 species are known exclusively from South America (Wibmer and O’Brien 1986: 263, including four more described by Rheinheimer 2011].

#### Range.

Canada, U.S.A., Mexico, Belize, Guatemala, El Salvador, Honduras, Nicaragua, Costa Rica, Panama, Puerto Rico, Guadeloupe; South America. A new species of *Lechriops* was recently described from India (Khairmode and Sathe 2015), though the position of the species in this genus or in the tribe Lechriopini is doubtful.

### Macrocopturus

Taxon classificationAnimaliaColeopteraCurculionidae

Heller, 1895: 19

Figs 44 , 83

(Macrocopturus) Heller, 1895: 19 [as subgenus of Copturus]. Type species: Not yet designated.
(Cyphocopturus) Heller, 1895: 19 [as subgenus of Copturus]. Type species: Not yet designated.
(Eucopturus) Heller, 1895: 20 [as subgenus of Copturus]. Type species: Not yet designated.
(Lamellocopturus) Heller, 1895: 19 [as subgenus of Copturus]. Type species: Not yet designated.

#### Type species.

*Copturus satyrus* Gyllenhal, 1838 [by subsequent designation: Wibmer and O’Brien 1986: 20].

#### Gender.

Masculine.

**Figures 83–86.**
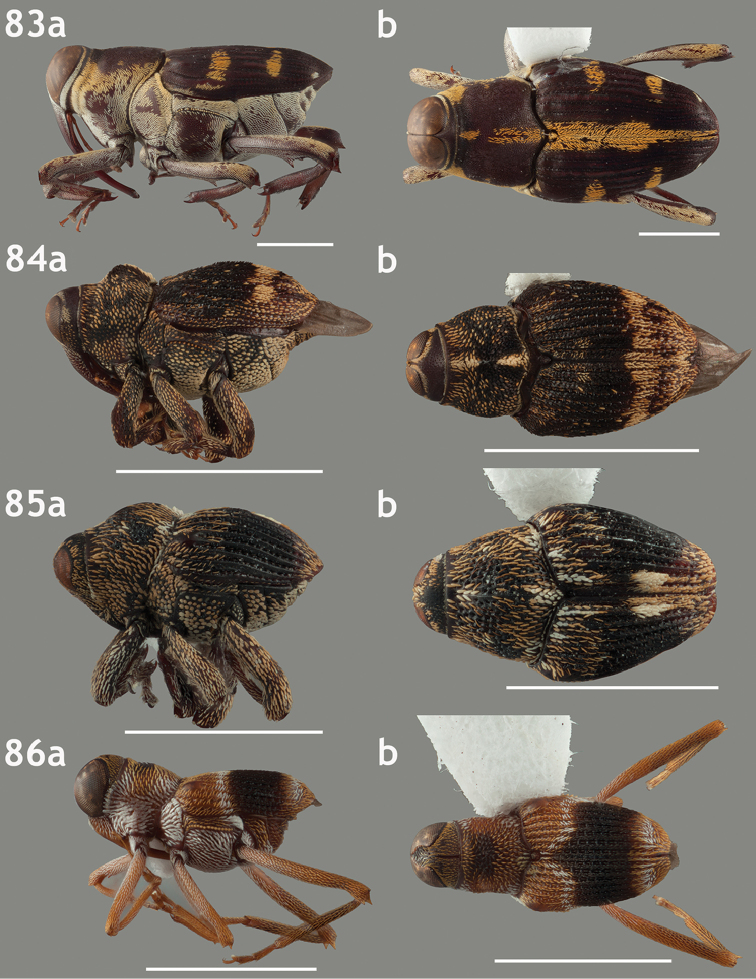
Lateral and dorsal habitus images of Lechriopini. **83a–b** *Macrocopturus lynceus* [SSAC0001085] **84a–b** *Macrolechriops spinicoxis* [ARTSYS0000529]. **85a–b** *Microzurus championi* [ASUHIC0031507] **86a–b** *Microzygops nigrofasciatus* [ARTSYS0000802]. Scale bars = 2 mm.

#### Diagnosis.

Most of the specimens of this very large and variable genus can be identified by the combination of a second funicular article that is longer than the first, an unmodified mesoventrite, a carinate and ventrally toothed hind femur, and the absence of a profemoral striolate patch. A few Central American species (and several more in South America – Heller’s subgenera *Lamellocopturus* and *Cyphocopturus*) have modification to the mesoventrite similar to *Microzygops* (e.g. *Macrocopturus albidus* (Champion, 1906)) but Champion did not assign them to one of Heller’s subgenera. Some smaller species can be difficult to distinguish from *Lechriops* but species of that genus in general tend to have a less spherical head (somewhat obliquely flattened above the rostral base) with comparatively smaller, more strongly acuminate eyes in addition to the modification to the mesoventrite.

#### Notes.

All species included in this genus were treated as *Copturus* until Wibmer and O’Brien (1986: 17), see “Notes” section for *Copturus* above for a clarification of name use. Heller (1895) divided the genus *Copturus* into six subgenera or species groups (including *Macrocopturus*), reiterating Pascoe’s (1880: 494) comment on his own struggle with adequately constructing a subgeneric classification for this genus: “*Copturus* illustrates the difficulty of defining a large genus. Every character is liable to exception, not one appearing to have a generic value, although there is a common interresemblance which is not to be mistaken; the group, in fact, is a natural one, but which perhaps might, for the advantage of the systematist, be artificially divided into several genera.” Heller noted the probable superficiality of his subgenera.

*Macrocopturus* is the most widespread genus in the Caribbean, being the only genus of Conoderinae recorded from the Bahamas and Jamaica. Four different putative mimicry complexes are present in *Macrocopturus* as presently defined (Hespenheide 1995). Immature stages are described for *Macrocopturus aguacatae* (Kissinger, 1957) by Muñiz Vélez (1958) and *M. burserophagus* Muñiz-Vélez & Ordóñez-Reséndiz, 2010 by Muñiz-Vélez and Ordóñez-Reséndiz (2010).

#### Phylogenetic relationships.

Some of the described species are very similar in appearance to the following genera: *Cylindrocopturus* (e.g. the Mexican *M. burserophagus* which is placed in *Macrocopturus* because of the presence of a ventral femoral tooth), *Copturomimus* (which have a striolate region on the profemora), *Lechriops*, *Hoplocopturus*, and the South American genera *Damurus* Heller, 1895 (Champion 1906b: 69, in footnote) and *Copturosomus* (Champion 1906b: 69). Of those genera, *Copturomimus*, *Lechriops*, and *Hoplocopturus* are considered related in this paper, and *Cylindrocopturus*, which is currently in the Zygopini, is also probably a related lechriopine. The few observed specimens of South American *Copturosomus* are very similar to *Macrocopturus* and the genus *Damurus* was not observed in the course of this study, but was considered by Heller (1895: 55) to be related to *Timorus* (also currently in the Zygopini) which Champion (1906: 33) in turn considered related to *Macrocopturus*.

#### Keys.

Champion 1906b: 69 (for Central America) and Heller 1895: 19 (for Central and South America).

#### Host associations.

*Macrocopturus floridanus* (Fall, 1906), known as “the mahogany notcher” (Morton 1987: 191) is wood-boring as a larva and as an adult feeds on foliage of mahogany (Meliaceae Juss.: *Swietenia mahogany* (L.) Jacq.) (Morton 1987). Other species are known from avocado (Lauraceae: *Persea*) (Kissinger 1957: 7, Muñiz V. 1965), *Bursera citronella* McVaugh & Rzed. (Burseraceae Kunth) (Muñiz-Vélez and Ordóñez-Reséndiz 2010), and *Cecropia*, *Coussapoa*, and *Pourouma* (Urticaceae) (LaPierre 2002).

#### Described species.

Fifty-six species are known from the focal region, including one described by Hespenheide (1984), three by Zayas (1988), one by Muñiz-Vélez and Ordóñez-Reséndiz 2010, and one by Hespenheide (2017), and one species transferred from *Archocopturus* by Hespenheide (2005). An additional 101 species are known from South America (Wibmer and O’Brien 1986: 272, with two additional species described by Rheinheimer (2011)).

#### Range.

USA: FL, Mexico, Guatemala, Belize, Honduras, Nicaragua, Costa Rica, Panama, Bahamas, Cuba (Zayas 1988), Jamaica, Puerto Rico, Guadeloupe (Hespenheide 1984); South America.

### Macrolechriops

Taxon classificationAnimaliaColeopteraCurculionidae

Champion, 1906b: 126

Fig. 84

= Parazurus Hustache, 1937: 108 [Syn.: Rheinheimer 2011: 77]. Type species: Parazurus nodieri Hustache, 1937 [by original designation].

#### Type species.

*Macrolechriops spinicoxis* Champion, 1906 [by monotypy].

#### Gender.

Masculine.

#### Diagnosis.

*Macrolechriops* belongs in the *Eulechriops* complex of genera with its short second funicular article, non-carinate and unarmed hind femora, and carinate and excavated mesoventrite, but can be distinguished (at least the Central American species) by the hump-like pronotal carina.

#### Notes.

Champion reported the presence of a “...flattened, conical prominence on the intermediate, as well as on the anterior, coxae...” (1906b: 127) as being unique among the conoderines he examined. This character, however, has been observed in other species and genera, including some *Eulechriops*, reducing the diagnostic utility of that character among the putative relatives of *Macrolechriops*.

The specimen in Fig. 84 agrees with Champion’s description, which was based on “one worn specimen” (Champion 1906b: 127), but direct comparison of the specimen with the holotype is needed to confirm the identity as no other identified material of that species has been observed in the course of this study.

The South American species of *Macrolechriops* described by Hustache have a pronotum that is strongly convex but without a hump-like carina. This hump-like pronotal carina is known from other genera, such as *Macrocopturus verrucosus* (Champion, 1906), but none described or so far known in the genus *Eulechriops* or genera closely related to it, making it a useful character for separating the only currently known Central American species of *Macrolechriops* from its relatives. A very similar vestiture pattern and pronotal shape has been observed in other genera, most notably in a species of *Copturomimus* which has been seen in several collections incorrectly identified as *Macrolechriops spinicoxis*.

#### Phylogenetic relationships.

Champion (1906b: 126) notes the similarity with the South American *Machaerocnemis* Heller, 1895 and *Copturosomus*, but the genus is here considered part of the *Eulechriops* genus complex.

#### Host association

**s.** Unknown.

#### Described species.

One species is known from the focal region and five additional species are known from South America (Wibmer and O’Brien 1986: 266, Rheinheimer 2011).

#### Range.

Mexico, Honduras [ARTSYS0000529]; South America.

### Microzurus

Taxon classificationAnimaliaColeopteraCurculionidae

Heller, 1895: 13

Figs 58 , 85

#### Type species.

*Microzurus rhombus* Heller, 1895 [by monotypy].

#### Gender.

Masculine.

#### Diagnosis.

*Microzurus* can be differentiated from *Copturus* by the lack of a ventral tooth on the profemora, a thin fifth tarsomere with minute tarsal claws (Fig. 58), and costate elytral intervals. Champion (1906: 89) described two species that have shallow or absent modification to the mesoventrite – no material was observed of the species without modification (*M. edentatus* Champion, 1906), but the species would still be easily recognized as a *Microzurus* by the concealed scutellum, minute tarsal claws, and absent ventral tooth on the pro- and mesofemora. The second funicular article is not longer than the first and the hind femora are carinate and ventrally toothed. The observed species have a similarly apically laterally flanged receptacle of the mesoventrite as in *Copturus*, though it is usually much less prominent.

#### Phylogenetic relationships.

Hespenheide (1984) suggested a relationship between *Microzurus* and *Cylindrocopturinus*. *Microzurus* is here interpreted as closely related to *Copturus*, but the position of those two genera within the lechriopines is uncertain. See entry on *Copturus*.

#### Host associations.

Hespenheide (1984: 316) reported the possibility of seed-feeding based on label data. Costa-Lima (1956: 219) mentions South American species on fruits of *Campomanesia* Ruiz & Pav. and *Psidium guajava* (Myrtaceae).

#### Described species.

Three species are known from the focal region and an additional four species are known from South America (Wibmer and O’Brien 1986: 266).

#### Range.

Mexico, Belize, El Salvador, Honduras, Panama; South America.

### Microzygops

Taxon classificationAnimaliaColeopteraCurculionidae

Champion, 1906b: 46

Figs 11 , 45 , 86

#### Type species.

*Microzygops nigrofasciatus* Champion, 1906 [by original designation].

#### Gender.

Masculine.

#### Diagnosis.

*Microzygops* can be distinguished from other lechriopine genera with an elongate second funicular article and modification to the mesoventrite by the following characters: the shape of the eyes, which are very large and contiguous in bottom 2/3 and widely separated in top 1/3 (Fig. 45), the distinct form of the mesoventrite (Fig. 11) which is similar only to the few species of *Macrocopturus* that have modification to the mesoventrite with an elevated posterior margin of the mesoventrite and faint, arcuate longitudinal carinae (as well as a deeply excavated anterior margin of the metaventrite), and the color pattern, which is putatively ant mimetic (Hespenheide 1995) but distinct from the other ant mimics with orange-brown ground color and transverse black fascia of the pronotum and elytra.

The metafemora are very elongate, extending well past the abdominal apex and lacking carina. The pro- and mesofemora are ventrally toothed, but the metafemur is unarmed in *Microzygops nigrofasciatus* though with a small tooth in the South American *M. flavatus* Rheinheimer, 2011 and one undescribed Central American species [SSAC0001210]. Other generic characters given by Champion (1906b: 46) include the “exserted head” and “cylindrical constricted prothorax”.

#### Notes.

The species *M. nigrofasciatus* like the species of several other genera originally described as monotypic, is possibly a complex of several species – specimens identified to that species have been observed from Mexico to Peru and at a range of elevations.

#### Phylogenetic relationships.

The exserted head, elongate and slender hind legs, proportionately short and narrow third tarsomere, and linear carina of the vertex of the head, in combination, is only similar to *Pseudolechriops* and, to a lesser extent (excluding the head characters) *Lissoderes*, but the mesoventrite is distinct in each of those genera. *Microzygops* was among the genera moved from the Zygopini to the Lechriopini in Lyal et al. (2006), but the position of the genus within the Lechriopini is at present uncertain.

#### Host associations.

Unknown.

#### Described species.

One species is known from the focal region and one additional species is known from French Guiana (Rheinheimer 2011: 68).

#### Range.

Mexico [ASUHIC0031512], Costa Rica [ASUHIC0086639], Panama; South America.

### Mnemynurus

Taxon classificationAnimaliaColeopteraCurculionidae

Heller, 1895: 54

Fig. 87

#### Type species.

*Mnemynurus caloderes* Heller, 1895 [by monotypy].

#### Gender.

Masculine.

**Figures 87–90.**
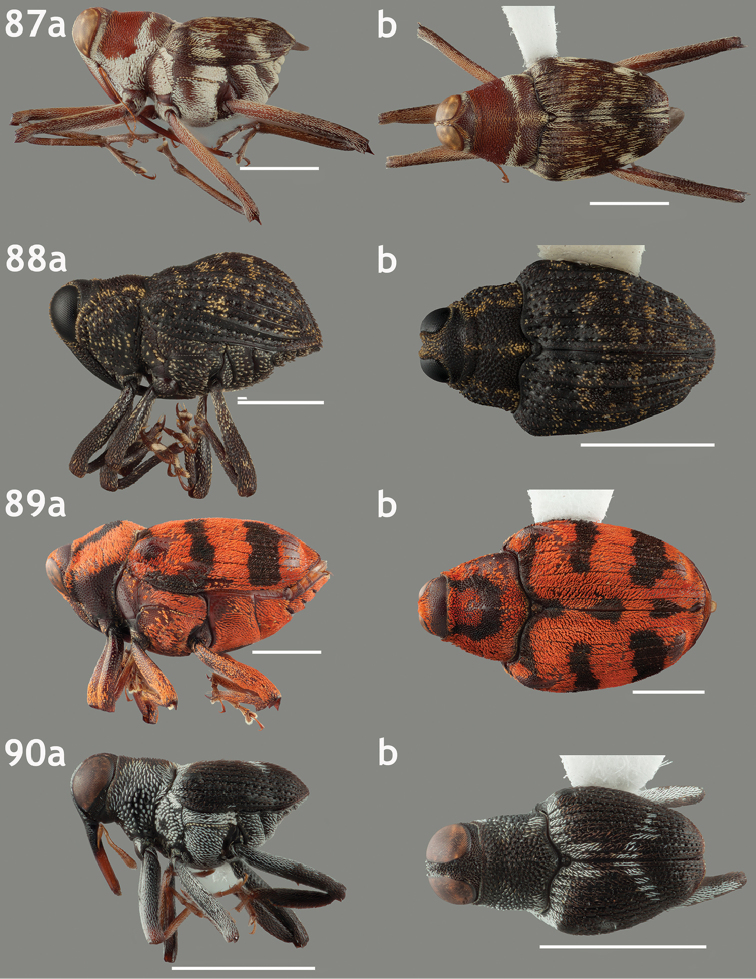
Lateral and dorsal habitus images of Lechriopini. **87a–b** *Mnemynurus poeciloderes* [ARTSYS0000803] **88a–b** *Paramnemyne decemcostata* [ASUHIC0065104] **89a–b** *Poecilogaster brevis* [ASUHIC0086631] **90a–b** *Pseudolechriops megacephalus* [ASUHIC0086629]. Scale bars = 2 mm.

#### Diagnosis.

All of the described and most of the observed undescribed species of *Mnemynurus* are members of the red-headed fly mimicry complex (Hespenheide 1973, 1995), and are most difficult to distinguish from the similarly patterned species of *Hoplocopturus* and the South American *Balaninurus* which also share the distinctive inverted U-shaped carina on the mesoventrite. See “Diagnosis” for *Hoplocopturus* above for more information on the separation of the genera. The second funicular article is much longer than the first, the apex of rostrum is flattened and dilated, the hind femora are ventrally toothed and sometimes carinate, and the rostrum usually extends past the posterior border of metaventrite (though not in *M. longispinis*) and sometimes beyond the apex of the abdomen.

#### Notes.

*Mnemynurus*, *Paramnemyne*, and the South American genus *Mnemyne* are the only New World genera having a rostral channel extending at least to the posterior half of the metaventrite. A distinct channel is found only in the larger *Mnemynurus* species and the metaventrites of other species are longitudinally depressed. Zayas (1988) described two species from Cuba, although based on the descriptions their placement in this genus is uncertain.

#### Keys.

Champion 1906b: 49 (for Central America; *M. caloderes* in key = *M. championi* Heller, 1933 (Heller 1933: 150)), Heller 1932a: 5 (only to the three species described by Heller).

#### Phylogenetic relationships.

Most similar to the South American genus *Balaninurus* Heller, but generic limits between these two genera and *Hoplocopturus* need resolving – see *Hoplocopturus* above.

#### Host associations.

Species of *Mnemynurus* have been recorded as gall-inducing on young leaves of *Philodendron* Schott (Araceae) (Hanson et al. 2014: 503).

#### Described species.

Nine species are known from the focal region including two described by Zayas (1988) and two additional species are known from South America (Wibmer and O’Brien 1986: 270).

#### Range.

Mexico, Honduras, Nicaragua, Costa Rica, Panama, Cuba [Zayas 1988]; South America.

### Paramnemyne

Taxon classificationAnimaliaColeopteraCurculionidae

Heller, 1895: 10

Figs 12 , 88

#### Type species.

*Paramnemyne arcana* Heller, 1895 [by subsequent designation: Rheinheimer 2011: 78].

#### Gender.

Feminine.

#### Diagnosis.

*Paramnemyne* can be readily distinguished by the transverse carina near the posterior margin of the metaventrite, marking the end of the rostral channel (Fig. 12). The second antennal funicular article is several times longer than the first; the femora are not carinate, are ventrally toothed, and are unarmed at the apices; the mesopleura are small and non-ascending; and the eyes are completely separated with the greatest separation in the middle.

#### Phylogenetic relationships.

The unarmed femoral apices, small and non-ascending mesopleura, and piazurine type of mesoventrite suggest improper placement in the Lechriopini, however transferring the genus without observing the putative relatives of *Paramnemyne*, the South American genera *Mnemyne* (*sec.* Heller 1895: 11) and *Paramnemynellus* Hustache, 1932 (*sec.* Hustache 1932b: 207), would be remiss.

#### Host associations.

Unknown.

#### Described species.

Two species are known from the focal region and three additional species are known only from South America (Wibmer and O’Brien 1986: 270).

#### Range.

Mexico, Guatemala, Costa Rica [O’Brien and Wibmer 1984: 296], Panama; South America.

### Poecilogaster

Taxon classificationAnimaliaColeopteraCurculionidae

Heller, 1895: 16

Figs 46 , 89

#### Type species.

*Poecilogaster longior* Heller, 1895 [by subsequent designation: Alonso-Zarazaga and Lyal 1999: 115] (=*Copturus brevis* Waterhouse, 1879).

#### Gender.

Feminine.

#### Diagnosis.

*Poecilogaster* has the general appearance of a large *Lechriops* that lacks a femoral carina. The rostral channel extends to the anteriorly depressed metaventrite and is laterally carinate on the mesoventrite, the second antennal funicular article is slightly longer than the first, the metafemora are not carinate and toothed ventrally, and the mesal face of femoral apex usually bears a long spine. Champion (1906: 44) notes a large tubercle on the prosternum behind the procoxae, which is not unique to *Poecilogaster* and known in other genera (e.g. some *Copturomorpha*).

#### Phylogenetic relationships.

While easily recognized by general appearance it is difficult to place within the Lechriopini. The longer second funicular article, ventrally toothed femora, and long spine at the mesal face of the femoral apices are suggestive of a relationship with *Lechriops* and *Hoplocopturus* while the non-carinate femora and deeply excavated mesoventrite is similar to *Eulechriops*. Heller (1895: 16) suggests a relationship with *Lechriops* and also speculates a potential relationship with the South American genera *Hemigaster* Lacordaire, 1865 (= *Hemicolpus* Heller, 1895) and *Acopturus* Heller, 1895 based on the shape of the second abdominal ventrite. Neither of those latter genera have been observed in the course of this study but both are currently placed in the Zygopini.

#### Host associations.

Unknown.

#### Described species.

Two, including one described by Zayas (1988).

#### Range.

Costa Rica, Panama, Cuba (Zayas 1988); South America.

### Pseudolechriops

Taxon classificationAnimaliaColeopteraCurculionidae

Champion, 1906b: 90

Figs 13 , 26 , 90

#### Type species.

*Pseudolechriops megacephalus* Champion, 1906 [by original designation].

#### Gender.

Masculine.

#### Diagnosis.

*Pseudolechriops* is rather distinctive in appearance yet difficult to satisfactorily characterize as a genus, with variation across the species in the following characters: the insertion of the antenna on the rostrum can be in the basal (e.g. in *P. megacephalus* Champion, 1906) or apical half (e.g. *P. klopferi* Hespenheide & LaPierre, 2006), the second funicular article can be longer than (e.g. in *P. megacephalus*) or subequal to the length of the first article (e.g. *P. coleyae* Hespenheide & LaPierre, 2006), the eyes can be vertical and relatively widely separated (e.g. in *P. megacephalus*) or larger and subcontiguous (e.g. in *P. klopferi*), the inner flange of the tibial apex can be flat (with no projection) to bearing an elongate, uncus-like process (Fig. 26), and hind femora that can be completely carinate and ventrally toothed (e.g. *P. megacephalus*), or partially carinate basally and without a tooth (e.g. *P. coleyae*). Despite this variation, the modification to the mesoventrite is unique, with the rostral channel being a deep, ovoid, receptacle (Fig. 13) for receiving the rostrum on the mesoventrite and anterior margin of metaventrite. Additionally, the procoxae lack a mesal process, which is found in many other lechriopines.

#### Notes.

Hespenheide and LaPierre (2006) distinguish two distinct species groups. The species are possibly mimics of ants in the genus *Azteca* Forel, 1878 (Hespenheide and LaPierre 2006: 37).

#### Phylogenetic relationships.

Champion (1906: 90) and Lyal et al. (2006: 229) noted similarities with the South American genus *Tachylechriops* Heller, 1895 and *Lechriops*; Hespenheide and LaPierre (2006: 3) disagree but do not present an alternative hypothesis. The mesoventrite of *Pseudolechriops* is most similar to that of *Lechriops* in shape although the sides of the channel in *Pseudolechriops* are much more ventrally prominent and the median channel deeper. The exserted head, elongate hind femora, and vertex of head with a linear carina are similar to *Microzygops*.

#### Host associations.

Adults can be found on the undersides of leaves of several species of *Cecropia* (Urticaceae), and the larvae develop in living or dead leaf petioles (Jordal and Kirkendall 1998, LaPierre 2002, Hespenheide and LaPierre 2006).

#### Described species.

Ten, including nine described by Hespenheide and LaPierre (2006).

#### Range.

Mexico [Hespenheide and LaPierre 2006], Guatemala, Belize, Honduras [Hespenheide and LaPierre 2006], Nicaragua [Hespenheide and LaPierre 2006], Costa Rica [Hespenheide and LaPierre 2006], Panama; South America [Hespenheide and LaPierre 2006].

### Psomus

Taxon classificationAnimaliaColeopteraCurculionidae

Casey, 1892: 458

Fig. 91

#### Type species.

*Psomus politus* Casey, 1892 [by monotypy] (=*Orchestes armatus* Dietz, 1891).

#### Gender.

Masculine.

**Figures 91–94.**
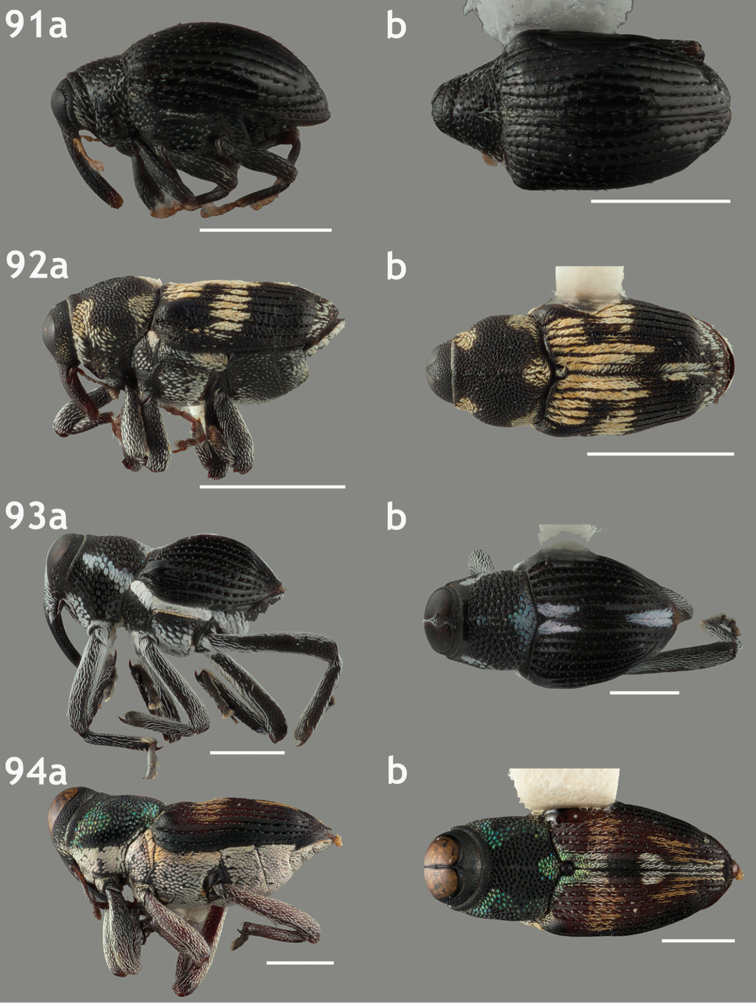
Lateral and dorsal habitus images of Lechriopini and Zygopini. All scale bars = 1 mm unless otherwise specified. **91a–b** *Psomus armatus* [ARTSYS0000533] **92a–b** *Turcopus viscivorus* [ARTSYS000530]; scale bar = 2 mm **93a–b** *Arachnomorpha circumlineata* [ARTSYS0000535] **94a–b** *Archocopturus laselvaensis* [ASUHIC0086633].

#### Diagnosis.

The combination of concealed pygidium, appendiculate tarsal claws, and sulcate subapical pronotal constriction readily distinguishes *Psomus* from the other genera treated here. *Philides* is the only other genus without simple tarsal claws and *Peltophorus* and *Zygops* have a sulcate subapical pronotal constriction, but each of those three genera have an exposed pygidium (which *Psomus* does not) and are otherwise distinct in habitus. *Psomus* is unique among the lechriopine genera for having a flattened mesoventrite and Type II sclerolepidia (Type II sclerolepidia also known from *Copturus*, *Microzurus*, and *Euzurus*, though each has a modified mesoventrite) although this combination is also found in *Lissoderes*, which is currently a zygopine (Lyal et al. 2006: 229); neither of those genera seem very well placed in their current tribes.

Some observed species have a ventrally expanded first abdominal ventrite and modifications to the profemora and tibiae that are similar to what is found in some Cleogonini (Prena and Whitehead 2012: 57). These differences were not mentioned by Champion when describing three Central American species, but he considered his species as “perfectly congeneric with *P. politus*, Casey [=*P. armatus* (Dietz)]” (Champion 1906b: 128). In addition to the characters given above distinguishing the genus, the species of *Psomus* have a second funicular article that is about equal to the first and a tibial apex with an uncus at the posterior apical angle or the middle of the apex.

#### Phylogenetic relationships.

The appendiculate tarsal claws (shared only with *Philides*) and a deep subapical pronotal constriction (shared only with *Zygops* and *Peltophorus*) are easily observed characters but not particularly suggestive of a relationship. The single U.S. species, *P. armatus* (Dietz, 1891) was originally described in the genus *Orchestes* Illiger, 1798 (Curculioninae: Rhamphini). *Psomus* bears a resemblance to the cleogonine *Isotrachelus* (which was previously placed in the Old World conoderine tribe Lobotrachelini Lacordaire, 1865), but differs from *Isotrachelus* by the insertion of the antenna on the rostrum, which in *Psomus* is in the basal half of the rostrum, and the tarsal claws, which in *Isotrachelus* are simple.

#### Host associations.

*Psomus armatus* can be found on ash trees (Oleaceae Hoffmanns. & Link: *Fraxinus* L.) (Sleeper 1963). Hosts of the Central American species are unknown.

#### Described species.

Four.

#### Range.

Eastern Canada and U.S.A., Guatemala, Panama.

### Turcopus

Taxon classificationAnimaliaColeopteraCurculionidae

R.S. Anderson, 1994: 475

Figs 14 , 92

#### Type species.

*Turcopus viscivorus* R.S. Anderson, 1994 [original designation].

#### Gender.

Masculine.

#### Diagnosis.

R.S. Anderson (1994: 463) separates *Turcopus* from *Coturpus*, *Cylindrocopturinus*, and *Eulechriops minutus* (LeConte, 1824) by the deep, prominently carinate, cup-like receptacle on the mesoventrite (Fig. 14); this has not been seen in the numerous observed specimens of *Eulechriops*, representing mostly undescribed species, but is difficult to generalize the mesoventrite for all species of such a variable genus. *Turcopus* is otherwise difficult to distinguish from *Eulechriops* except by the vestiture pattern (Fig. 92) and host association, which are currently unknown in *Eulechriops*. *Turcopus* can be further separated from *Coturpus* by genitalic characters given by R.S. Anderson (1994: 477).

#### Phylogenetic relationships.

R.S. Anderson (1994: 477) proposed a relationship with the sister taxa of *Coturpus* + *Cylindrocopturinus* (those three taxa are the proposed sister to *Eulechriops*). The difficulty of separation with *Eulechriops* suggests a closer relationship with that genus but much work needs to be done in delimiting generic boundaries in this complex of genera.

#### Host associations.

*Turcopus* has been collected on *Phoradendron* on *Quercus* (R.S. Anderson 1994: 479).

#### Described species.

One (R.S. Anderson 1994).

#### Range.

Mexico, Guatemala.

### Zygopini

Taxon classificationAnimaliaColeopteraCurculionidae

Lacordaire, 1865: 150

#### Classificatory history and current circumscription.

This tribe was originally characterized by Lacordaire (1865: 150) for the genera *Zygops*, *Peltophorus*, *Copturus*, *Timorus* and *Hemigaster* (=*Hemicolpus*) by a more-or-less canaliculate prosternum, a flat, unmodified mesoventrite, and straight, carinate hind femora that can exceed the apex of the abdomen. Presently, the genera placed in Zygopini lack sclerolepidia (except for *Arachnomorpha*, *Lissoderes*, and some species of *Philenis*) and lack modification to the mesoventrite (except *Peltophorus* and most species of *Philenis*). Davis and Engel (2006) also suggested the “strongly protuberant compound eyes, deeply depressed pronotal lateral-facing surfaces, and relatively large genae”, but these features are also shared with several lechriopine genera.

As indicated previously, of the genera currently placed in the Zygopini from the focal region, *Zygops* and *Peltophorus* are quite distinct from the rest with a large, exposed pygidium that is at least mostly visible in dorsal view and abdominal ventrites that do not ascend rapidly, a fifth abdominal ventrite that is arcuate in lateral profile (deflected apically downwards by the large pygidium). Additionally, most observed specimens of these genera have a quadrate to transversely rectangular scutellum (visible in Fig. 102b). The Dominican and Mexican amber fossil genus *Geratozygops* appears to belong to this group of “true zygopines”, and as best could be determined from the images provided by Davis and Engel (2006) and Poinar and Legalov (2013) the species would key out to *Zygops* in the above key. *Latychus*, the South American piazurine proposed by Prena et al. (2014: 300) to be the identity of *Geratozygops*, would run to couplets 7-10, which treats the Piazurini.

The genera besides *Zygops* and *Peltophorus* can be identified by having the following combination of characters: a concealed pygidium, strongly ascending abdominal ventrites, and a second funicular article that is subequal to or shorter than article 1 (except *Philenis* and some *Cylindrocopturus*).

#### Variation in key character systems.

The mesoventrite of most genera and species is unmodified, with exceptions being found in *Peltophorus* (which has the mesoventrite ventrally produced and nearly cup-like, Fig. 16), a few species of *Zygops* (with the posterolateral margins tumescent or with small processes), most species of *Philenis* (with a posteromedial semicircular depression and posterolateral tubercles, Fig. 17), and a few other species with slight posteromedial depressions (e.g. *Archocopturus championi* Hespenheide, 2005). The general form of the tibial apex varies little from the typical conoderine form apart from having a very short and curved uncus (in *Helleriella* Champion, 1906 and *Peltophorus*, Figs 30 and 33, respectively), a minute premucro (in *Lissoderes* and *Arachnomorpha*, Figs 27 and 31, respectively), and a premucro oriented at a 45° angle to the longitudinal axis of the tibia (in *Phileas* Champion, 1906 and *Philenis*, Figs 32 and 35, respectively).

#### Diversity and distribution.

Eighty-three species are currently known from the 11 genera occuring north of South America. An additional 8 genera occur exclusively in South America and two more are also recorded from Africa.

### Arachnomorpha

Taxon classificationAnimaliaColeopteraCurculionidae

Champion, 1906b: 47

Figs 27 , 47 , 93

#### Type species.

*Arachnomorpha circumlineata* Champion, 1906 [by original designation].

#### Gender.

Feminine.

#### Diagnosis.

*Arachnomorpha* can be distinguished from the zygopine genera with a flattened mesoventrite, concealed pygidium and short second funicular article by the broad interocular space between the top of the eyes (Fig. 47), with the eyes closer together below the space than above, the costate elytral intervals, the carinate and ventrally unarmed hind femora and minute premucro (Fig. 27). Additionally, *Arachnomorpha circumlineata* is part of a “shiny-black” ant-mimicry complex (Hespenheide 1995), with the cuticle in large part glabrous and black with patches or stripes of white or opalescent scales. This mimicry complex, among the New World Conoderinae, is so far known only in *Arachnomorpha*, *Microzurus*, *Lissoderes* and *Philides*. The observed undescribed *Microzurus* species [SSAC0001290] also has sharply costate elytral intervals but can be easily distinguished from *Arachnomorpha* by the concealed scutellum and modified mesoventrite.

#### Phylogenetic relationships.

Champion (1906b: 47) posits a relationship with the South American *Mnemyne* and Hespenheide (1987: 42) notes the similarity with *Lissoderes*. Of the zygopine genera with a concealed pygidium and a short second funicular article, only *Arachnomorpha* and *Lissoderes* have sclerolepidia, although apparently of a different type (Lyal et al. 2006: 229). *Arachnomorpha* and *Lissoderes* additionally have a minute premucro of the tibial apex, but differently shaped eyes, antennal insertion on different parts of the rostrum (basal third in *Arachnomorpha*), and a different body shape. The genera of Zygopini that have a concealed pygidium and a second funicular article that is not longer than the first (*Arachnomorpha*, *Archocopturus* Heller, 1895, most *Cylindrocopturus*, *Helleriella*, *Larides* Champion, 1906, *Lissoderes*, *Phileas*, and *Zygopsella* Champion, 1906), with the exception of *Cylindrocopturus*, are all small genera (five described species or less) that are very distinct in body shape and/or coloration likely owing to their participation in different mimicry complexes, and are otherwise difficult to separate by external characters. Of those genera, large pronotal punctures are also shared with *Archocopturus* and *Zygopsella*.

#### Host associations.

Unknown.

#### Described species.

One.

#### Range.

Costa Rica, Panama.

### Archocopturus

Taxon classificationAnimaliaColeopteraCurculionidae

Heller, 1895: 56

Figs 48 , 94

#### Type species.

*Copturus regalis* Boheman, 1845 [by monotypy].

#### Gender.

Masculine.

#### Diagnosis.

**Figures 95–98.**
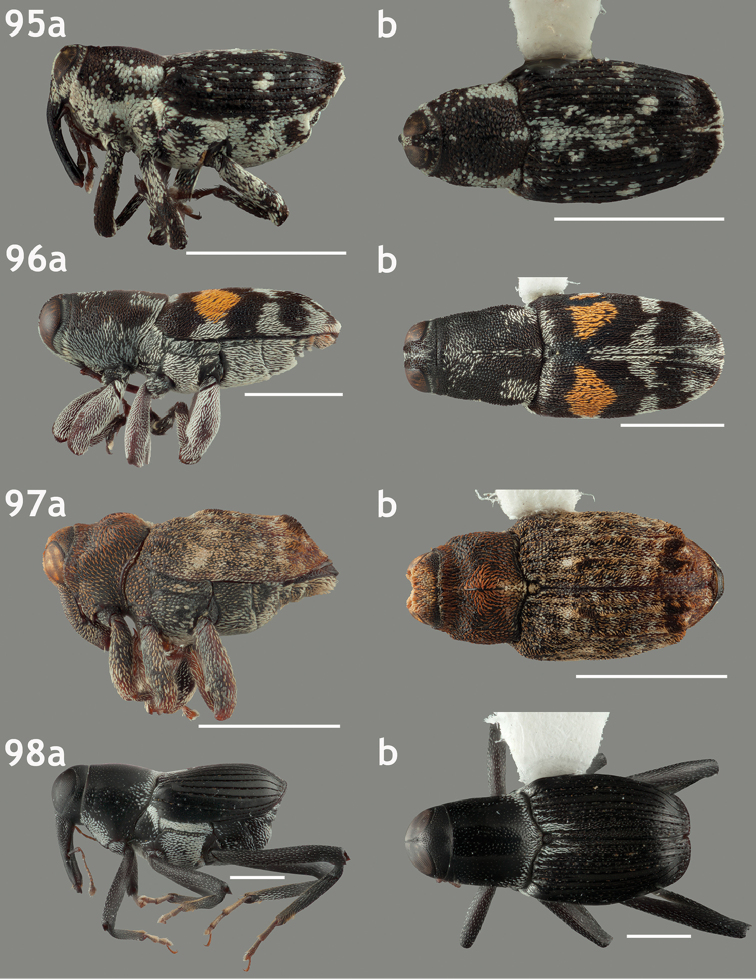
Lateral and dorsal habitus images of Zygopini. Scale bars = 2 mm unless otherwise specified. **95a–b** *Cylindrocopturus quercus* [ARTSYS0000819]; scale bars = 1 mm **96a–b** *Helleriella longicollis* [ASUHIC0065241] **97a–b** *Larides cavifrons* [ASUHIC0016882] **98a–b** *Lissoderes subnudus* [SSAC0001136]; scale bars = 1 mm.

*Archocopturus* can be separated from the other zygopines that have a concealed pygidium and a second funicular article that is subequal to the first by the following combination of characters: the eyes are separated at the top by a small lanceolate space (Fig. 48; also in other genera – e.g. many species of *Macrocopturus*), the vertex of head has a triangular, transversely striolate region (visible in Figs 48 and 94b for *Archocopturus* but most noticeable in Fig. 95b for *Cylindrocopturus*; also seen in some species of other genera, e.g. *Zygops*, *Cylindrocopturus*) the pronotum has deep, close punctures, the profemora are unarmed, and the hind femora are carinate and ventrally toothed and do not extend much beyond the abdominal apex. Additionally, all known species of *Archocopturus* have blue-green scales on the pronotum, suggesting mimicry of the dolichopodid genus *Medetera* (Hespenheide 2005). While this coloration is found in several other genera of Conoderinae, the only other zygopine with it is *Zygopsella*, which *Archocopturus* can be easily separated from by the lack of a ventral profemoral tooth and the more approximate eyes. The mesoventrite is flat in most species but posteromedially depressed in *A. championi*.

#### Keys.

Hespenheide 2005: 673.

#### Phylogenetic relationships.

Champion (1906b: 42) suggests a relationship with *Zygopsella*. The two genera have in common the deep punctures of the pronotum (also in *Arachnomorpha*) and blue-green scales.

#### Host associations.

The South American *Archocopturus regalis* (Boheman, 1845) has been reared from branches of Lecythidaceae in Peru (Fassbender 2013).

#### Described species.

Four species are known from the focal region, which includes all four species described by Hespenheide (2005). One additional species is known from South America (Wibmer and O’Brien 1986: 270, Hespenheide 2005: 671).

#### Range.

Mexico, Belize, Guatemala, Honduras (Hespenheide 2005), Nicaragua, Costa Rica, Panama; South America.

### Cylindrocopturus

Taxon classificationAnimaliaColeopteraCurculionidae

Heller, 1895: 56

Figs 29 , 49 , 95

= Paratimorus Heller, 1895: 58 [Syn.: Champion 1906b: 35]. Type species: Paratimorus ganglbaueri Heller, 1895 [by monotypy].
= Gyrotus Casey, 1897: 668 [Syn.: Sleeper 1963: 217]. Type species: Gyrotus munitus Casey, 1897 [by monotypy].
= Copturodes Casey, 1897: 669 [Syn.: Casey 1904: 324]. Type species: Zygops quercus Say, 1831 [by subsequent designation: Sleeper 1963: 217].

#### Type species.

*Zygops quercus* Say, 1831 [by subsequent designation: Sleeper 1963: 217].

#### Gender.

Masculine.

#### Diagnosis.

Many of the species of *Cylindrocopturus* can be distinguished by the following combination of characters: the unmodified mesoventrite, the unarmed and non-carinate metafemora, the second antennal funicular article that is not longer than the first, the relatively vertical and separated eyes (Fig. 49), and the body mostly densely covered in round, imbricate scales. Additionally, the body is often somewhat dorsoventrally compressed, the elytra sometimes has prominences or setal tufts, the tibial apex at least of the protibia typically has a large hook-like uncus and a rounded, produced inner flange (Fig. 19), and a group of species (*C. mammillatus* species group of Fall (1906), *Gyrotus* Casey of Gluck (1987)) have a pair of tubercles on the second abdominal ventrite. These characters are useful for separation of the species found in the U.S.; of the several observed Mexican, Guatemalan, and Honduran species, including numerous unidentified and likely undescribed, the following exceptions to the above characters have been observed: a depressed posterior border of the mesoventrite, a second funicular article that is longer than the first, and carinate hind femora. These species otherwise appear congeneric with described species, highlighting the need for closer examination and reconstruction of this genus and its relatives.

#### Notes.

*Cylindrocopturus* is in need of comprehensive revision due to the large number of synonymies, uncertain geographic range and lack of good characters separating it from several other genera. An unpublished Ph.D. thesis by W. Gluck (1987) attempted such for the species north of Mexico, but the heavily-relied upon statistical approach employed for generic and specific delimitation necessitates closer examination of many of the classificatory changes proposed.

Immature stages are described for the following species: *C. adspersus* (LeConte, 1876) by Böving (1926), *C. biradiatus* Champion, 1906 by Dampf (1929), *C. crassus* Van Dyke, 1930 by Keifer (1930), *C. furnissi* Buchanan, 1940 by W.H. Anderson (1941), *C. quercus* by Piper (1977). See Gluck (1987: 78) for an index of the seventeen known species of hymenopteran parasites of the species of *Cylindrocopturus*.

#### Keys.

Champion 1906: 36 (to Central American species), Fall 1906: 55 (to *C. mammilatus* species group), LeConte and Horn 1876: 261 (to *Copturus* of the U.S.), Casey 1897: 669 (to *Copturodes* of the U.S.), Blatchley and Leng 1916: 420 (to Northeastern U.S. species), Hatch 1971: 361 (to *Gyrotus* of Northwestern U.S.), Heller 1895: 57, Gluck 1987: 9 (to *Gyrotus* north of Mexico) and Gluck 1987: 30 (to *Cylindrocopturus* north of Mexico).

#### Phylogenetic relationships.

Hespenheide (1980: 330) suggests the genus *Cylindrocopturus* as the closest relative of *Helleriella* due to the shared elongate and compressed habitus and occurrence in arid environments. Champion distinguishes *Cylindrocopturus* from the South American genus *Timorus* by the lack of a ventral femoral tooth, and seems to imply possible relationships between *Timorus*, *Cylindrocopturus*, *Macrocopturus*, *Phileas*, and *Larides* (1906: 33-35). See *Macrocopturus*.

#### Host associations.

Species of *Cylindrocopturus* have been reared from various species of Pinaceae (in the genera *Abies* Mill., *Pinus*, and *Pseudostuga* Carrière), various Asteraceae Bercht. & J. Presl (e.g. *Helianthus* L., *Hemizonia* DC.) and also Cactaceae Juss. (*Opuntia* Mill.); larvae of some species known from roots, stems, branches, galls and spines (Casey 1897, Fall 1906, Blatchley and Leng 1916, Dampf 1929, Van Dyke 1930, Buchanan 1940, Gluck 1987: 77, Martínez et al. 2016). The “sunflower stem weevil”, *C. adspersus*, is the most well-studied species of New World conoderine due to its agricultural importance in the Midwestern United States, with studies including insecticide toxicity (e.g. Charlet and Oseto 1983) and overwintering and emergence patterns (Rogers and Serda 1982).

#### Described species.

Forty-one species are known from the focal region and two additional described species are known from South America (Wibmer and O’Brien 1986: 270), though Rheinheimer (2011: 78) suggests *Eulechriops* as a better placement for the French Guianan *C. minutus* Hustache, 1938.

#### Range.

Canada, USA, Mexico, Guatemala, Honduras; South America.

### Helleriella

Taxon classificationAnimaliaColeopteraCurculionidae

Champion, 1906b: 32

Figs 30 , 50 , 96

#### Type species.

*Helleriella longicollis* Champion, 1906 [by monotypy].

#### Gender.

Feminine.

#### Diagnosis.

The slender rostrum (Fig. 50), elongate pronotum, linear scales, and a very short tibial uncus (Fig. 30) separates *Helleriella* from the zygopine genera with a concealed pygidium, flattened mesoventrite and second funicular article that is not longer than the first. The eyes are somewhat widely separated, especially near the top, strongly inflexed along outer margin towards bottom where it is sharply acuminate, the femora are non-carinate, with or without a ventral tooth, and are short and thick in some species.

#### Notes.

The species of *Helleriella* have been suggested to belong to different mimicry complexes, including clytrine chrysomelids, *Zacryptocerus* ants, and possibly red-eyed flies and other species of ants (Hespenheide 1980).

#### Phylogenetic relationships.

Hespenheide (1980: 330) suggests a relationship with *Cylindrocopturus* due to the “...elongate, compressed habitus... the pronotum distinctly narrower than the elytra, and an investiture of scales that are predominantly linear and only overlap end-to-end in contrast to broad, completely overlapping, encrusting scales of most *Cylindrocopturus*.”

#### Keys.

Hespenheide 1980: 329 and 1998: 3.

#### Host associations.

Associated with several species of “swollen thorn *Acacia*” (Fabaceae: Mimosoideae DC.) (Hespenheide 1980). Larvae live and feed in thorns not occupied by ants (Hespenheide 1980).

#### Described species.

Five species are known, including one described by Hespenheide (1998).

#### Range.

Mexico, Guatemala, El Salvador (Hespenheide 1980: 325), Belize, Nicaragua, Costa Rica.

### Larides

Taxon classificationAnimaliaColeopteraCurculionidae

Champion, 1906b: 34

Figs 51 , 97

#### Type species.

*Larides cavifrons* Champion, 1906 [by original designation].

#### Gender.

Masculine.

#### Diagnosis.

*Larides* is distinct from all other zygopine genera treated here with the exception of *Phileas* with the short, stout, arcuate rostrum that does not extend much beyond the procoxae, more strongly developed ocular lobes that partially cover the eye, and eyes widely separated at the top and strongly concave in between (Fig. 51). The antennae are inserted near the middle of the rostrum, the second antennal funicular article is not longer than the first, the mesoventrite is unmodified, and the hind femora are ventrally toothed and faintly carinate in the distal half. The distinction given by Champion (1906b: 35) between *Larides* and *Phileas* in their original descriptions is that *Larides* has the eyes “less acuminate below and more widely separated above, the antennal club shorter and relatively stouter, and the prothorax and elytra subtruncate at the base” seem insufficient for generic distinction, especially when considering the intrageneric variation of those characters in other conoderine genera. Both *Larides* and *Phileas* are monotypic, but *Larides cavifrons* can be easily separated from *Phileas granulatus* Champion by the more strongly depressed interocular space and the metatibial apex that has a premucro oriented along the longitudinal axis of the tibia (at a 45° angle in *Phileas granulatus*).

#### Notes.

Couplet 38 in the below key serves to distinguish the genera *Larides* and *Phileas*, however, few specimens of *Larides* and only one of *Phileas* were observed in this study. Whether the tibial apex character, which easily separates the observed specimens but was not mentioned by Champion in the original descriptions, will hold for generic distinction when additional specimens and species are observed remains to be seen.

#### Phylogenetic relationships.

Very similar to *Phileas*, and as noted by R.S. Anderson (1994: 486) they are possibly congeneric, but insufficient material has been observed to comment further. Both genera share with the South American *Timorus* the short, robust rostrum, ocular lobes that are more developed than in other genera, and similarly shaped eyes.

#### Host associations.

R.S. Anderson (1994: 486) reports specimens collected on the mistletoe *Struthanthus* prob. *quercicola* (Schltdl. & Cham.) D.Don (Loranthaceae).

#### Described species.

One.

#### Range.

Mexico.

### Lissoderes

Taxon classificationAnimaliaColeopteraCurculionidae

Champion, 1906b: 47

Figs 15 , 31 , 52 , 56 , 98

#### Type species.

*Lissoderes subnudus* Champion, 1906 [by original designation].

#### Gender.

Masculine.

#### Diagnosis.

*Lissoderes* is easily distinguished by its overall appearance, which is a mostly glabrous, shining body with black or reddish-brown cuticle and small patches of white scales, elongate, non-carinate, and ventrally unarmed hind femora that extend well past the abdominal apex, and an absent subapical pronotal constriction. The antennal insertion in the middle of the rostrum in females or near apex in males (Fig. 52), the second funicular article is not longer than the first, the unmodified mesoventrite is densely covered in multifid setae (Fig. 15), the premucro of the tibial apex is minute (Fig. 31), and the very narrowly bilobed third tarsal article are additional characters that in combination are unique to *Lissoderes*.

#### Keys.

Hespenheide 1987: 52.

#### Phylogenetic relationships.

Hespenheide (1987) suggests a relationship with *Arachnomorpha*. *Lissoderes*, like mentioned above with *Arachnomorpha*, are both part of a putative ant-mimicry complex (Hespenheide 1995) that includes species in other genera of Conoderine and Curculionidae with a black, glabrous cuticle and patches of white scales. The only other zygopine genera with an antennal insertion in the middle or apical half of the rostrum are *Phileas* and *Larides*, which have it near the middle of the rostrum.

#### Host associations.

*Lissoderes* is one of the few conoderine genera that have been both the subject of a taxonomic revision (Hespenheide 1987) and natural history study (Weng et al. 2007). Adults are easily found on the underside of leaves of several species of *Cecropia* and larvae feed on the parenchyma tissue inside the internodes of the stem (Weng et al. 2007).

#### Described species.

Five species are known from the focal region, including three described by Hespenheide (1987) and one by Hespenheide (2007). An additional two species are known exclusively from South America (Wibmer and O’Brien 1986: 272; one more described by Hespenheide (2007)).

#### Range.

Honduras [Hespenheide 1987], Costa Rica [Hespenheide 1987], Panama; South America.

### Peltophorus

Taxon classificationAnimaliaColeopteraCurculionidae

Schoenherr, 1845: 451

**Figures 99–102.**
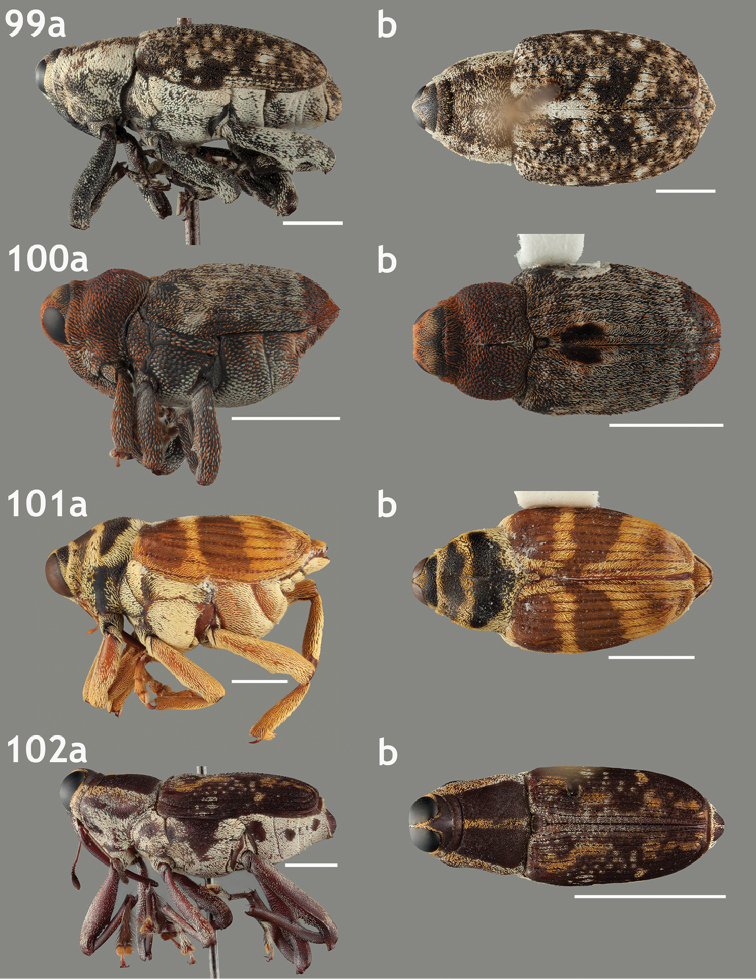
Lateral and dorsal habitus images of Zygopini. Scale bars = 2 mm unless otherwise specified. **99a–b** *Peltophorus polymitus suffusus* [ASUHIC0016837]. **100a–b** *Phileas granulatus* [ARTSYS0000528]. **101a–b** *Philenis flavipes* [ASUHIC0065102]; scale bar for 101a = 1 mm **102a–b** *Zygops vitticollis* [ASUHIC0086634]; scale bar for 102b = 5 mm.

Figs 16 , 33 , 62 , 99

= Apatorhynchus Desbrochers, 1891: 40 [Syn.: Champion 1906b: 20]. Type species: Zygops leopardinus Desbrochers, 1891 [by monotypy].
= Opalocetus Desbrochers, 1910: 126 [unjustified replacement name for Peltophorus (Champion 1910b: 211)].

#### Type species.

*Peltophorus polymitus* Boheman, 1845 [by original designation].

#### Gender.

Masculine.

#### Diagnosis.

This genus is similar only to *Zygops* with the large exposed pygidium that is visible in dorsal view and the sulcate subapical pronotal constriction, and can be readily distinguished from *Zygops* by the following characters: the shape of the eyes, which in *Peltophorus* are generally not as large and not extending as laterally on the head as in *Zygops*, the distal setal comb of the metatibia that extends more than a third to the base of the tibia (Champion 1906b: 20; Fig. 33) the femora always with one large triangular tooth (with one or several smaller teeth in *Zygops*) and the unique mesoventrite that is ventrally protruding along the posterior margin (Fig. 16), and differs from the mesoventrite of *Zygops* which is usually unmodified (or with small posterolateral processes in a few species).

#### Notes.

See Böving (1926) for description of larval and pupal morphology and González-Hernández et al. (2015) and Figueroa-Castro et al. (2016) for an overview of the feeding damage of *P. polymitus* Boheman and *P. adustus* (Fall) in Mexico.

#### Keys.

Sleeper 1963: 216 (to U.S. species), Casey 1892: 459 (to U.S. species).

#### Phylogenetic relationships.

Related to *Zygops* and the South American genera *Parazygops* and *Colpothorax* due to the exposed pygidium, large metepimeron, transverse scutellum, and sulcate subapical pronotal constriction.

#### Host associations.

The species of *Peltophorus* are associated with several species of *Agave* L. (Asparagaceae Juss.), including *Agave palmeri* Engelm. (Sleeper 1963: 216, González-Hernández et al. 2015, Figueroa-Castro et al. 2016).

#### Described species.

Three.

#### Range.

Southwestern U.S.A., Mexico, Honduras.

### Phileas

Taxon classificationAnimaliaColeopteraCurculionidae

Champion, 1906b: 34

Figs 32 , 100

#### Type species.

*Phileas granulatus* Champion, 1906 [by original designation].

#### Gender.

Masculine.

#### Diagnosis.

*Phileas* shares with *Larides* the short and thick rostrum, the antenna inserted near the middle of the rostrum, and the ocular lobes that are more produced than in other Central American Conoderinae, and can be distinguished from *Larides* by the less strongly depressed interocular space, the more completely carinate hind femora, and distinct metatibial apex (Fig. 32).

#### Notes.

Lyal et al. (2006: 214) noted the scales along the metanepisternal suture of *Phileas* as being a similar color to the regular scales surrounding the suture and thus considered them unlikely to be true sclerolepidia – a similar situation has been observed in *Larides.*

#### Phylogenetic relationships.

See above entry for Larides.

#### Host association.

One specimen observed, collected “on mistletoe” [ARTSYS0000528].

#### Described species.

One.

#### Range.

Mexico.

### Philenis

Taxon classificationAnimaliaColeopteraCurculionidae

Champion, 1906b: 43

Figs 17 , 35 , 53 , 101

#### Type species.

*Philenis flavipes* Champion, 1906 [by original designation].

#### Gender.

Feminine.

#### Diagnosis.

The short, slender antenna and narrow, acuminate club are given by Champion (1906b: 43) to distinguish the genus from *Copturus* (=*Macrocopturus*), which also separate it from the rest of the genera except for the observed South American specimens of *Hypoplagius*. *Philenis flavipes* has an unmodified mesoventrite but *P. fuscofemorata* Champion, 1906 and three observed undescribed species have a large tubercle at the posterolateral margins of the mesoventrite with the posteromedial margin being strongly depressed (Fig. 17). The two described species are easily distinguished for being the only known members of the putative bee mimicry complex (Hespenheide 1995: 150) with the contrasting yellow and black or brown pattern, but some observed undescribed species are not, with one being a representative of the “red-eyed fly” mimicry complex. The second funicular article is longer than the first, eyes are ovoid and somewhat protruding (Fig. 53), and the femora are non-carinate and ventrally toothed.

#### Notes.

Champion (1906b: 44) notes that the pygidium is slightly exposed in *P. flavipes*, but all observed specimens of that species have the last abdominal ventrites deflected downwards (i.e. not in their natural position during life) so this character has not been confirmed. *Philenis* was not moved to Lechriopini by Lyal et al. (2006) despite having some type of modification to the mesoventrite because of a lack of sclerolepidia. The two described species, *P. flavipes* and *P. fuscofemorata*, lack sclerolepidia, however, observed specimens of three congeneric undescribed species do have sclerolepidia. The genus is not moved to the Lechriopini here due to the lack of a suitably identified sister genus.

#### Phylogenetic relationships.

*Philenis* was regarded by Champion (1906b: 43) to be “closely related to *Copturus* [=*Macrocopturus*] in its restricted sense”, but the genus is not very similar in appearance to others in the Lechriopini or Zygopini. Interestingly, both the reported host association and the mesoventrite, which in some species has a deep semicircular depression, are similar to that found in *Hoplocopturus* and *Mnemynurus*.

#### Host associations.

One species has been collected from a “gall on an aroid stem” (Hespenheide 1995: 150).

#### Described species.

Two.

#### Range.

Costa Rica, Panama.

### Zygops

Taxon classificationAnimaliaColeopteraCurculionidae

Schoenherr, 1825: c.586

Figs 34 , 102

= Eccoptus Dejean, 1821: 86 [Syn.: O’Brien and Wibmer 1984: 296]. Type species: Curculio strix Olivier, 1790 [by monotypy]. Suppressed for priority (ICZN 1987).
= Eccyptus [Fischer von Waldheim], 1829: 99. Type species: Curculio strix Olivier, 1790 [by monotypy].

#### Type species.

*Poecilma wiedii* Germar, 1824.

#### Gender.

Masculine.

#### Diagnosis.

Of the genera occuring north of South America, *Zygops* could be mistaken only with *Peltophorus* with the large, exposed pygidium that is mostly visible in dorsal view and deflecting the fifth abdominal ventrite ventrally at the apex, and the sulcate subapical constriction of the prothorax. *Zygops* can be differentiated from *Peltophorus* by the shorter metatibial setal comb (Fig. 34), the much longer second funicular article (usually at least 2 times longer in *Zygops*, 1.5-2 times longer in *Peltophorus*), the metafemora ventrally with more than one tooth (in many species), the more elongate tibial uncus (Fig. 34), and the less developed mesoventrite, which in *Zygops* is usually flattened, but in some with the posterolateral margin tumescent (e.g. *Z. maculipes* Desbrochers, 1891) or with small projections (e.g. the South American *Z. leucogaster* Desbrochers, 1891). The prosternal channel is sometimes very narrow and scarcely depressed, and the procoxae are sometimes very narrowly separated or even contiguous (e.g. in *Z. maculipes*).

#### Notes.

The genus *Eccoptus* Dejean, 1821 was suppressed despite having priority over *Zygops* Schoenherr, 1825 (petitioned in O’Brien and Wibmer 1986, ruled by ICZN 1987) for the purpose of nomenclatural stability: the name *Eccoptus* had been used much less frequently (though most recently resurrected in O’Brien and Wibmer 1982) and the name *Zygops* formed the base for the subfamilial name in use at the time, Zygopinae.

#### Keys.

Champion 1906b: 21.

#### Phylogenetic relationships.

See *Peltophorus*. Most similar to *Peltophorus* of the genera treated here, but the distinction between *Zygops* and the South American genera *Parazygops* and *Colpothorax* is less distinct, based on the shape of the rostrum and the shape of the prothorax, respectively.

#### Host associations.

Some species have been reared from branches of various genera of Lecythidaceae (Fassbender 2013, Fassbender et al. 2014).

#### Described species.

Eighteen species are known from the focal region and an additional 34 species are known only from South America (Wibmer and O’Brien 1986: 267).

#### Range.

Mexico, Guatemala, Belize, Honduras, Nicaragua, Costa Rica, Panama, Dominican Republic (Poinar and Legalov 2013, fossil); South America.

### Zygopsella

Taxon classificationAnimaliaColeopteraCurculionidae

Champion, 1906b: 42

Figs 54 , 103

#### Type species.

*Zygopsella ruficauda* Champion, 1906 [by original designation].

#### Gender.

Feminine.

**Figures 103–105.**
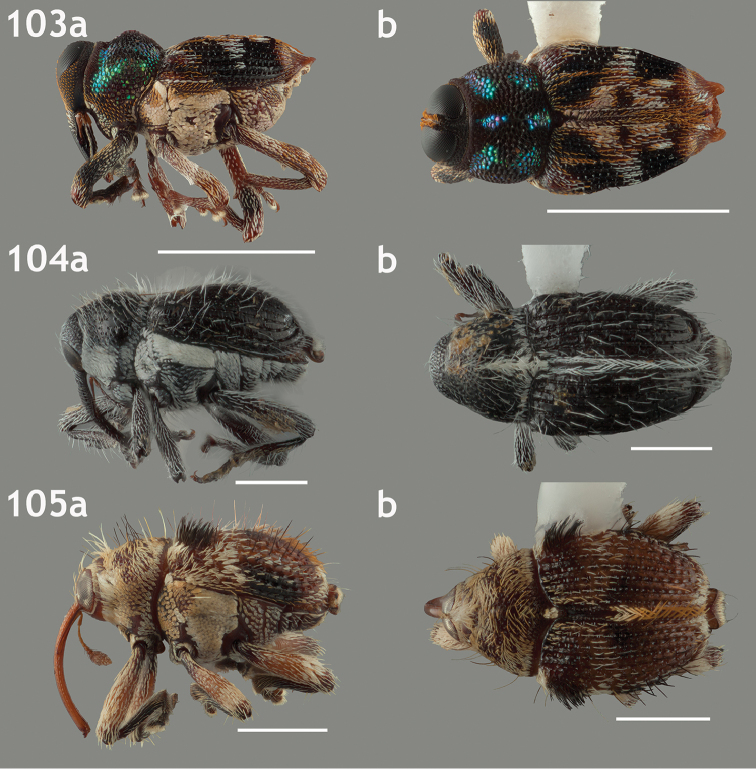
Lateral and dorsal habitus images of Zygopini and Conoderinae *incertae sedis*. Scale bars = 1 mm unless otherwise specified. **103a–b** *Zygopsella ruficauda* [ARTSYS0000526]; scale bar = 2 mm **104a–b** *Philides comans* [ARTSYS0000804] **105a–b** *Philinna bicristata* [ARTSYS0000532].

#### Diagnosis.

*Zygopsella* is similar in appearance to *Archocopturus* and some species of *Macrocopturus*, *Hoplocopturus*, and *Copturomimus* with blue-green pronotal scales, but of those genera is similar only to *Archocopturus* with the subequal first two articles of the funiculus and deep pronotal punctures. From *Archocopturus*, *Zygopsella* can be separated by the more widely separated eyes (Fig. 54), the more sharply acuminate lower margin and more strongly inflexed lower lateral margin of the eyes, the more strongly arcuate lateral margins of the pronotum (Fig. 103b), the ventrally toothed profemora, the ventrally emarginate tibiae to receive the femoral tooth, and the flattened caudal prominences of the elytra (Fig. 103b).

#### Phylogenetic relationships.

Suggested by Champion (1906b: 42) to be closely related to *Archocopturus*. The combination of ascending abdominal ventrites, concealed pygidium, unmodified mesoventrite, carinate and ventrally toothed metafemora and short second funicular article of the antenna is shared only with *Archocopturus*, *Phileas*, and *Larides* (the latter only with a faint carina in the distal half of the femora). Also similar to many *Cylindrocopturus* with the elytral processes and relatively widely separated eyes. *Zygopsella* has a similar eye shape to *Helleriella* that is strongly laterally inflexed and sharply ventrally acuminate.

#### Host associations.

Unknown.

#### Described species.

Two, including one species transferred from *Archocopturus* by Hespenheide (2005).

#### Range.

Mexico (Hespenheide 2005: 683), Guatemala, Costa Rica (Hespenheide 2005: 682), Panama (Hespenheide 2005: 683), Guadeloupe (Hespenheide 2005: 683).

### Conoderinae *incertae sedis*

#### Philides

Taxon classificationAnimaliaColeopteraCurculionidae

Champion, 1906b: 129

Figs 59 , 104

##### Type species.

*Philides anthonomoides* Champion, 1906 [by monotypy].

##### Gender.

Masculine.

##### Diagnosis.

*Philides* is easily differentiated from all other genera treated here except *Philinna* by the following characteristics: the body, especially the lateral surfaces, are densely covered in multifid setae; the antennal funiculus has 6 instead of 7 articles (as in Fig. 57 for *Philinna*); the tibial apex does not have an uncus at the posterior apical angle and has either a small process at inner apical angle (as in Fig. 36 for *Philinna*) or no process at all; the tibial apex is subcircular in cross-section (not laterally compressed as in all other genera) and with distinct fringe of spine-like setae around the apex; the prosternum behind the procoxae has ventrally projecting laterally compressed tubercles (as in Fig. 18 for *Philinna*); the mesoventrite is vertical, unmodified or with posterolateral margins modified into somewhat projecting lamellae (as in Fig. 18); the first elytral interval has elongate, stout setae crossing over the suture in roughly posterior half (Fig. 104b); and the fifth abdominal ventrite is strongly emarginate to accommodate the exposed pygidium. *Philides* differs from *Philinna* in the presence of tarsal claws with a broad tooth (Fig. 59; seen in *Philides comans* Champion, 1909, but not all observed specimens identified to *P. anthonomoides* had this tooth) and in overall appearance, with the known species of *Philides* belonging to the “shiny black” mimicry complex of Hespenheide (1995: 149). Some observed specimens identified as *P. anthonomoides* have the mesoventrite unmodified, with slight projections at the posterolateral margins or with the mesoventrite strongly excavated apically to receive the rostrum.

##### Phylogenetic relationships.

The numerous characters that differentiate this genus and *Philinna* from the rest of the conoderines treated here suggest improper placement in Conoderinae, as suggested by Lyal et al. (2006) due to the lack of sclerolepidia and differently constructed mesoventrite. Champion (1906: 130) and Hespenheide (1992: 2, 2002: 756) noted the similarity of these genera with Tachygonini (a group sometimes included in the Conoderinae but since relegated to a subtribe in the Curculioninae: Rhamphini, treated there most recently in Caldara et al. 2014) in having multifid setae covering much of the body. Further similarities of these three genera are the stout, crossed setae along the elytral suture. Tachygonines, however, have a more conoderine-like tibial apex than either *Philides* and *Philinna*, being laterally compressed and with an uncus at the posterior apical angle. Specimens were not observed for two of the three genera currently placed in the Tachygonina, reserving a reconsideration of the placement of the subtribe for future phylogenetic study. *Philides* also bears a resemblance to the Old World conoderine tribe Lobotrachelini, but the observed species of that tribe also differ from *Philides* and *Philinna* in having a more typical conoderine tibial apex. The only other genus treated here without simple tarsal claws is *Psomus* Casey, which does not have a similar broad, flat tooth as in *Philides*.

##### Host associations.

The larva of at least one species is an inquiline in galls made by a buprestid (Medianero et al. 2007).

##### Described species.

Two. Numerous undescribed species and related genera occur in Central America (H. Barrios, personal communication). One additional described species is known from South America (Rheinheimer 2011).

##### Range.

Mexico, El Salvador, Panama; South America (Rheinheimer 2011).

#### Philinna

Taxon classificationAnimaliaColeopteraCurculionidae

Champion, 1906b: 128

Figs 18 , 36 , 57 , 105

##### Type species.

*Philinna bicristata* Champion, 1906 [by monotypy].

##### Gender.

Feminine.

##### Diagnosis.

*Philinna* can be distinguished from the rest of the genera treated here by characters listed above for *Philides*. It differs from most *Philides* by the following characters: the tarsal claws are simple, the pronotum has a smooth, raised median line, and the elytral humeral angle has dense tufts of elongate setae.

##### Phylogenetic relationships.

Very similar to *Philides* but its relationship to other genera is currently uncertain. See *Philides*.

##### Host associations.

Unknown.

##### Described species.

One species is known from the focal region and one additional species is known in South America (Wibmer and O’Brien 1986: 265).

##### Range.

Mexico (Wibmer and O’Brien 1989: 19), Belize [ARTSYS0000799], Guatemala, Panama [ARTSYS0000806]; South America.

### Identification resources

*Previous regional keys to genera*. The following references provide the publication and page number of a published identification key treating genera from North and/or Central America, the Caribbean, and South America (if they also include genera whose ranges extend to Central America). An asterisk (*) indicates publication in a language other than English.

Blatchley and Leng 1916: 417 (Northeastern U.S.A.), Casey 1897: 667 (U.S.A.), Gluck 1987: 8 (Zygopini north of Mexico), Hatch 1971: 361 (Northwestern U.S.A.), *Heller 1984: 3 (World), *Heller 1895: 3 (New World), *Heller 1906: 31 (New World Piazurini), Hespenheide 2002: 754 (U.S.A.), *Hustache 1932a: 275 (Guadeloupe), Kissinger 1964: 71 (U.S.A.), LeConte and Horn 1876: 259 (U.S.A.), *Muñiz 1965:5 (avocado pests), *Muñiz and Barrera 1958 (avocado pests), *Rheinheimer 2011: 72 (French Guiana), Sleeper 1963: 209 (U.S.A.).

*Key to genera from focal region.* The genera treated by the below key are listed synoptically in Table 1 along with all genera included in the five tribes that are not from the focal region (as indicated by an asterisk or circumflex accent). This key incorporates elements from previous keys, characters from the original descriptions of genera, as well as many new characters. It will not necessarily work for species outside of the focal range or for undescribed species from the focal range, but as many of those species as possible were worked in. The sequence of the following key is approximately in perceived phylogenetic order with pragmatic deviations to allow for more efficient identification.

**Table:** 

1	Antennal club loose and bearing elongate setae (Fig. 67b). Procoxae contiguous. Rostral channel prosternally with the sides converging to a point ventral to the procoxae (Fig. 1)	***Trichodocerus***
–	Antenal club compact and with short setae (Figs 55–57). Procoxae separate (can be very narrowly separated or rarely contiguous – if contiguous, antennal club always compact). Rostral channel on the prosternum not terminating below the coxae (e.g. Fig. 10)	**2**
2	Pygidium broadly exposed (if only narrowly exposed, mesoventrite a cup-shaped receptacle for receiving rostrum as in Fig. 2)	**3**
–	Pygidium completely concealed or only very narrowly exposed (if narrowly exposed, mesoventrite not a cup-shaped receptacle for receiving rostrum)	**11**
3	Antennal funicle composed of 6 articles (Fig. 57). Tibial apex without uncus at posterior apical angle (Fig. 36). Lateral and dorsal surfaces of the body in large part covered in multifid setae. First elytral interval with elongate and stout setae that cross over elytral suture in posterior half (Figs 104b, 105b)	**4**
–	Antennal funicle composed of 7 articles (Fig. 56). Tibial apex with uncus at posterior apical angle (Figs 22–35). If body bearing multifid setae then not covering large portions of the lateral and dorsal surfaces. Elytral interval 1 without stout setae crossing over elytral suture	**5**
4	Tarsal claws often with a broad tooth at the base (Fig. 59). Body in large part glabrous, cuticle black and with white setae/scales (Fig. 104)	***Philides***
–	Tarsal claws simple. Cuticle black and reddish brown (Fig. 105). Setal color various	***Philinna***
5	Subapical constriction of pronotum sulcate (especially when viewed dorsally as in Fig. 99b). Exposed portion of pygidium mostly to entirely visible in dorsal view (Figs 99b, 102b). Meso- and/or metafemoral apices with teeth at mesal and/or lateral face (Fig. 62). Scutellum usually distinctly quadrate (Fig. 102b) or transversely rectangular	**6**
–	Subapical constriction of pronotum shallow or absent. Exposed portion of pygidium mostly to entirely concealed in dorsal view (Figs 68b–72b) and visible in posterior or ventral view only in most. Femoral apices without teeth (Fig. 60). Scutellum variable but never distinctly quadrate or transversely rectangular	**7**
6	Longitudinal setal comb of posterior distal face of hind tibia occupying distal half (Fig. 33). Posterior margin of mesoventrite ventrally produced (Fig. 16). Tibial uncus very short (Fig. 33). Femora never armed ventrally with more than one tooth	***Peltophorus***
–	Longitudinal setal comb of posterior distal face of hind tibia occupying distal third or less (Fig. 34). Posterolateral margin of metaventrite of most flattened but in few slightly tumescent or with small processes. Tibial uncus variable but not as above (Fig. 34). Femora armed ventrally with one to several teeth	***Zygops***
7	Antennal funicular article 2 usually about equal in length to article 1 (as in Fig. 56). Metafemora slightly clavate, ventral tooth present and not especially large or laterally flattened	**8**
–	Antennal funicular article 2 usually at least 2 times longer than article 1 (as in Fig. 55). Metafemora clavate, with large laterally flattened tooth (Fig. 71a)	**9**
8	Rostral channel closed on the mesoventrite (Fig. 2). Eyes more widely separated dorsally and strongly concave between (Fig. 39). Setal tuft at tibial apex composed of a few golden setae (as in Fig. 23). Setal comb of meso- and metafemora (along posterodistal face) not a dense brush, composed of no more than a few rows of setae. Profemora ventrally with two projections, a pointed tooth near the middle and a smaller, rounded prominence distally	***Lobops***
–	Rostral channel open on the mesoventrite (as in Figs 3–4). Eyes large and approximate or small and widely separated along entire length (Fig. 38), at most slightly concave between. Setal tuft at tibial apex a thick fascicle of golden setae (Fig. 21). Setal comb of meso- and metafemoral apex a broad, dense setal brush (Fig. 21). Profemora ventrally with one or no teeth (rarely two)	***Cratosomus***
9	Abdominal ventrite 2 at the side about as long as 3 and 4 combined (Fig. 70a). Pro- and mesotibial apices with premucro. Premucro of metatibial apex never subapical	***Piazurus***
–	Abdominal ventrite 2 at the side shorter than 3 and 4 combined (Figs 71a, 72a). Pro- and mesotibial apices without premucro. Premucro of metatibial apex sometimes subapical	**10**
10	Eyes very large, ovoid and contiguous (Fig. 40). Pronotum conical in dorsal view (Fig. 71b). Mesepipleura large and ascending. Profemora unarmed ventrally. Abdominal ventrite I with two arcuate sulci (Fig. 64). Vertex of head without arcuate carina	***Pseudopiazurus***
–	Eyes smaller, more circular, subcontiguous or more separated (Fig. 41). Pronotum (Fig. 72b) and mesepipleura usually not as above. Profemora ventrally toothed (in most). Vertex of head with arcuate carina (Fig. 41; in many Central American species). Abdominal ventrite I without large U-shaped impression, or if present, then vertex of head always with arcuate carina	***Pseudopinarus***
11	Scutellum partially or completely concealed by posteriorly projecting medial lobe of pronotum (Fig. 76b)	**12**
–	Scutellum completely exposed	**14**
12	Mesoventral channel open (Fig. 8). Eyes vertical and widely separated. Excavation to metaventrite anterior to metacoxa with large tubercle (Fig. 65)	***Euzurus***
–	Rostral channel closed on the mesoventrite (Fig. 6). Eyes not as above. Metaventrite without large tubercle anterior to metacoxa	**13**
13	Pro- and mesofemora unarmed ventrally. Tarsal claws very small (Fig. 58). Elytral intervals costate	***Microzurus***
–	Pro- and mesofemora armed ventrally. Tarsal claws normal. Elytral intervals not costate	***Copturus***
14	Mesoventrite with a single arcuate carina in the shape of an inverted “U” (Fig. 9)	**15**
–	Mesoventrite with or without carinae, but if present not in the shape of an inverted “U”	**16**
15	Metaventrite with complete longitudinal depression or channel (in most). Rostrum very long, extending beyond posterior margin of metaventrite (Fig. 87a; except in *M. longispinis*). Apex of rostrum flattened and dilated. Posterior margin of mesoventrite invaginated under U-shaped carina (as in Fig. 9)	***Mnemynurus***
–	Metaventrite, if modified, with depression or fovea limited mainly to the anterior, intermesocoxal region or the middle of the sclerite and never from the anterior to the posterior border. Rostrum not extending beyond posterior margin of metaventrite (Fig. 81a). Rostral apex not significantly depressed or dilated. Posterior margin of mesoventrite usually depressed (in most) or invaginated (in few; Fig. 9)	***Hoplocopturus***
16	Rostral channel of mesoventrite with longitudinal or slightly arcuate carinae (e.g. Figs 10, 13). Posterior margin of mesoventrite without ventrally produced tubercles	**17**
–	Mesoventrite without carinae, or, if bearing carinae, then the posterolateral margins also tuberculate	**25**
17	Hind femora slender and elongate, extending well beyond abdominal apex (Fig. 90a). Rostral channel a deep ovoid receptacle (Fig. 13)	***Pseudolechriops***
–	Hind femora stout, not extending much past abominal apex if at all. Rostral channel not as above	**18**
18	Femora ventrally toothed. Funicular article 2 longer than article 1	**19**
–	Femora ventrally unarmed. Funicular article 2 not longer than article 1	**20**
19	Metafemora not carinate (as in Fig. 61). Body size > 5 mm	***Poecilogaster***
–	Metafemora carinate (as in Fig. 62). Body size usually < 5 mm	***Lechriops***
20	Profemora dorsally with bare, finely striolate region (as in Fig. 66 or more concealed)	***Copturomorpha***
–	Profemora without striolate area	**21**
21	Pronotum with strongly arcuate, hump-like medial longitudinal carina (Fig. 84a)	***Macrolechriops***
–	Pronotum without hump-like carina	**22**
22	Hind femora and tibia of males arcuate and bearing erect setae ventrally (Fig. 77a). Procoxae without mesal conical process	***Coturpus***
–	Hind femora not elongate and arcuate. Procoxae mesally with small conical process	**23**
23	Apex of rostral channel without distinct termination (apex is the non-carinate anterior margin of the metaventrite) (Fig. 7)	***Cylindrocopturinus***
–	Rostral channel terminating on either mesoventrite or metaventrite, carinate or not, but always being a distinctly posteriorly rounded receptacle	**24**
24	Rostral channel ending on mesoventrite in deep carinate cup-like receptacle (Fig. 14). On mistletoe (Santalaceae: *Phoradendron*)	***Turcopus***
–	Rostral channel, if ending on the mesoventrite, not a deep, carinate receptacle. Hosts various	***Eulechriops***
25	Mesoventrite with some prominent modification at least in posterior half in the form of tubercles, carinae or depressions (if unmodified, antenna also very short and slender) (Figs 11, 12, 17)	**26**
–	Mesoventrite without the abovementioned modification in posterior half (Figs 5, 15), with posterolateral corners at most slightly tumescent; antenna not short and slender	**29**
26	Antenna short and slender (Fig. 53). Posterolateral margin of mesoventrite (in most) with tubercles and a deep semicircular depression (Fig. 17). Tibial apex with premucro directed at a roughly 45° angle from the longitudinal axis of the tibia (Fig. 35). Eyes ovoid and somewhat protuberant on head (Fig. 53)	***Philenis***
–	Posterolateral margin of mesoventrite elevated, forming a “platform” for the rostrum to rest on, usually with tubercles at the posterolateral margin and otherwise lacking the above combination of characters	**27**
27	Posterior margin of metaventrite with short transverse carina marking end of rostral channel (Fig. 12). Region of mesoventrite anterior to posterior tubercles without carinae	***Paramnemyne***
–	Posterior margin of metaventrite without a short transverse carina. Mesoventrite with medial depression delimited by slightly arcuate longitudinal carinae	**28**
28	Hind femora slender and elongate, extending well beyond the apex of the abdomen (Fig. 86). Hind femora not carinate and, if ventrally toothed, tooth small and inconspicuous	***Microzygops***
–	Hind femora not extending much beyond the apex of the abdomen, carinate and with distinct ventral tooth	***Macrocopturus*** [few]
29	Tarsal claws appendiculate. Subapical pronotal constriction sulcate	***Psomus***
–	Tarsal claws simple. Subapical pronotal constriction, if present, not sulcate	**30**
30	Abdominal sternites slightly evenly ascending in profile. Tibiae lacking premucro (Fig. 24)	***Acoptus***
–	Abdominal sternites strongly ascending in profile. Hind tibia at least with premucro	**31**
31	Article 2 of antennal funicle not longer than article 1 (as in Fig. 56)	**32**
–	Article 2 of antennal funicle much longer than article 1 (as in Fig. 55)	**39**
32	Antennal insertion clearly in basal half of rostrum (usually basal third)	**33**
–	Antennal insertion near middle or in apical half of rostrum	**37**
33	Hind femora carinate, never with paired tubercles on the second abdominal ventrite	**34**
–	Hind femora not carinate, or if carinate also with paired tubercles on the second abdominal ventrite	**36**
34	Hind femora ventrally unarmed. Body of known species with cuticle shining black and vestiture composed of opalescent white scales (Fig. 93)	***Arachnomorpha***
–	Hind femora armed ventrally with 1 tooth. Pronotum of known species with blue-green scales (Figs 94, 103)	**35**
35	Profemora armed ventrally with 1 tooth. Elytral apex with flattened processes (Fig. 103b). Vertex of head without triangular, transversely striolate region	***Zygopsella***
–	Profemora ventrally unarmed. Elytral apex without flattened processes. Vertex of head with triangular, transversely striolate region (visible in Figs 48, 94b)	***Archocopturus***
36	Vestiture consisting of linear scales (Fig. 96). Tibial apices with very short, curved uncus (Fig. 30). Eyes acuminate ventrally and strongly inflexed laterally towards the bottom (Fig. 50). Elytra and mesoventrite never tuberculate	***Helleriella***
–	Vestiture consisting of overlapping, rounded scales at least in part, densely covering most of body surface (in many species). Tibial apex (at least of protibia) usually with hook-like uncus and a produced, rounded inner flange (Fig. 29). Eyes acuminate ventrally and rounded at sides, often vertical and somewhat separated (Fig. 49). Elytra often tuberculate or with erect tufts of scales. Second abdominal ventrite of some with paired tubercles	***Cylindrocopturus***
37	Ocular lobes absent (Fig. 98a). Hind femora extending well beyond apex of abdomen (Fig. 98). Subapical pronotal constriction absent (Fig. 98b). Eyes not widely separated at the top and not strongly depressed in between (Fig. 52)	***Lissoderes***
–	Ocular lobes slightly produced (Figs 97a, 100a). Hind femora shorter and more stout (Figs 97a, 100a). Subapical pronotal constriction present. Eyes at the top widely separated and the interocular space strongly depressed (Fig. 51)	**38**
38	Metafemora completely carinate. Metatibial apex with premucro oriented at a ~45° angle to longitudinal axis of tibia (Fig. 32)	***Phileas***
–	Metafemora with faint carina in distal half. Metatibial apex with premucro oriented along longitudinal axis of tibia (as in Fig. 28)	***Larides***
39	Profemora dorsally with a denuded, striolate patch (Fig. 66). Femora with a single ventral tooth	***Copturomimus***
–	Profemora dorsally without a denuded, striolate patch. Some species with more than one ventral femoral tooth	***Macrocopturus*** [most]

## Discussion

The monophyly of the Conoderinae as well that of its tribes and genera have not yet been demonstrated. While the present study is not considered comprehensive enough in both taxon and character system sampling to provide a significantly emended classification of the Conoderinae, it provides a first summary of phenotypic information for many of the treated genera and tribes and an examination of the phylogenetic utility of several morphological character systems that have been traditionally used to define taxa. This has revealed several suspected classificatory changes that will be needed to achieve a phylogenetic classification.

Many genera as they are currently constructed can only be identified by a combination of characters and by negative identification of similar genera, and numerous specimens have been examined that lack part of the character combinations and appear intermediate between genera. Several new genera will likely be created from those that are currently large and unsatisfactorily delimited. Additional character systems, such as the genitalia, are likely to provide more clarity to hypotheses of generic monophyly and relationships as many of the external characters traditionally used have been found to be limited for these purposes. Such characters, like the length of the funicular articles and the presence of a carina or tooth on the hind femora, can be useful at the generic level when used in combination but are certainly not without exception in the larger genera.

Of the characters traditionally influential for conoderine classification, the mesoventrite remains one of the most useful for identification at the level of genus as this structure is relatively invariable within most genera. However, its utility at higher levels, especially that of the tribal level, does not seem to be as originally implicated by Lacordaire (1865). The distribution of the types of modification to the mesoventrite within the current classificatory framework suggests that this is a very homoplasius character system, with certain types appearing independently in multiple lineages (e.g. multiple transitions from unmodified to modified), or that the different types are homologous and the tribes, as currently composed, contain many improperly placed genera. Both, to some degree, are likely to be true, but with limited current knowledge of relationships it is difficult to assign polarity to the different states of modification.

The tibial apex of New World Conoderinae conforms to a general structure that varies little, with the exception of the production of the inner flange, which can be quite variable within a genus. Three of the four genera transferred out of the Lechriopini in this paper are genera that deviated most from this general structure, with *Acoptus* having a tibial apex more similar to Old World Conoderinae and *Philinna* and *Philides* having a tibial apex distinct among the observed Conoderinae and likely indicative of a proper placement elsewhere in the Curculionidae. Undoubtedly, much work remains to be done in circumscribing the New World conoderine tribes and genera and elucidating their phylogenetic relationships, but it is hoped that this contribution to conoderine systematics can provide the foundation to facilitate such studies in the future.

## Supplementary Material

XML Treatment for Conoderinae

XML Treatment for Trichodocerini

XML Treatment for Trichodocerus

XML Treatment for Piazurini

XML Treatment for Cratosomus

XML Treatment for Lobops

XML Treatment for Piazurus

XML Treatment for Pseudopiazurus

XML Treatment for Pseudopinarus

XML Treatment for Othippiini

XML Treatment for Acoptus

XML Treatment for Lechriopini

XML Treatment for Copturomimus

XML Treatment for Copturomorpha

XML Treatment for Copturus

XML Treatment for Coturpus

XML Treatment for Cylindrocopturinus

XML Treatment for Eulechriops

XML Treatment for Euzurus

XML Treatment for Hoplocopturus

XML Treatment for Lechriops

XML Treatment for Macrocopturus

XML Treatment for Macrolechriops

XML Treatment for Microzurus

XML Treatment for Microzygops

XML Treatment for Mnemynurus

XML Treatment for Paramnemyne

XML Treatment for Poecilogaster

XML Treatment for Pseudolechriops

XML Treatment for Psomus

XML Treatment for Turcopus

XML Treatment for Zygopini

XML Treatment for Arachnomorpha

XML Treatment for Archocopturus

XML Treatment for Cylindrocopturus

XML Treatment for Helleriella

XML Treatment for Larides

XML Treatment for Lissoderes

XML Treatment for Peltophorus

XML Treatment for Phileas

XML Treatment for Philenis

XML Treatment for Zygops

XML Treatment for Zygopsella

XML Treatment for Philides

XML Treatment for Philinna
